# The evolution of YidC/Oxa/Alb3 family in the three domains of life: a phylogenomic analysis

**DOI:** 10.1186/1471-2148-9-137

**Published:** 2009-06-18

**Authors:** Yu-Juan Zhang, Hai-Feng Tian, Jian-Fan Wen

**Affiliations:** 1 State Key Laboratory of Genetic Resources and Evolution, Kunming Institute of Zoology, Chinese Academy of Sciences, Kunming, Yunnan Province 650223, PR China; 2 Graduate School of the Chinese Academy of Sciences, Beijing 100039, PR China

## Abstract

**Background:**

YidC/Oxa/Alb3 family includes a group of conserved translocases that are essential for protein insertion into inner membranes of bacteria and mitochondria, and thylakoid membranes of chloroplasts. Because mitochondria and chloroplasts are of bacterial origin, Oxa and Alb3, like many other mitochondrial/chloroplastic proteins, are hypothetically derived from the pre-existing protein (YidC) of bacterial endosymbionts. Here, we test this hypothesis and investigate the evolutionary history of the whole YidC/Oxa/Alb3 family in the three domains of life.

**Results:**

Our comprehensive analyses of the phylogenetic distribution and phylogeny of the YidC/Oxa/Alb3 family lead to the following findings: 1) In archaea, YidC homologs are only sporadically distributed in Euryarchaeota; 2) Most bacteria contain only one YidC gene copy; some species in a few taxa (*Bacillus*, Lactobacillales, Actinobacteria and Clostridia) have two gene copies; 3) Eukaryotic Oxa and Alb3 have two separate prokaryotic origins, but they might not arise directly from the YidC of proteobacteria and cyanobacteria through the endosymbiosis origins of mitochondrium and chloroplast, respectively; 4) An ancient duplication occurred on both Oxa and Alb3 immediately after their origins, and thus most eukaryotes generally bear two Oxa and two Alb3. However, secondary loss, duplication or acquisition of new domain also occurred on the two genes in some lineages, especially in protists, resulting in a rich diversity or adaptive differentiation of the two translocases in these lineages.

**Conclusion:**

YidC is distributed in bacteria and some Euryarchaeota. Although mitochondrial Oxa and chloroplastic Alb3 are derived from the prokaryotic YidC, their origin might be not related to the endosymbiosis events of the two organelles. In some eukaryotic lineages, especially in protists, Oxa and Alb3 have diverse evolutionary histories. Finally, a model for the evolutionary history of the entire YidC/Oxa/Alb3 family in the three domains of life is proposed.

## Background

Bacterial YidC, eukaryotic Oxa (in mitochondria) and Alb3 (in chloroplasts) form an evolutionarily conserved protein translocase family, which plays an essential role in protein inserting into inner membranes of bacteria and mitochondria, and thylakoid membranes of chloroplasts. Proteins translocated by them, regardless of nuclear-encoded or organelles-encoded, are mainly respiration- and other energy transduction-involved components [1,2]. Because mitochondria and chloroplasts are derived from a proteobacterial and a cyanobacterial endosymbiont, respectively, Oxa and Alb3 are supposed to have originated from prokaryotic YidC and they might have played an important role in establishing a tight relationship between the endosymbionts and the host cell. Actually, it has been suggested that, in the course of transforming from a proteobacterial endosymbiont to mitochondria, some of the pre-existing protein translocation apparatus of the endosymbiont appears to have been commandeered, with molecular chaperones such as mHsp70 and Oxa1 derived from the bacterial chaperones DnaK and YidC, respectively [3]. However, whether Oxa and Alb3 really evolutionarily originated directly from the YidC of the bacterial progenitors of mitochondria and chloroplasts, or inherited vertically from the YidC of the prokaryotic ancestors of eukaryotes, or arose by other means, remains an open question.

In archaea, some proteins distantly related to YidC/Oxa/Alb3 were considered to be members of this family in some previous studies [1,2]. However, because of their low sequence similarities, whether these archaeal sequences really belong to the YidC/Oxa/Alb3 family needs to be further confirmed. In bacteria, one YidC gene copy has been reported in most species studied; only a few taxa, including *Bacillales *and Lactobacillales, reportedly possess two YidC genes [1,2]. Two Oxa versions are found in eukaryotes: Oxa1, the first identified Oxa, is required for the assembly of the respiratory-chain protein subunits into mitochondria inner membrane [4,5] whereas Oxa2 (or Cox18) provides a complementary role to Oxa1 in the assembly of cytochrome oxidase. The two Oxa were considered to have evolved from a duplication of their ancestral gene during the early evolution of mitochondria [6,7]. Because this evolutionary scenario was deduced only from the studies on higher eukaryotes, further investigations are necessary to include lower eukaryotes such as protists, which represent the early stage of eukaryote evolution. As for chloroplastic Alb3, a few plastid-containing eukaryotes have been investigated thus far. Two members of Alb3 were identified in *Chlamydomonas reinhardtii *and *Arabidopsis thaliana*; one is proved to be essential for the efficient assembly of subunits of photosynthetic complexes [8,9] and the other has a special function in proper chloroplast biogenesis besides more or less involvement in assembling photosynthetic complexes [10,11]. Nevertheless, whether all plastid-containing eukaryotes possess the two Alb3 members is still unclear and their evolutionary history is unknown yet.

Earlier phylogenetic analysis of Oxa1 family performed by Yen et al [2] included only a very limited number of sequences and taxonomic samples. Until now, no studies on the evolution of the entire YidC/Oxa/Alb3 family in all the three domains of life are reported.

To address the above issues, we conducted comprehensive searches against all available public databases for YidC/Oxa/Alb3 homologs. We further investigated the phylogenetic distribution of the family in the three domains of life and performed phylogenetic analyses using maximum likelihood and neighbor-joining approaches. An evolutionary route chart of the entire YidC/Oxa/Alb3 family is proposed mainly based on the results of our study.

## Results

### Phylogenetic distribution of YidC/Oxa/Alb3 in the three domains of life

Of the 45 archaeal species investigated, no YidC homologs were found in all the 13 Crenarchaeota and one Nanoarchaeota. YidC homologs were only found in seven of the 31 Euryarchaeota, each containing a single copy (see Additional file 1) with low sequence similarity to those from bacteria. Nevertheless, though their sequences are usually short (174~308aa), our prediction with Tmpred showed these Euryarchaeota YidC homologs all possess four transmembrane regions (see Additional file 2). Among these homologs, only two (15790660 and 15668657) had been reported previously [2].

We have investigated 589 bacterial species which cover almost all the lineages (see Additional file 1). As previous work [1,2] showed, one YidC homolog was identified in most species and two in many *Bacillus *and Lactobacillales. For the first time, we found that many Actinobacteria and one *Clostridium *also have two YidC genes. Interestingly, all the bacteria with two YidC homologous genes are generally Gram-positive bacteria, but not every Gram-positive bacterium possesses two YidC genes. These bacterial YidC have various sizes ranging from 249 to 794 aa, but our prediction with Tmpred showed that they all possess several (5–6) transmembrane regions (see Additional file 2).

In eukaryotes, two Oxa (Oxa1 and Oxa2) homologs can be found in fungi and metazoa, which is consistent with the previous investigation of fewer species [7]. However, we found much more distributional diversity in other lineages. Firstly, the number of Oxa homologs varies among protists: two copies are found in *Monosiga brevicollis*, *Trypanosoma *and *Leishmania*, with the exception that *T. cruzi *contain three copies. Only one copy was found in *Plasmodia *and none in amitochondriate protozoa [12], including *Giardia lamblia*, *Trichomonas vaginalis *(both of which had been reported to have no Oxa, previously [13]), *Cryptosporidium hominis*, *C. parvum*, *Encephalitozoon cuniculi*, and *Entamoeba histolytica*; Secondly, green algae, red algae, diatoms, oomycetes and plants generally have two Oxa homologs, but surprisingly *C. reinhardtii *has none at all. It is noteworthy that one Oxa in green algae and plants is longer than the other (see Additional file 1). Our further analysis indicated that besides the conserved 60 KD IMP domain, the longer one has an additional C-terminal Tetratricopeptide Repeat (TPR) domain that can be predicated by CDART. In *Oryza sativa*, for example, the TPR domain was predicted to be located between residues 361P and 508 V in the longer Oxa. The longer Oxa is definitely predicted to be a mitochondria-located protein by MitoProt II (see Additional file 3).

Two Alb3 homologs were also identified in many other plants, green algae, and, for the first time in diatoms, in addition to *C. reinhardtii *and *A. thaliana *which had been previously studied [10,11,14]. Only one Alb3 homolog could be found in the red alga *Cyanidioschyron merolae *and, interestingly, four in plant *Populus trichocarp*. No Alb3 homolog could be detected in the completely sequenced genome of *Plasmodium falciparum *and in several partially sequenced genomes of oomycetes (*Phytophthora ramorum, Phytophthora sojae *and *Phytophthora infestans*), though the two lineages were considered to possess typical plastids once [15,16].

### Phylogeny of YidC/Oxa/Alb3 family

#### (1) Evolutionary correlation between prokaryotic YidC and eukaryotic Oxa/Alb3

**Figure 1:**
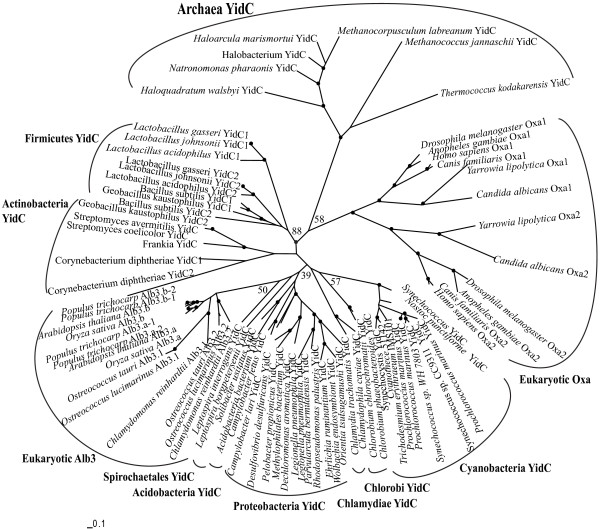
**Maximum-likelihood phylogenetic tree of representative archaea, bacteria and eukaryotes YidC/Oxa/Alb3 protein sequences**. The tree is based on alignment of the full sequences. The nodes with bootstrap support values more than 70% are marked by black dots and the bootstrap support values for some important nodes are shown directly in the tree.

Homologous sequences from representative species of archaea, bacteria and eukaryotes were used to perform phylogenetic analysis. It was showed that mitochondrial Oxa, chloroplastic Alb3, and archaeal YidC form three separate clades, respectively, while bacterial YidC alone form several other clades that are largely consistent with their source organism lineages (Figure 1). The mitochondrial Oxa clade does not group with proteobacterial YidC clade; The Alb3 clade is nested within the large bacterial YidC clades without obviously showing a close relationship with the cyanobacterial YidC clade.

The AU test significantly rejects (*P *< 0.05) that proteobacteria YidC is a sister of Oxa (see Additional file 4). The topologies placing cyanobacteria YidC as sister of Alb3 have low P values (*P *= 0.009, *P *= 0.154) in the tests. Although a possible close relationship between Alb3 and cyanobacterial YidC cannot be significantly rejected, the topology shown in Figure 1 is supported to be the most likely evolutionary scenario (*P *= 0.889) by the AU test, suggesting that Alb3 is unlikely of cyanobacterial origin.

To test whether the high divergence of archaeal YidC leads to the low support values of our Figure 1, further phylogenetic analyses (see Additional file 5) were conducted only including representative bacterial and eukaryotic sequences. These additional analyses generated a similar topology without any obvious increase in support values.

#### (2) Phylogeny of Oxa and Alb3 subfamilies in eukaryotes

**Figure 2:**
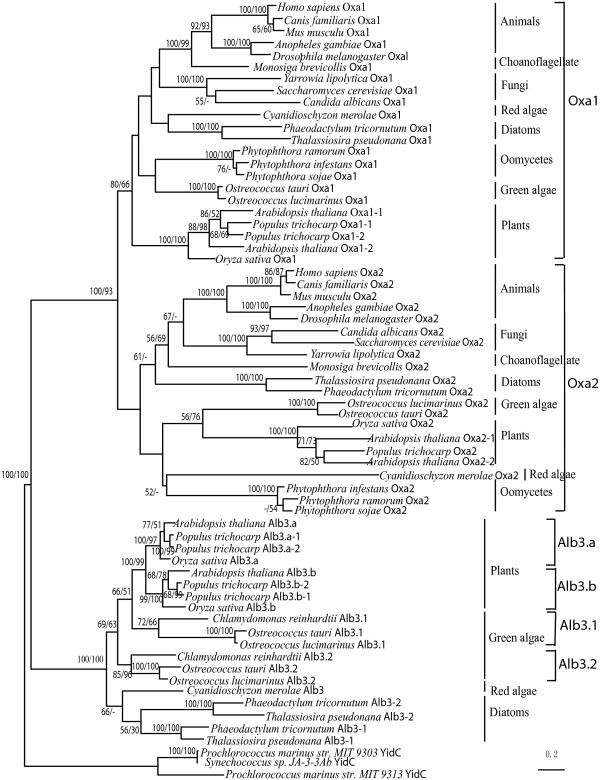
**Phylogeny of Oxa and Alb3 subfamilies in eukaryotes**. Phylogenetic tree of Oxa/Alb3 from representative eukaryotes based on alignment of the full protein sequences with bacterial YidC as outgroup. NJ and ML methods were both used. ML tree was shown with bootstrap values for NJ and ML analyses (the first and second values, respectively), the bootstrap values exceeding 50% were showed.

To investigate the evolutionary history of Oxa and Alb3 in eukaryotes, phylogenetic analyses were performed based on most of the Oxa and Alb3 protein sequences from diverse eukaryotes. The ML and NJ methods gave essentially the same tree topology except for some minor details. Oxa sequences from all eukaryotes form two separate groups (Figure 2) and they are denoted Oxa1 and Oxa2 respectively as suggested before [7]. These data suggest that the Oxa1 and Oxa2 genes are derived from a single gene duplication that occurred at least as ancient as before the divergence of algae, fungi and metazoa.

It is notable that Oxa2 from algae and plants have undergone accelerated evolution which is reflected by their relatively long branches in the phylogenetic tree (Figure 2). Among them, the Oxa2 sequences from green algae and plants all (but except *A. thaliana *Oxa2-2) possess an additional TPR domain as mentioned above, which might partially contribute to their long branches. Such accelerated evolution can also be seen from the matrix of amino acid sequence identities among these sequences (see Additional file 6), which shows that the percent identities among Oxa2 sequences are always lower than those among Oxa1 homologs in green algae and plants.

**Figure 3:**
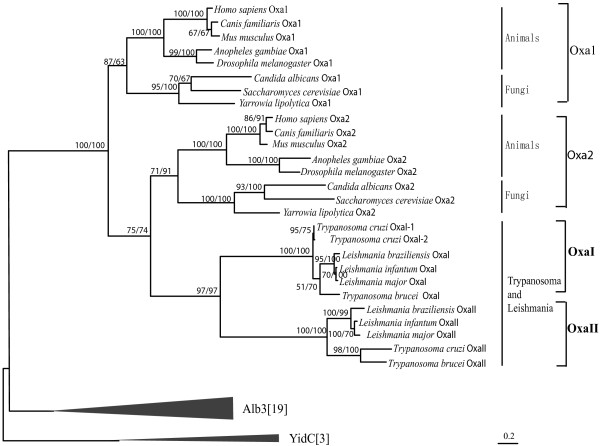
**Phylogeny of Oxa subfamily**. The tree is based on alignment of the full protein sequences with Alb3 and YidC as outgroups. It mainly shows the phylogeny of Trypanosoma and Leishmania Oxa. NJ and ML methods were both used, and percent bootstrap values are given as in Figure 2. Numbers of species for the condensed branches are shown in brackets.

*Trypanosoma *and *Leishmania *homologous genes are usually highly divergent from those of other eukaryotes [17]. This is also true to Oxa. Because such divergence can affect the phylogenetic tree seriously, especially when combined with the fast evolved alga and plant Oxa2 genes, they were excluded from the phylogenetic analyses above. To determine their phylogenetic affinity, further analyses were performed with a smaller data set that included samples from fungal and animal Oxa, YidC and Alb3. It was showed that all *T. cruzi, T. brucei and L. major *Oxa homologs cluster together and form two groups, each including one homolog of the two of each species. Both these groups cluster together with the Oxa2 clade, indicating a recent Oxa2 duplication and the loss of Oxa1 in these kinetoplastid. We here named the two kinetoplastid Oxa groups as OxaI and OxaII instead of Oxa1 and Oxa2 (Figure 3).

Alb3 homologs from green algae and plants cluster together in our phylogenetic trees (Figure 2 and Figure 3). Within this large clade, green alga Alb3 form two distinct but adjacent branches, which are designated as Alb3.1 and Alb3.2 respectively [14], and each branch includes one of the two Alb3 copies. Plant Alb3 also split into two distinct and adjacent groups that are in turn sister to green alga Alb3.1. We named the two plant Alb3 groups Alb3.a and Alb3.b, respectively. Each of the two groups comprise of one Alb3 from *A. thaliana*, *Oryza sativa*, and two from *Populus trichocarp *(the four homologs from *P. trichocarp *were named Alb3.a-1, Alb3.a-2, Alb3.b-1, and Alb3.b-2, respectively). Although bootstrap values are sometimes not particularly strong, additional AU tests significantly rejected the scenario that alga Alb3.1 and Alb3.2 are sister groups (*P *= 0.028), suggesting that the Alb3 gene duplication event happened in the common ancestor of plant and green algae rather than in green algae lineage specifically, and that the secondary loss of one Alb3 gene occurred in the process of evolutionary origin of plant from green algae. However, it is hard for us to determine which Alb3 gene (Alb3.1 or Alb3.2) had been lost in the process as the alternative tree placing alga Alb3.2 as a sister group to plant Alb3 was not be rejected significantly (*P *= 0.415) in our AU test.

Outside the green alga-plant Alb3 clade, the only one Alb3 of red alga (*C. merolae*) forms a separate branch, and the two Alb3 (named Alb3-1, Alb3-2) of diatoms (*P. tricornutum*, *T. pseudonana*) form two other separate branches (Figure 2 and Figure 3).

## Discussion

### YidC in archaea and bacteria

Although nine YidC homologs were reported in archaea by earlier studies [2], only a limited taxonomic sample was covered and the distribution of YidC in the entire archaea domain was largely unclear. Here we have investigated all the available 45 species in all the three archaea lineages, Crenarchaeota, Euryarchaeota, and Nanoarchaeota, but only identified seven YidC homologs, including two old ones (the other reported ones were denied by our reciprocal BLAST search). All the seven homologs were identified in Euryarchaeota. They all contain the conserved homologous transmembrane domain though not the typical 60 KD-IMP (YidC) domain. These results suggest that YidC might have arisen in the Euryarchaeota lineage. However, whether these identified homologs in Euryarchaeota maintain the same function as their bacterial homologs and whether these functions are substituted by other translocases in other archaea remain interesting issues for further studies.

Our survey covers much more bacterial species and lineages (589 species in 8 lineages) than earlier studies. The results confirmed that YidC is ubiquitous in bacteria, with most bacteria possessing one YidC copy and some *Bacillus *and Lactobacillales possessing two copies. In addition, we found that some Actinobacteria and one *Clostridium *also have two YidC copies, and that all the bacteria bearing two YidC copies are Gram-positive bacteria. These results indicate YidC is a common protein translocation system in bacteria. It remains to be further investigated why so many Gram-positive bacteria bear two YidC.

### The diversity and evolution of Oxa in eukaryotes

Our investigation showed a rich diversity of Oxa in eukaryotes, reflecting the specific divergences of the translocases in different lineages.

Our survey showed that besides plants, fungi and metazoans, which were studied previously [7], protists (including green algae, red alga, diatoms, oomycetes and choanoflagellate) also possess two Oxa versions (Oxa1 and Oxa2). This suggests that a very early Oxa duplication must have occurred at least prior to the divergence of protists, fungi, plants and metazoans, that is, in the common ancestor of theses lineages. Thus, our results provide stronger evidence than the previous work, which was only carried out on higher eukaryotes [6,7], for the supposition that the duplication occurred during the early evolution of mitochondria [6,7]. The formation of Oxa1 and Oxa2 may be one of the prerequisites for transforming a bacterial endosymbiont into a mitochondrion during the evolution of eukaryotic cells.

Gene duplication often leads to functional differentiation between homologs. It has been suggested that, by acquiring a ribosome-binding coiled-coil structure in the C-terminal to facilitate the interaction with mitochondrial ribosome, Oxa1 carries out co-translational transport of neo-peptides while Oxa2 carries out a post-translational transport in the biogenesis of cytochrome oxidase [6,18]. However, our data indicate that such a C-terminal coiled-coil structure is only restricted to a few Oxa1 sequences (data not show). In addition, only one substrate of Oxa2 has been found so far, and little is known about the function of Oxa2. Therefore, the above "co- and post-translational transport" hypothesis is doubtful, and actually the functional divergence and collaboration between Oxa1 and Oxa2 is unclear yet. Nevertheless, this issue might be intimately related to the early establishment of proteobacterial endosymbionts or mitochondria during the origin and evolution of eukaryotic cells.

After the early duplication in the common ancestor of eukaryotes, Oxa diverged in different eukaryotic lineages, especially in protists, resulting in rich diversity or adaptive differentiation.

1) Oxa genes in *C. reinhardtii *were completely lost. The reason for such a specific loss is unknown. There may be another system to substitute the Oxa system in this organism.

2) In *Trypanosoma *and *Leishmania*, Oxa sequences form two other groups (OxaI and OxaII) that are sister to Oxa2, rather than being included in Oxa1 and Oxa2 groups of the other organisms. This might reflect an independent duplication occurred in the common ancestor of *Trypanosoma *and *Leishmania*. The most likely scenario for the evolution of OxaI and OxaII includes a loss of Oxa1 and a lineage-specific duplication of Oxa2. These kinetoplastids possess a peculiar mitochondrion called kinetoplastid, which is unique in many respects [19,20]. Therefore, such a condition of Oxa might relate to the peculiar mitochondria in these organisms. It will be interesting to study why OxaI in *T. cruzi *duplicated again and produced twocopies.

3) Oxa in amitochondriate protozoa is completely absent. Our investigation also indicated that the genes of Oxa substrates (such as Cox2, Cox3, F0F1-ATPase [6,21]) and Oxa-associated proteins (including Mba1, PET122, and Rmp1 [22-24]) are also absent in these organisms (data not shown). These amitochondriate protozoa lack canonical mitochondrion but have mitosome or hydrogensome, which contain no respiratory complex and genome [25]. Therefore, the Oxa translocation system may have lost completely in these organisms.

4) Green algae and plants have two Oxa, but their Oxa2 sequences are obviously longer because of an additional C-terminal TPR domain. Since this domain is not present in Oxa of any other lineages, the acquisition of the TPR domain in Oxa2 of these organisms must have occurred after the split of green algae from other eukaryotic lineages. TPR domain has been shown in various organisms to mediate protein-protein interactions and assembly of multiprotein complexes [26]. Proteins containing TPRs are involved in a variety of biological processes, one of which is protein transport [27]. It was reported that many mitochondrial out membrane translocases including Tom20, Tom70 and Tom34 possess TPR domain, [28-30]. No inner membrane translocase was ever found to have this domain so far. Therefore, this is the first report that TPR domain occurs in a mitochondrial inner membrane translocase. Similar differences in mitochondrial translocases between plant and yeast were considered to be involved in avoiding mitochondria mistakenly importing chloroplast proteins in plants [31]. Therefore, the additional TPR domain of Oxa2 in green algae and plants might also to be involved in keeping away similar mistaken import of chloroplast proteins. But the inner-membrane location of Oxa in mitochondria and the absence of TPR domain in Oxa of red alga and diatoms weaken this hypothesis.

### Alb3 in plastid-containing organisms and its evolution

Except for *C. merolae*, our survey indicated that almost all plastid-containing eukaryotes investigated possess at least two Alb3 copies. Our phylogenetic analyses indicated that Alb3 gene duplication and loss occurred several times during the evolution of plastid-containing eukaryotes. The first duplication event might occurred at least in the last common ancestor of green algae and produced Alb3.1 and Alb3.2; Then, during the emergence of land plant from green algae, one Alb3 gene copy was lost and the other was subject to a second duplication event and led to the two genes (Alb3.a and Alb3.b) found in present plants; Interestingly, the two plant Alb3 genes both duplicated recently once again in *P. trichocarp *and produced four Alb3 genes. Additionally, another duplication of Alb3 occurred in the ancestor of diatoms and resulted in two Alb3 (Alb3-1 and Alb3-2) in extant diatoms. The red alga *C. merolae *possesses only one Alb3 copy, and this may be related to its smallest genome among all photosynthetic eukaryotes [32]. An Alb3 gene copy might have been lost in *Cyanidioschyzon *in the process of its genome reduction.

Oomycetes are often considered to have secondarily lost their plastids and become nonphotosynthetic [16,33]. No Alb3 could be identified in oomycetes, suggesting a loss of the Alb3 system likely as a result of loss of plastids. *Plasmodia *possess degenerated plastids, but no Alb3 gene and its substrate (e.g. Lhcb4.1 and Lhcb5) genes could be detected (data not show). This suggests that Alb3 pathway exists no longer in the degenerated plastids.

The universal distribution of two Alb3 in plastid-containing organisms may imply distinct functions for the two Alb3 copies. According to Woolhead et al [8] and Gohre et al [11], Alb3.1/a is responsible for the assembly of photosystem units into thylakoid membrane, whereas Alb3.2/b is in charge of the formation of proper chloroplast ultrastructure and also partially involved in photosynthesis. Photosynthesis and chloroplast biogenesis are two important activities for photosynthetic eukaryotes. The availability of two Alb3 gene copies will render the regulation of the two separate activities more effectively. Anyway, to determine the functional differentiation between Alb3.1 and Alb3.2, Alb3.a and Alb3.b would gather more attention in the future.

### The origin of eukaryotic Oxa and Alb3: not directly from the YidC of the bacterial endosymbiotic ancestors of mitochondria and chloroplasts

YidC/Oxa/Alb3 family was customarily considered as a good example to exhibit the conservation of protein transporting system in prokaryotes and eukaryotic endosymbionts [1,34]. According to the endosymbiosis theory, it seems reasonable to suppose that Oxa and Alb3 are derived directly from the YidC of the bacterial progenitors of mitochondrion and chloroplast, respectively [3] Consequently, like many mitochondrial or chloroplastic proteins [35,36], Oxa and Alb3 are expected to group with YidC sequences from proteobacteria and cyanobacteria respectively. However, our phylogenetic analyses indicated that, although Oxa and Alb3 do have two separate origins, they are not particularly related to proteobacterial and cyanobacterial YidC sequences, respectively. Instead, Oxa sequences appear to form a separate clade; Alb3 sequences group with YidC clades of various bacterial lineages with generally poor bootstrap support and their specific affiliation cannot be decisively pinpointed from our data. Therefore, our studies have denied the supposition that Oxa and Alb3 originated directly from YidC of the bacterial progenitors of the two organelles. What prokaryotic YidC once gave rise to the eukaryotic Oxa and Alb3 remains an issue for further study.

## Conclusion

**Figure 4:**
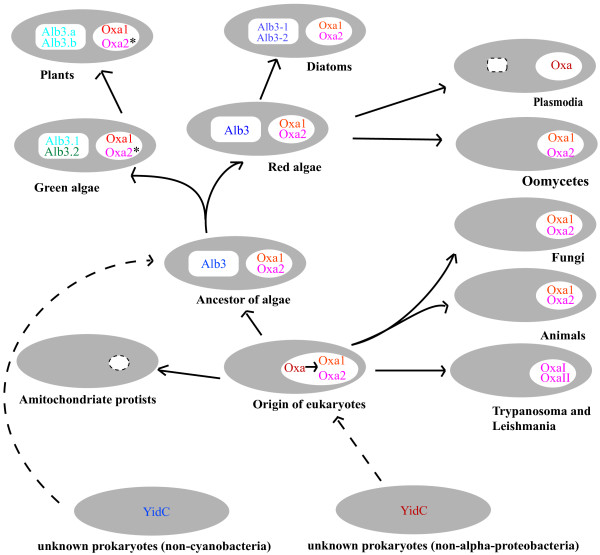
**An evolutionary route chart of the YidC/Oxa/Alb3 family in the three domains of life (for detailed illustration please see the text)**. In cells, chloroplast is represented by polygon and mitochondrion by ellipse. Degenerated mitochondrion and plastid are represented by dashed polygon and dashed ellipse, respectively. Asterisks (*) indicate the Oxa2 possessing an additional C-terminal TPR domain in green algae and plants. Dashed line indicates an uncertain relationship. The color of the letters refers to their evolutionary correlations of the members of the family.

We propose a scenario of the evolutionary history of the YidC/Oxa/Alb3 family (Figure 4) mainly based on the results generated from this study. In this model, YidC gene arose in some Euryarchaea and all Bacteria, and later in some gram-positive bacteria the gene duplicated to produce two copies. The eukaryotic Oxa, Alb3 have two separate prokaryotic origins, which might not be directly related to the endosymbiotic origins of mitochondria and chloroplasts. An early Oxa duplication in the common ancestor of eukaryotes led to Oxa1 and Oxa2, and thus most eukaryotes generally bear two Oxa. However, secondary loss, duplication or acquisition of new domain also occurred on Oxa genes in some lineages, especially in protists, resulting in a rich diversity or adaptive differentiation of the translocase in these lineages. A subsequent Alb3 duplication led to the origin of Alb3.1 and Alb3.2 in green algae, and one of them was lost and the other underwent another duplication and generated two other Alb3 copies (Alb3.a and Alb3.b) in the evolution of land plants. In diatoms, another duplication of Alb3 occurred and produced Alb3-1 and Alb3-2. Alb3 genes were also lost partially (e.g. in red alga *C. merolae*) or completely (e.g. in *Plasmodium *and *Oomycetes*) in eukaryotes that either lost the plastids entirely or only retain a relic plastid. This model outlines the evolutionary history of the Oxa/Alb3/YidC family in the three domains of life.

## Methods

### Search and identification of YidC/Oxa/Alb3 homologs

Protein sequence of *Escherichia coli *YidC (NP_756486) was used as query to search against all available completed (589, up to October 2008) bacterial genomic databases with protein annotation in GenBank with E value 1e-03 as cutoff to get candidate homologs; Using the protein sequences of *Homo sapiens *Oxa1 and Cox18 (NP_005006; NP_776188) and *C. reinhardtii *Alb3.1 and Alb3.2 (AAM11662; AAM49792) as queries, eukaryotic candidate homologs were retrieved by BLASTP and TBLASTN searches (E-value < 10^-3^) from 51 eukaryotic genome databases in NCBI , Doe Joint Genome Institute , the Institute For Genome Research  and Broad institute . Then, the obtained candidate homologs were retained as putative ones only after 1) they were predicted to have the conserved 60 KD-IMP or YidC domain by CDART (Conserved Domain Architecture Retrieval Tool) [37] with default parameters; 2) their best hits of BLASTP against NCBI nr database were known YidC/Oxa/Alb3. To acquire archaeal homologs, all sequences obtained hereinbefore were used as queries to PSI-blast search against 45 annotated archaeal genome databases for five iterations, with E value 1e-03 as cutoff, and then they were accepted as putative homologs only if their best hits of BLASTP against nr database were known YidC/Oxa/Alb3.

In order to search exhaustively the homologs from protozoa and algae, which are usually highly divergent from those of other lineages, the above obtained putative homologs from these lineages were also used as queries to search back against all the available databases of these lineages themselves to find more, if any, candidate homologs, and as above, the conserved 60 KD-IMP or YidC domain and the best hit were two criteria to determine whether are putative homologs.

At last, the assignment of the above obtained putative eukaryotic homologs to Oxa or Alb3 is according to the highest expect values produced by *H. sapiens *Oxa or *C. reinhardtii *Alb3 sequences, the best hit sequences of blast search against the nr database, the similarities showed by multiple sequence alignments (Additional file 7), altogether.

MitoProt II  was used as described before [38] for the cellular organelle localization prediction of some Oxa homologs which have particular domain structure.

Tmpred  was used to predict the hydropathy plots of representative members of the different branches of YidC subfamily.

All the homologous sequences (see Additional file 1) obtained from archaea, bacteria and eukaryotes were retrieved from public databases in October, 2008.

### Sequence Alignment and Phylogenetic analysis

Sequence alignment was performed using M-Coffee [39], with default parameters for gap opening and extension, and blosum30mt as the protein weight matrix. After manually refined, the alignments were used to carry out maximum likelihood (ML) and neighbor-joining (NJ) phylogenetic analyses, using PHYML [40] and PHYLIP 3.65 (Felsenstein 2005), respectively.

The ProtTest program [41] was used to select the model of protein evolution that best fits our datasets. The invoked options in the maximum likelihood analysis with PHYML program were 100 bootstrap replications, the RtREV substitution matrix [41], and the gamma distribution model (1 invariable site+8 gamma rate categories) for estimation of rate heterogeneity.

Programs from the PHYLIP package were used to create pseudoreplicate data sets (SEQBOOT), calculate distance trees (NEIGHBOR), and assemble a bootstrap consensus tree (CONSENSE), followed by 1000 replications of bootstrap resampling.

### Tree Topology Tests

The AU nonparametric bootstrap test was used to compare alternative phylogenetic hypotheses. Using the ML tree (Figure 1) as the backbone tree, alternative trees were produced by switching branches using Treeview (version 1.6.6). Site-wise log-likelihoods were calculated for each topology in Tree-Puzzle 5.2 [42], and then were supplied to the program CONSEL (version 0.1j) [43].

## Authors' contributions

YJZ conceived the project, carried out the sequence alignments, and performed phylogenetic analysis, HFT performed database searches and JFW supervised the work. YJZ, HFT and JFW wrote the manuscript. All authors read and approved the final manuscript.

## Supplementary Material

Additional file 1 **Identified homologs of YidC/Oxa/Alb3 family from the three domains of life**. Homologs were retrieved from archaea, bacteria and eukaryotes, only those species used in our phylogenetic analyses are listed in detail. Click here for file

Additional file 2 **Hydropathy plots for representative members of the different branches of YidC subfamily**. Hydropathy plots were obtained by using Tmpred to predict with default parameter settings. X-axis represents position of amino acids. Y-axis represents hydropathy values. Click here for file

Additional file 3 **Targeting prediction for green algae and plants Oxa2 proteins with particular TPR domain**. The subcellular locations were predicted by MitoProt II. Click here for file

Additional file 4 **Comparison of alternative tree topology regarding the endosymbiosis origin of Oxa, Alb3 and the secondary loss of Alb3 in plants**. The approximately unbiased (AU) test, bootstrap probability (BP), and unweighted and weighted (W) Kishino-Hasegawa (KH) and Shimodaira-Hasegawa (SH) tests were used, all calculated using CONSEL. Click here for file

Additional file 5 **Maximum-likelihood phylogenetic tree of representative bacteria and eukaryotes YidC/Oxa/Alb3 protein sequences**. The tree is based on alignment of the full sequences. The nodes with bootstrap support values more than 50% were indicated. Click here for file

Additional file 6 **Protein identities (%) between mitochondria Oxa1 and Oxa2 sequences from plants and green algae**. The sequence identities of Oxa1 are in bold-face, and the sequence identities of the Oxa2 are in italic. Click here for file

Additional file 7 **Multiple sequence alignments used in this study**. Multiple sequence alignments used for Figure 1, Figure 2, Figure 3 and Additional file 5 were shown. Click here for file
