# Supplementary material for: The evolution of YidC/Oxa/Alb3 family in the three domains of life: a phylogenomic analysis
Source: BMC Evol Biol. 2009 Jun 18;9:137. doi: 10.1186/1471-2148-9-137 (PMC2706819; doi:10.1186/1471-2148-9-137)
Supplement: Additional file 1 — Identified homologs of YidC/Oxa/Alb3 family from the three domains of life. Homologs were retrieved from archaea, bacteria and eukaryotes, only those species used in our phylogenetic analyses are listed in detail. [file 1471-2148-9-137-S1.doc]

**Additional file 1**

**1. Archaea**

| **Lineage** | **Species** | **Homolog** | **Accession** | **Size**  **(aa)** | **Notes** |
| --- | --- | --- | --- | --- | --- |
| Eury  archaeota | *Haloquadratum walsbyi DSM 16790*  *Natronomonas pharaonis DSM 2160*  *Halobacterium sp. NRC-1*  *Haloarcula marismortui ATCC*  *43049*  *Methanocorpusculum labreanum Z*  *Methanococcus jannaschii*  *Thermococcus kodakarensis KOD1* | YidC  YidC  YidC  YidC  YidC  YidC  YidC | YP_658478.1  YP_331181.1  NP_280484.1  YP_137740.1  YP_001029550.1  NP_247456.1  YP_183929.1 | 308  298  290  296  212  198  174 | Domain: COG1422  Domain: COG1422  Domain: COG1422  Domain: COG1422  Domain: COG1422  Domain: DUF106  Domain: COG1422 |
|  | another 24 species | no homolog identified | | | |
| Cren  archaeota | 13 species | no homolog identified | | | |
| Nano  archaeota | 1 species | no homolog identified | | | |

**2. Bacteria**

| **Lineage** | **Species** | | **Homolog** | **Accession** | **Size**  **(aa)** | **Notes** |
| --- | --- | --- | --- | --- | --- | --- |
| Actino  bacteria | *Frankia sp. EAN1pec*  *Streptomyces avermitilis MA-4680*  *Streptomyces coelicolor A3(2)*  *Corynebacterium diphtheriae NCTC 13129* | | YidC  YidC  YidC  YidC1  YidC2 | YP_001511563.1  NP_825489.1  NP_628069.1  NP_940679.1  NP_940576.1 | 461  428  431  317  435 |  |
|  | another 40 species | | 47 homologs identified | | | |
| Bacteroidetes  /Chlorobi | *Chlorobium chlorochromatii CaD3*  *Chlorobium phaeobacteroides BS1* | | YidC  YidC | YP_380321.1  ZP_00530951.1 | 584  587 |  |
|  | another 38 species | | 38 homologs identified | | | |
| Cyano  bacteria | *Prochlorococcus marinus str. MIT 9303*  *Synechococcus sp. JA-3-3Ab*  *Prochlorococcus marinus str. MIT 9313*  *Synechocystis sp. PCC 6803*  *Cyanothece sp. ATCC 51142*  *Trichodesmium erythraeum IMS101*  *Nostoc punctiforme PCC 73102*  *Synechococcus sp. WH 7803*  *Synechococcus sp. CC9311*  *Prochlorococcus marinus str. MIT 9312* | | YidC  YidC  YidC  YidC  YidC  YidC  YidC  YidC  YidC  YidC | YP_001016626.1  YP_475202.1  NP_895198.1  NP_441564.1  YP_001802780.1  YP_724146.1  YP_001865213.1  YP_001225624.1  YP_731297.1  YP_397782.1 | 377  392  378  384  378  382  383  383  382  380 |  |
|  | another 36 species | | 36 homologs identified | | | |
| Chlamydiae | *Chlamydia trachomatis A/HAR-13*  *Chlamydophila caviae GPIC* | | YidC  YidC | YP_328059.1  NP_829339.1 | 787  794 |  |
|  | another 9 species | | 9 homologs identified | | | |
| Firmicutes | Bacillales | *Bacillus subtilis subsp. subtilis str. 168*  *Geobacillus kaustophilus HTA426* | YidC1  YidC2  YidC1  YidC2 | NP_391984.1  NP_390269.1  YP_149349.1  YP_147675.1 | 261  275  254  249 |  |
|  |  | another 37 species | 41 homologs identified | | | |
|  | Clostridia | 25 species | 26 homologs identified | | | |
|  | Lactobacillales | *Lactobacillus gasseri ATCC 33323*  *Lactobacillus johnsonii NCC 533*  *Lactobacillus acidophilus NCFM* | YidC1  YidC2  YidC1  YidC2  YidC1  YidC2 | YP_819589.1  YP_815196.1  NP_965834.1  NP_965435.1  YP_194373.1  YP_194801.1 | 291  327  291  327  322  291 |  |
|  |  | another 42 species | 64 homologs identified | | | |
| Acido  bacteria | *Solibacter usitatus Ellin6076*  *Acidobacteria bacterium Ellin345* | | YidC  YidC | YP_822371.1  YP_589296.1 | 579  583 |  |
| Others | 18 species | | 18 homologs identified | | | |
| Proteobacteria | Others | One species | 1 homolog identified | | | |
|  | alpha subdivision | *Parvularcula bermudensis HTCC2503*  *Rhodopseudomonas palustris BisA53*  *Ehrlichia ruminantium str. Welgevonden*  *Wolbachia endosymbiont strain TRS of Brugia malayi*  *Orientia tsutsugamushi str. Ikeda* | YidC  YidC  YidC  YidC  YidC | ZP_01018491.1  ZP_00811788.1  YP_197195.1  YP_198021.1  YP_001937521.1 | 589  626  575  572  567 |  |
|  | beta subdivision | *Methylophilales bacterium HTCC2181*  *Dechloromonas aromatica RCB* | YidC  YidC | ZP_01551557.1  YP_287397.1 | 546  547 |  |
|  |  | another 43 species | 43 homologs identified | | | |
|  | delta subdivision | *Pelobacter propionicus*  *DSM 2379*  *Desulfovibrio desulfuricans subsp. desulfuricans str. G20* | YidC  YidC | YP_903272.1  YP_388886.1 | 542  536 |  |
|  |  | another 14 species | 14 homologs identified | | | |
|  | epsilon subdivision | *Campylobacter fetus subsp. fetus 82-40*  *Campylobacter lari RM2100* | YidC  YidC | YP_891743.1  ZP_00368969.1 | 531  526 |  |
|  |  | another 17 species | 17 homologs identified | | | |
|  | gamma subdivision | *Legionella pneumophila subsp. pneumophila str. Philadelphia 1*  *Xanthomonas campestris pv. campestris str. 8004* | YidC  YidC | YP_096994.1  YP_245385.1 | 556  573 |  |
|  |  | another 140 species | 140 homologs identified | | | |
| Spiro  chaetales | *Leptospira borgpetersenii*  *serovar Hardjo-bovis L550*  *Leptospira interrogans serovar Lai str. 56601* | | YidC  YidC | YP_799186.1  NP_710359.1 | 622  627 |  |
|  | another 7 species | | 7 homologs identified | | | |

**3. Eukarytes**

| **Lineage** | **Species** | **Homolog** | **Accession** | **Size**  **(aa)** | **Notes** |
| --- | --- | --- | --- | --- | --- |
| Protist | *Monosiga brevicollis* | Oxa1 | 1400 | 235 | Protein IDs from JGI |
|  |  | Oxa2 | 32371 | 384 |  |
|  | *Trypanosoma brucei TREU927* | OxaI | XP_828689.1 | 418 |  |
|  |  | OxaII | XP_827302.1 | 424 |  |
|  | *Trypanosoma cruzi* | OxaI-1 | EAN85125 | 428 |  |
|  |  | OxaI-2 | EAN98364 | 428 |  |
|  |  | OxaII-1 | [XP_810400.1\|](http://www.ncbi.nlm.nih.gov/entrez/query.fcgi?cmd=Retrieve&db=Protein&list_uids=71416854&dopt=GenPept) | 434 |  |
|  | *Leishmania major strain Friedlin* | OxaI | XP_001681584.1 | 407 |  |
|  |  | OxaII | AAK38135 .1 | 532 |  |
|  | *Leishmania infantum JPCM5* | OxaI | XP_001463916.1 | 441 |  |
|  |  | OxaII | XP_001469267.1 | 558 |  |
|  | *Leishmania braziliensis MHOM/BR/75/M2904* | OxaI | XP_001563031.1 | 381 |  |
|  |  | OxaII | XP_001568555.1 | 560 |  |
|  | *Plasmodium falciparum* | Oxa | NP_704272 | 589 | # |
|  | *Chlamydomonas reinhardtii* | Alb3.1 | AAM11662 | 495 |  |
|  |  | Alb3.2 | AAM49792 | 422 |  |
|  | *Ostreococcus tauri* | Oxa1 | 33312 | 337 | Protein IDs from JGI |
|  |  | Oxa2 | 34956 | 430 |  |
|  |  | Alb3.1 | 3067 | 259 | uncompleted CDS |
|  |  | Alb3.2 | 15979 | 399 |  |
|  | Ostreococcus lucimarinus | Oxa1 | 40894 | 304 | Protein IDs from JGI |
|  |  | Oxa2 | 93298 | 427 |  |
|  |  | Alb3.1 | 3329 | 264 | uncompleted CDS |
|  |  | Alb3.2 | 3334 | 271 | uncompleted CDS |
|  | *Cyanidioschyzon merolae* | Oxa1 | CMC118C | 426 | Protein IDs from JGI |
|  |  | Oxa2 | CME168C | 369 |  |
|  |  | Alb3 | CMT063C | 488 |  |
|  | *Phaeodactylum tricornutum* | Oxa1 | 43657 | 422 | Protein IDs from JGI |
|  |  | Oxa2 | 42866 | 664 |  |
|  |  | Alb3-1 | 43657 | 425 |  |
|  |  | Alb3-2 | 46411 | 467 |  |
|  | *Thalassiosira pseudonana* | Oxa1 | 5214 | 370 | Protein IDs from JGI |
|  |  | Oxa2 | 8546 | 426 |  |
|  |  | Alb3-1 | 268324 | 407 |  |
|  |  | Alb3-2 | 34551 | 268 |  |
|  | *Phytophthora ramorum* | Oxa1 | 73907 | 374 | Protein IDs from JGI |
|  |  | Oxa2 | 83927 | 366 |  |
|  | *Phytophthora sojae* | Oxa1 | 127695 | 355 | Protein IDs from JGI |
|  |  | Oxa2 | 137102 | 362 |  |
|  | *Phytophthora infestans* | Oxa1 | PITG_01748 | 371 | * |
|  |  | Oxa2 | PITT_13344 | 360 |  |
| Plant | *Arabidopsis thaliana* | Oxa1-1 | Q42191 | 429 |  |
|  |  | Oxa1-2 | NP_182170 | 431 |  |
|  |  | Oxa2-1 | NP_176688 | 525 |  |
|  |  | Oxa2-2 | NP_190023 | 338 |  |
|  |  | Alb3.1a | NP_180446 | 462 |  |
|  |  | Alb3.1b | CAJ45566 | 499 |  |
|  | *Oryza sativa* | Oxa1 | NP_922381 | 487 |  |
|  |  | Oxa2 | XP_493955 | 528 |  |
|  |  | Alb3.1a | XP_550167 | 459 |  |
|  |  | Alb3.1b | XP_470390 | 489 |  |
|  | *Populus trichocarp* | Oxa1-1 | 259857 | 310 | uncompleted CDS |
|  |  | Oxa1-2 | 751211 | 459 | Protein IDs from JGI |
|  |  | Oxa2 | 557194 | 499 |  |
|  |  | Alb3.1a-1 | 706185 | 451 |  |
|  |  | Alb3.1a-2 | 821561 | 446 |  |
|  |  | Alb3.1b-1 | 419194 | 281 | uncompleted CDS |
|  |  | Alb3.1b-2 | 230358 | 292 | uncompleted CDS |
| Fungi | *Saccharomyces cerevisiae* | Oxa1 | AAT93151 | 402 |  |
|  |  | Oxa2 | NP_011576 | 316 |  |
|  | *Yarrowia lipolytica* | Oxa1 | XP_506118 | 388 |  |
|  |  | Oxa2 | XP_505371 | 314 |  |
|  | *Candida albicans SC5314* | Oxa1 | EAL02067 | 374 |  |
|  |  | Oxa2 | EAL03076 | 331 |  |
|  | another 6 species | 13 homologs identified | | | *Schizosaccharomyces pombe* have 3 |
| Animal | *Homo sapiens* | Oxa1 | NP_005006 | 495 |  |
|  |  | Oxa2 | NP_776188 | 333 |  |
|  | *Canis familiaris* | Oxa1 | XP_537362 | 435 |  |
|  |  | Oxa2 | XP_539312 | 333 |  |
|  | *Mus musculus* | Oxa1 | NP_081212 | 433 |  |
|  |  | Oxa2 | NP_001028482 | 331 |  |
|  | *Drosophila melanogaster* | Oxa1 | NP_648417 | 441 |  |
|  |  | Oxa2 | NP_648286 | 351 |  |
|  | *Anopheles gambiae* | Oxa1 | EAA11925 | 419 |  |
|  |  | Oxa2 | EAA11323 | 285 |  |
|  | another 4 species | 8 homologs identified | | | |

# This sequence was not used for phylogenetic analyses.

* Transcript IDs retrieved from *Phytophthora infestans* genome database in BROAD institute (<http://www.broad.mit.edu/annotation/genome/phytophthora_infestans/Home.html>).
