# Supplementary material for: The evolution of YidC/Oxa/Alb3 family in the three domains of life: a phylogenomic analysis
Source: BMC Evol Biol. 2009 Jun 18;9:137. doi: 10.1186/1471-2148-9-137 (PMC2706819; doi:10.1186/1471-2148-9-137)
Supplement: Additional file 2 — Hydropathy plots for representative members of the different branches of YidC subfamily. Hydropathy plots were obtained by using Tmpred to predict with default parameter settings. X-axis represents position of amino acids. Y-axis represents hydropathy values. [file 1471-2148-9-137-S2.doc]

**Additional file 2**

**1. Archaea**

(1) *Haloquadratum walsbyi DSM 16790* YidC

[
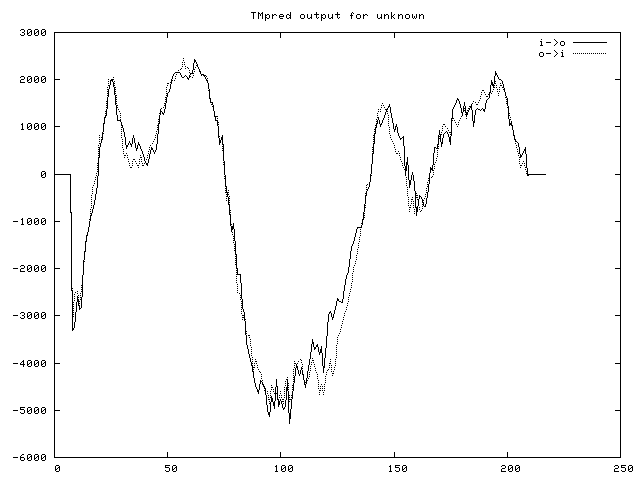
](http://www.ch.embnet.org/wwwtmp/.TMPRED.2739.1572.gif)(2) *Natronomonas pharaonis DSM 2160* YidC

[
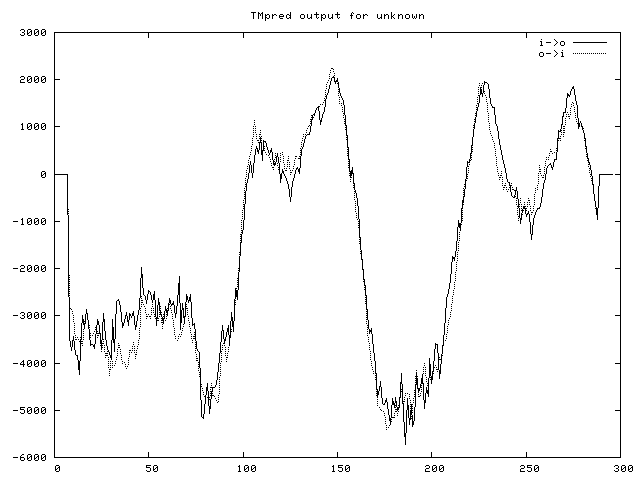
](http://www.ch.embnet.org/wwwtmp/.TMPRED.1689.1106.gif)(3) *Halobacterium sp. NRC-1* YidC

[
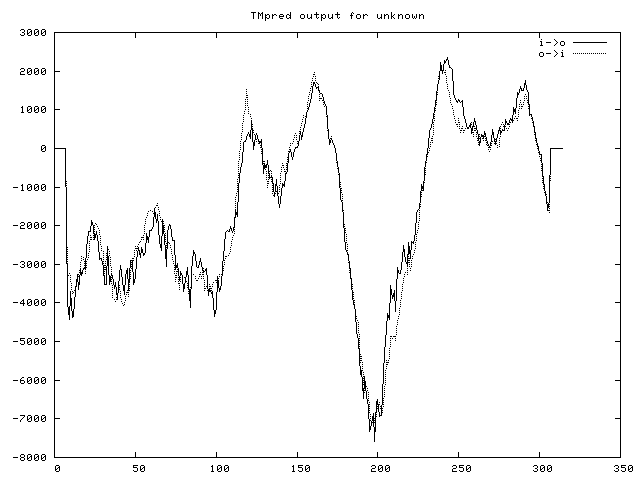
](http://www.ch.embnet.org/wwwtmp/.TMPRED.1987.6224.gif)

(4) *Haloarcula marismortui ATCC 43049* YidC

[
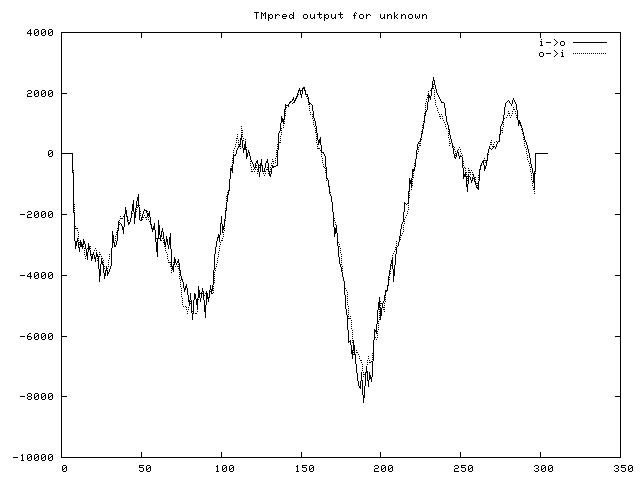
](http://www.ch.embnet.org/wwwtmp/.TMPRED.2150.435.gif)

(5) *Methanocorpusculum labreanum Z* YidC

[
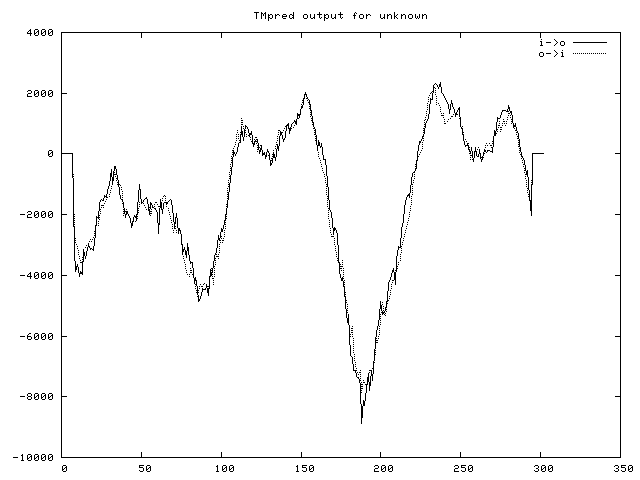
](http://www.ch.embnet.org/wwwtmp/.TMPRED.2740.9092.gif)

**2. Bacteria**

**(1) Actinobacteria**

*Corynebacterium diphtheriae* YidC1

[
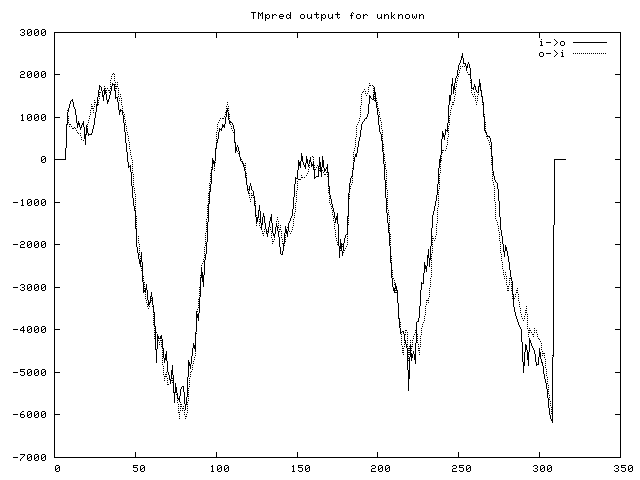
](http://www.ch.embnet.org/wwwtmp/.TMPRED.1688.5810.gif)*Corynebacterium diphtheriae* YidC2

[
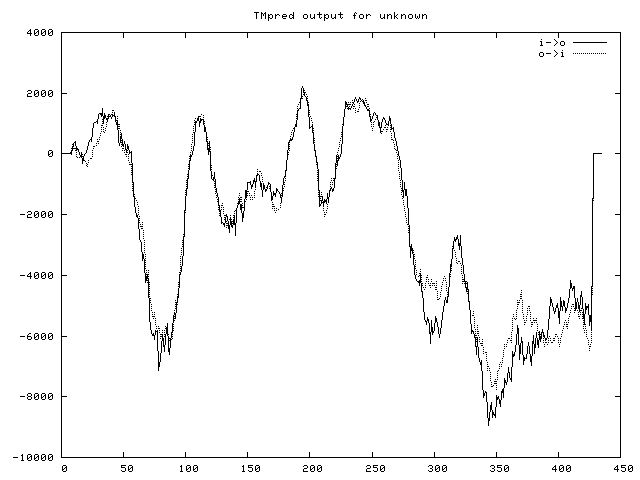
](http://www.ch.embnet.org/wwwtmp/.TMPRED.1684.5623.gif)**(2) Bacteroidetes**

*Chlorobium chlorochromatii CaD3* YidC

[
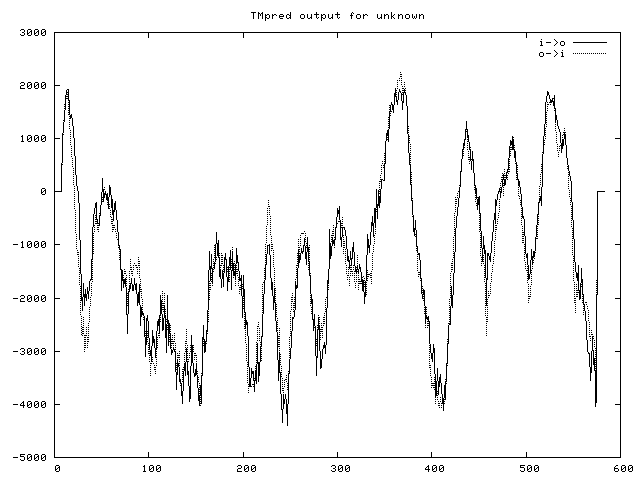
](http://www.ch.embnet.org/wwwtmp/.TMPRED.407.6386.gif)*Chlorobium phaeobacteroides BS1* YidC

[
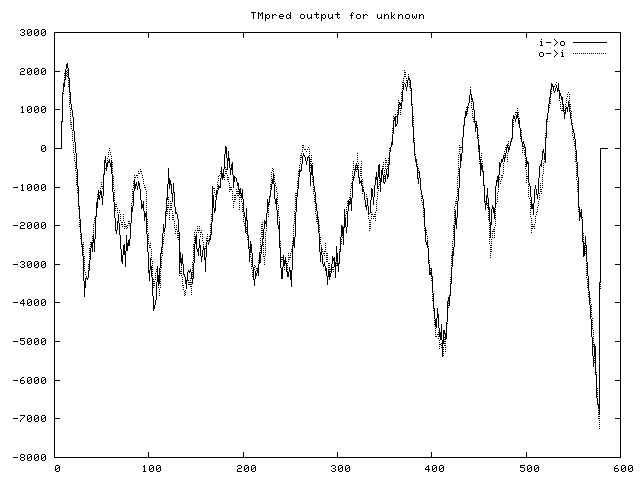
](http://www.ch.embnet.org/wwwtmp/.TMPRED.994.1167.gif)**(3) Chlamydiae**

*Chlamydia trachomatis A/HAR-13* YidC

[
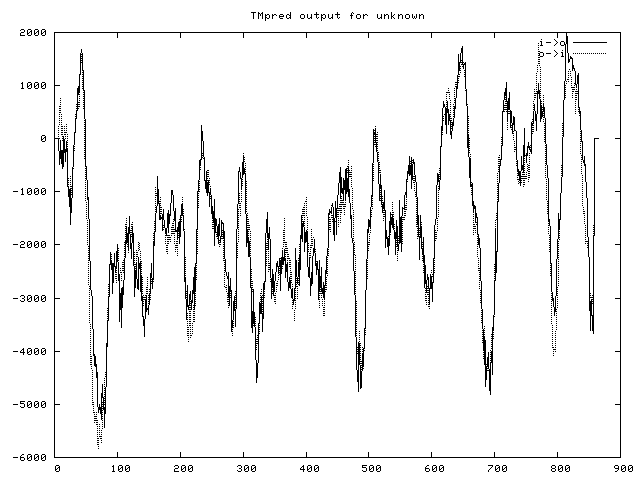
](http://www.ch.embnet.org/wwwtmp/.TMPRED.405.4212.gif)

*Chlamydophila caviae GPIC* YidC

[
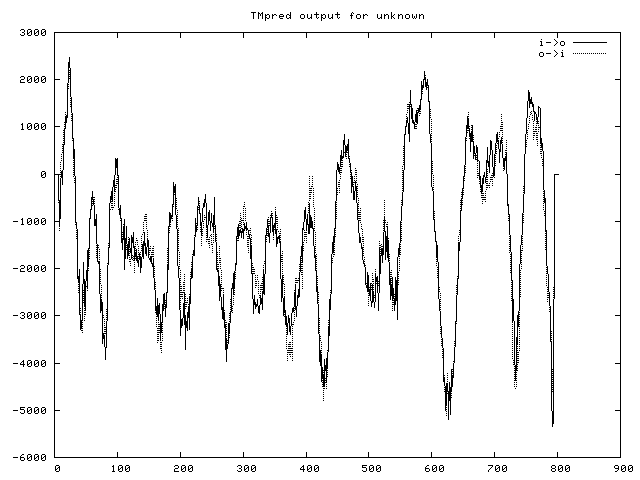
](http://www.ch.embnet.org/wwwtmp/.TMPRED.726.6796.gif)

**(4) Cyanobacteria**

*Prochlorococcus marinus str. MIT 9303* YidC

[
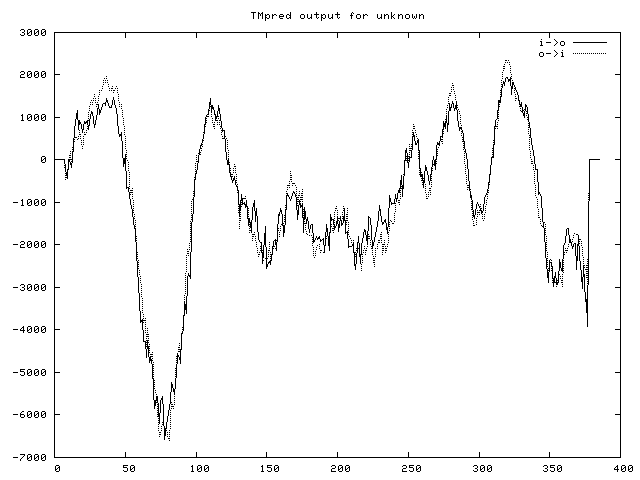
](http://www.ch.embnet.org/wwwtmp/.TMPRED.405.131.gif)

*Prochlorococcus marinus str. MIT 9313* YidC

[
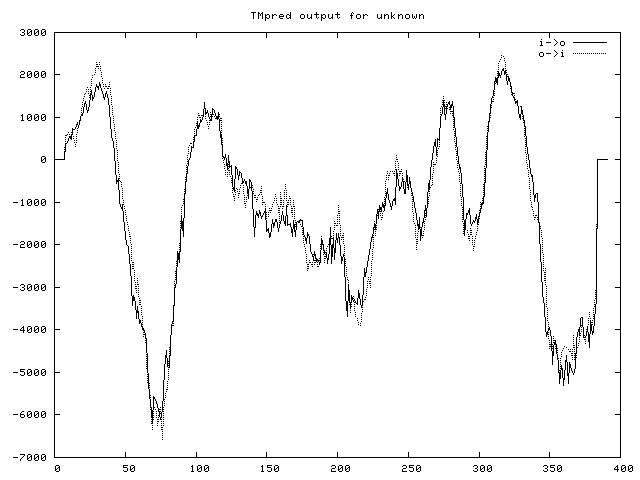
](http://www.ch.embnet.org/wwwtmp/.TMPRED.403.4604.gif) *Synechococcus sp. JA-3-3Ab* YidC

[
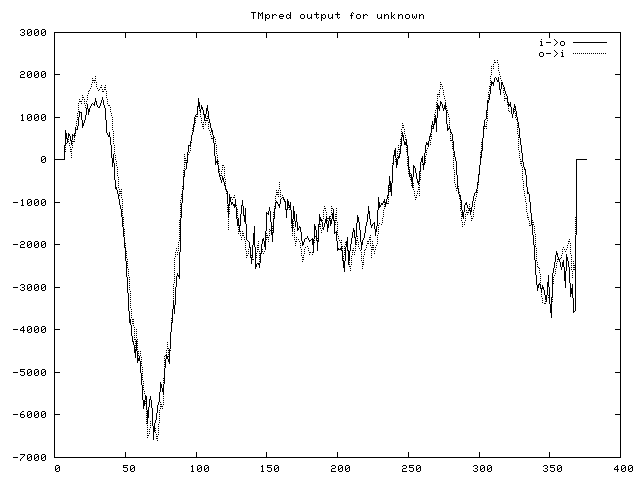
](http://www.ch.embnet.org/wwwtmp/.TMPRED.994.5845.gif)**(5) Firmicutes**

**(5.1) Lactobacillales**

*Lactobacillus gasseri* YidC1

[
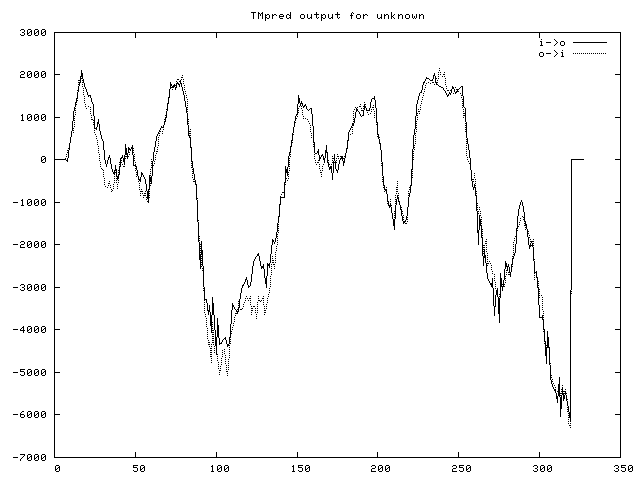
](http://www.ch.embnet.org/wwwtmp/.TMPRED.403.179.gif)*Lactobacillus gasseri* YidC2

[
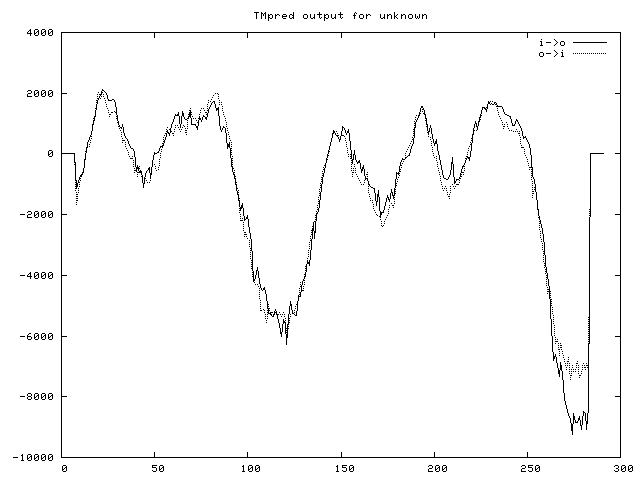
](http://www.ch.embnet.org/wwwtmp/.TMPRED.403.4960.gif)**(5.2) Mollicutes**

*Mycoplasma synoviae* YidC

[
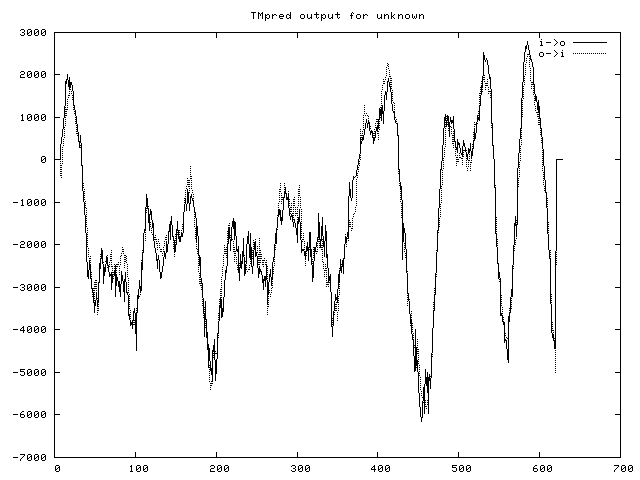
](http://www.ch.embnet.org/wwwtmp/.TMPRED.405.4163.gif)*Mycoplasma agalactiae* YidC

[
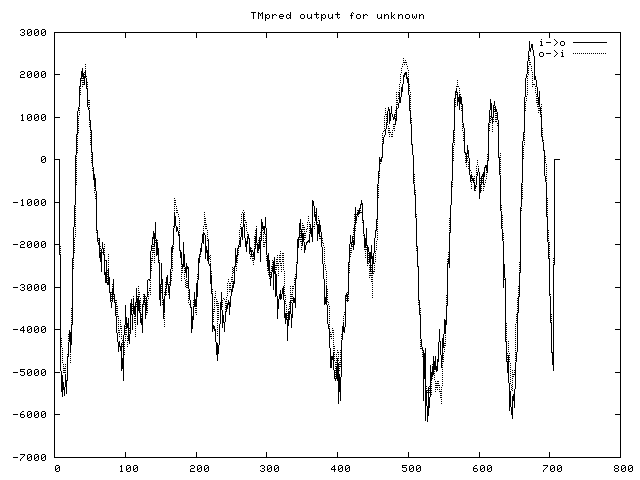
](http://www.ch.embnet.org/wwwtmp/.TMPRED.1019.8505.gif)**(6) Acidobacteria**

*Solibacter usitatus* YidC

[
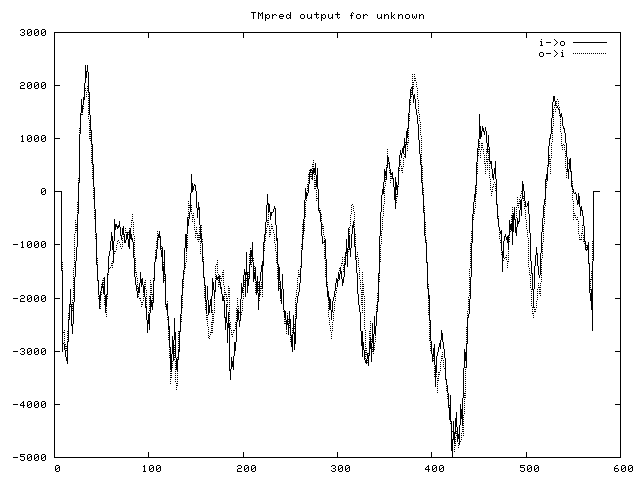
](http://www.ch.embnet.org/wwwtmp/.TMPRED.726.3416.gif)

*Acidobacteria bacterium* YidC

[
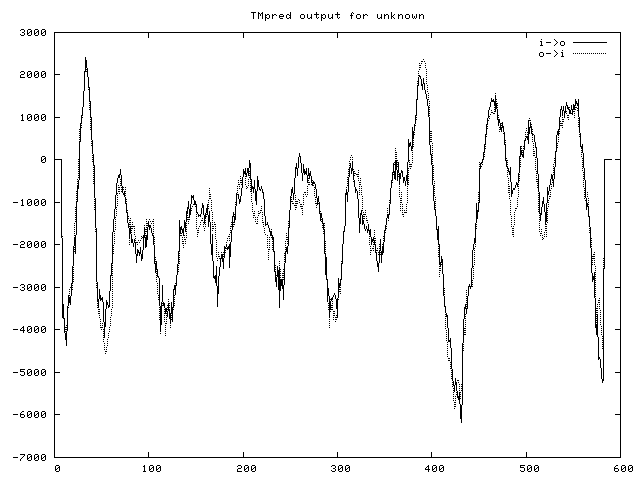
](http://www.ch.embnet.org/wwwtmp/.TMPRED.782.2460.gif)

**(7) Proteobacteria**

**(7.1) alpha subdivision**

*Parvularcula bermudensis* YidC

[
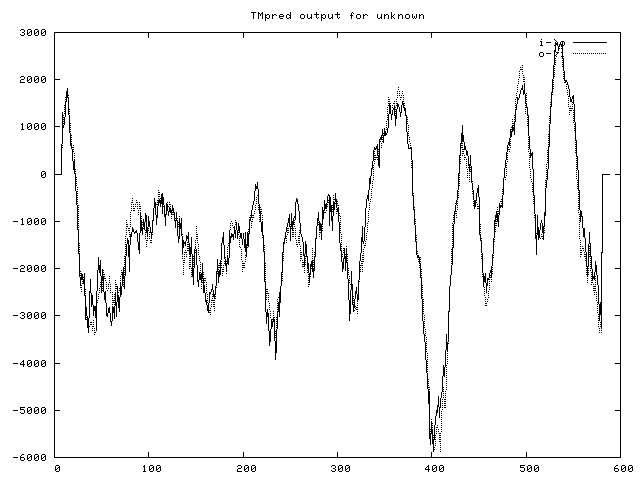
](http://www.ch.embnet.org/wwwtmp/.TMPRED.404.3878.gif)**(7.2) beta subdivision**

*Methylophilales bacterium* YidC

[
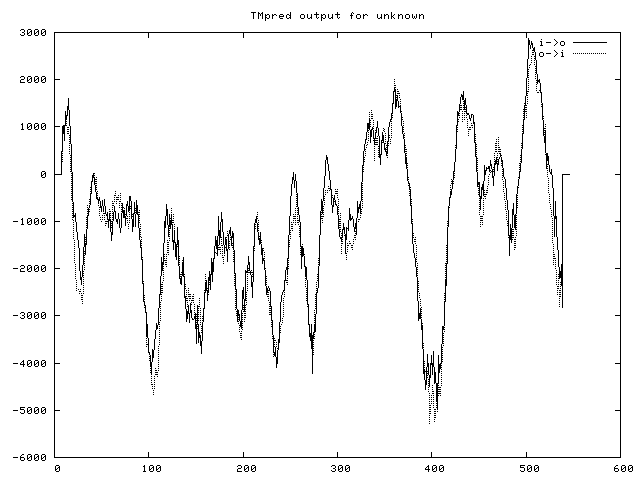
](http://www.ch.embnet.org/wwwtmp/.TMPRED.1193.4862.gif)**(7.3) delta subdivision**

*Pelobacter propionicus* YidC

[
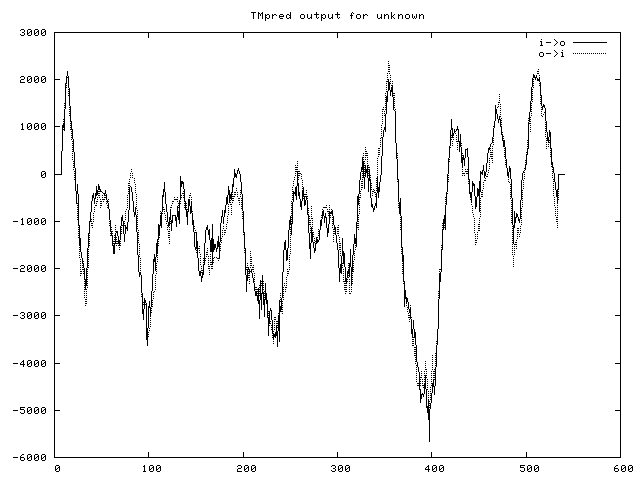
](http://www.ch.embnet.org/wwwtmp/.TMPRED.405.2124.gif)**(7.4) epsilon subdivision**

*Pelobacter propionicus* YidC

[
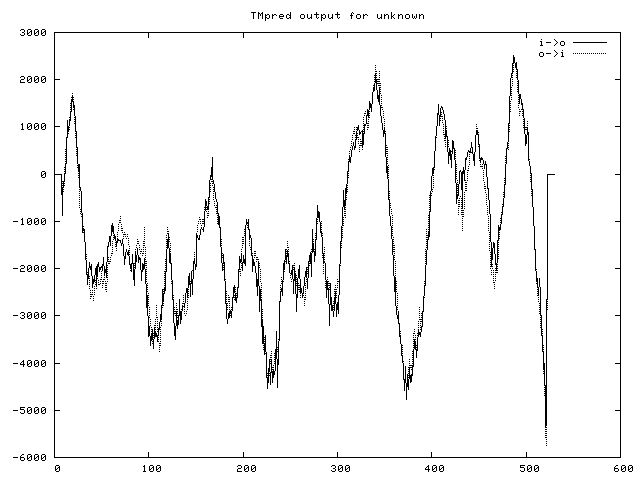
](http://www.ch.embnet.org/wwwtmp/.TMPRED.409.7498.gif)**(7.5) gamma subdivision**

*Xanthomonas campestris* YidC

[
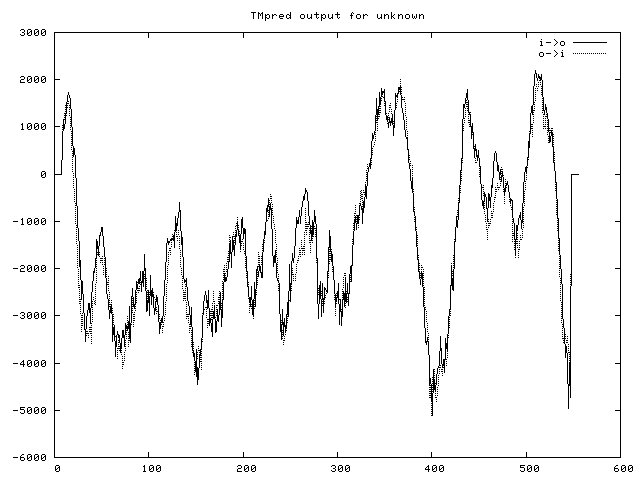
](http://www.ch.embnet.org/wwwtmp/.TMPRED.992.8584.gif)**(8) Spirochaetales**

*Leptospira borgpetersenii* YidC

[
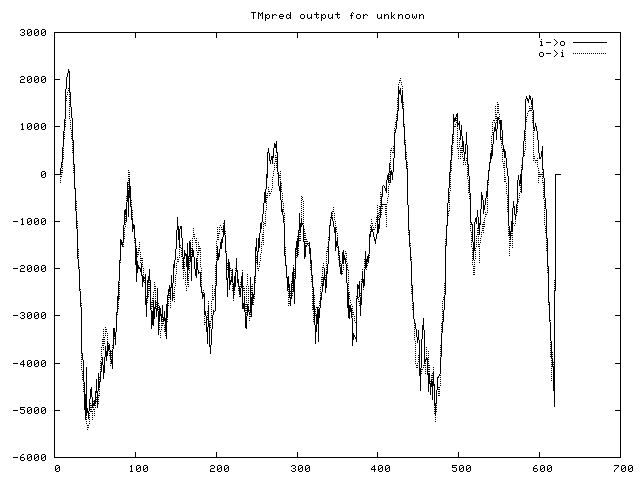
](http://www.ch.embnet.org/wwwtmp/.TMPRED.1019.9256.gif)
