# Supplementary material for: The evolution of YidC/Oxa/Alb3 family in the three domains of life: a phylogenomic analysis
Source: BMC Evol Biol. 2009 Jun 18;9:137. doi: 10.1186/1471-2148-9-137 (PMC2706819; doi:10.1186/1471-2148-9-137)
Supplement: Additional file 3 — Targeting prediction for green algae and plants Oxa2 proteins with particular TPR domain. The subcellular locations were predicted by MitoProt II. [file 1471-2148-9-137-S3.doc]

**Additional file 3**

| **Homologs** | **Probability of Mitoprot II** |
| --- | --- |
| *Ostreococcus tauri* Oxa2 | 0.8902 |
| Ostreococcus lucimarinus Oxa2 | 0.7083 |
| *Arabidopsis thaliana* Oxa2-1 | 0.9904 |
| *Oryza sativa* Oxa2 | 0.9950 |
| *Populus trichocarp* Oxa2 | 0.9984 |
