# Supplementary material for: The evolution of YidC/Oxa/Alb3 family in the three domains of life: a phylogenomic analysis
Source: BMC Evol Biol. 2009 Jun 18;9:137. doi: 10.1186/1471-2148-9-137 (PMC2706819; doi:10.1186/1471-2148-9-137)
Supplement: Additional file 4 — Comparison of alternative tree topology regarding the endosymbiosis origin of Oxa, Alb3 and the secondary loss of Alb3 in plants. The approximately unbiased (AU) test, bootstrap probability (BP), and unweighted and weighted (W) Kishino-Hasegawa (KH) and Shimodaira-Hasegawa (SH) tests were used, all calculated using CONSEL. [file 1471-2148-9-137-S4.doc]

**Additional file 4**

1. Comparison of alternative tree topology regarding the endosymbiosis origin of Oxa and Alb3.

(1) Alternative tree topologies.

| T1 | ML tree (Fig 1) |
| --- | --- |
| T2 | Oxa as sisters to Proteobacteria |
| T3 | Oxa as sisters to Alpha-proteobacteria |
| T4 | Proteobacteria as sisters to Oxa |
| T5 | Alpha-proteobacteria as sisters to Oxa |
| T6 | Cyanobacteria as sisters to Alb3 |
| T7 | Alb3 as sisters to Cyanobacteria |

(2) Comparison of alternative tree topology with *P* values of AU, BP, KH, SH, WKH and WSH tests.

| Rank | Item | Obs | AU | BP | KH | SH | WKH | WSH |
| --- | --- | --- | --- | --- | --- | --- | --- | --- |
| 1 | T1 | -11.1 | 0.889 | 0.865 | 0.861 | 0.993 | 0.861 | 0.996 |
| 2 | T7 | 11.1 | 0.154 | 0.132 | 0.139 | 0.464 | 0.139 | 0.367 |
| 3 | T6 | 20.5 | 0.009 | 0.003 | 0.022 | 0.230 | 0.022 | 0.073 |
| 4 | T2 | 30.1 | 0.002 | 5e-05 | 0.002 | 0.060 | 0.002 | 0.003 |
| 5 | T4 | 37.0 | 0.001 | 1e-04 | 0.004 | 0.030 | 0.004 | 0.011 |
| 6 | T3 | 42.3 | 2e-04 | 0 | 0.001 | 0.006 | 0.001 | 0.002 |
| 7 | T5 | 98.0 | 2e-06 | 0 | 0 |  | 0 | 0 |

1. Comparison of alternative tree topology regarding the evolution of Alb3 in algae and plants.
2. Alternative tree topology.

| T1 | ML tree (Fig 2) |
| --- | --- |
| T2 | Algae-alb3.2 as sisters to Plant |
| T3 | Algae3.1 and 3.2 are sisters |

1. Comparison of alternative tree topology with *P* values of AU, BP, KH, SH, WKH and WSH tests.

| Rank | Item | Obs | AU | BP | KH | SH | WKH | WSH |
| --- | --- | --- | --- | --- | --- | --- | --- | --- |
| 1 | T1 | -1.8 | 0.645 | 0.617 | 0.615 | 0.753 | 0.615 | 0.726 |
| 2 | T2 | 1.8 | 0.415 | 0.374 | 0.385 | 0.483 | 0.385 | 0.517 |
| 3 | T3 | 6.0 | 0.028 | 0.009 | 0.085 | 0.162 | 0.085 | 0.169 |
