# Supplementary material for: The evolution of YidC/Oxa/Alb3 family in the three domains of life: a phylogenomic analysis
Source: BMC Evol Biol. 2009 Jun 18;9:137. doi: 10.1186/1471-2148-9-137 (PMC2706819; doi:10.1186/1471-2148-9-137)
Supplement: Additional file 6 — Protein identities (%) between mitochondria Oxa1 and Oxa2 sequences from plants and green algae. The sequence identities of Oxa1 are in bold-face, and the sequence identities of the Oxa2 are in italic. [file 1471-2148-9-137-S6.doc]

**Additional file 6**

|  |  | (1) | (2) | (3) | (4) | (5) | (6) | (7) | (8) | (9) |
| --- | --- | --- | --- | --- | --- | --- | --- | --- | --- | --- |
| Oxa1 | *Ostreococcus tauri* Oxa1 |  |  |  |  |  |  |  |  |  |
|  | Ostreococcus lucimarinus Oxa1 | **85** |  |  |  |  |  |  |  |  |
|  | *Arabidopsis thaliana* Oxa1-1 | **29** | **31** |  |  |  |  |  |  |  |
|  | *Oryza sativa* Oxa1 | **30** | **31** | **40** |  |  |  |  |  |  |
|  | *Populus trichocarp* Oxa1-1 | **26** | **30** | **50** | **37** |  |  |  |  |  |
| Oxa2 | *Ostreococcus tauri* Oxa2 | 12 | 12 | 11 | 11 | 11 |  |  |  |  |
|  | Ostreococcus lucimarinus Oxa2 | 14 | 14 | 11 | 14 | 12 | *71* |  |  |  |
|  | *Arabidopsis thaliana* Oxa2-1 | 14 | 14 | 13 | 15 | 14 | *17* | *15* |  |  |
|  | *Oryza sativa* Oxa2 | 15 | 16 | 14 | 14 | 14 | *13* | *15* | *30* |  |
|  | *Populus trichocarp* Oxa2 | 16 | 16 | 14 | 14 | 15 | *16* | *17* | *45* | *31* |
