# Supplementary material for: The evolution of YidC/Oxa/Alb3 family in the three domains of life: a phylogenomic analysis
Source: BMC Evol Biol. 2009 Jun 18;9:137. doi: 10.1186/1471-2148-9-137 (PMC2706819; doi:10.1186/1471-2148-9-137)
Supplement: Additional file 7 — Multiple sequence alignments used in this study. Multiple sequence alignments used for Figure 1, Figure 2, Figure 3 and Additional file 5 were shown. [file 1471-2148-9-137-S7.doc]

**Additional file 7**

1. Page 2-13: multiple sequence alignments for Fig 1.

2. Page 14-22: multiple sequence alignments for Fig 2.

3. Page 23-30: multiple sequence alignments for Fig 3.

4. Page 31-41: multiple sequence alignments for Supplemental material S5.

79 526

Aga_Oxa2 ---------- ---------- ---------- ---------- ---------- ----------

Dme_Oxa2 M--------- ---------- ---------- ---------- ---------- ----------

Hwa_YidC M--------- ---------- ---------- ---------- ---------- ----------

Nph_YidC MQW------- --DVSDDVSW LEK------- ---------- ---------- ----------

Tko_YidC M--------- ---------- ---------- ---------- ---------- ----------

AthAlb3.b M--------- ----VRRSRF SHT------- ---------- ---------- ----------

PAlb3.b-2 ---------- ---------- ---------- ---------- ---------- ----------

PAlb3.b-1 ---------- ---------- ---------- ---------- ---------- ----------

OsaAlb3.b M--------- ----VRRPAS ARR------- ---------- ---------- ----------

AthAlb3.a MGT------- QFLPRNKLFT TST------- ---------- ---------- ----------

OsaAlb3.a ML-------- ----LRRHGG RRA------- ---------- ---------- ----------

PAlb3.a-1 M--------- ----LRRFIS TRI------- ---------- ---------- ----------

PAlb3.a-2 M--------- ----LRRFIS TRI------- ---------- ---------- ----------

CreAlb3.2 ---------- ---------- ---------- ---------- ---------- ----------

OtaAlb3.2 ---------- ---------- ---------- ---------- ---------- ----------

OluAlb3.2 ---------- ---------- ---------- ---------- ---------- ----------

Hal_YidC MKW------- ----KDELSW IET------- ---------- ---------- ----------

Hma_YidC MSW------- ----SDELTW IEK------- ---------- ---------- ----------

Mla_YidC MTW------- ----SDDLTW IES------- ---------- ---------- ----------

CalOxa1 MSP------- ---------- ---------- ---------- ---------- ----------

CalOxa2 ---------- ---------- ---------- ---------- ---------- ----------

Cfa_Oxa2 M--------- ---------- --G------- ---------- ---------- ----------

Hsa_Oxa2 M--------- ---------- ---------- ---------- ---------- ----------

CreAlb3.1 M--------- ---ALQPTLA ASS------- ---------- ---------- ----------

Ctr_YidC MYT-STKLVS AFLVFSNYGG ALAEINLPFR TDENASVVRE IEFDRNMVKH HPYNALFPSH

Cca_YidC RWI-SNPLVT SYLIVSQESG SIEGINLPFS SEDNKSIVNE IGFDRELKAQ VPSEASFPGL

Cch_YidC MEQTVNDLLD GFDL------ ---------- ---------- ------LGKQ LSYTV--RYR

Cph_YidC MEKLTNELLN AFDL------ ---------- ---------- ------IKNS LSYTV--TYR

Pma3_YidC ---------- ---------- ---------- ---------- ---------- ----------

Syn3_YidC ---------- ---------- ---------- ---------- ---------- ----------

Syn4_YidC ---------- ---------- ---------- ---------- ---------- ----------

Pma1_YidC ---------- ---------- ---------- ---------- ---------- ----------

Pma2_YidC ---------- ---------- ---------- ---------- ---------- ----------

Syn2_YidC M--------- ---------- ---------- ---------- ---------- ----------

Cya_YidC M--------- ---------- ---------- ---------- ---------- ----------

Tri_YidC M--------- ---------- ---------- ---------- ---------- ----------

Nos_YidC M--------- ---------- ---------- ---------- ---------- ----------

Syn1_YidC M--------- ---------- ---------- ---------- ---------- ----------

Lga_YidC1 M--------- ---------- ---------- ---------- ---------- ----------

Ljo_YidC1 M--------- ---------- ---------- ---------- ---------- ----------

Lga_YidC2 ---------- ---------- ---------- ---------- ---------- ----------

Ljo_YidC2 ---------- ---------- ---------- ---------- ---------- ----------

Lac_YidC2 ---------- ---------- ---------- ---------- ---------- ----------

Lac_YidC1 M--------- ---------- ---------- ---------- ---------- ----------

Ota_Alb3.1 ---------- ---------- ---------- ---------- ---------- ----------

Olu_Alb3.1 ---------- ---------- ---------- ---------- ---------- ----------

Sus_YidC MAFINTDLYR GLDL------ ---------- ---------- ------VNSV FPLGV---TY

Aba_YidC METVENAVSN DLEL------ ---------- ---------- ------VNRE YPLTPGELTF

Lin_YidC ITYVRTPLVE AQV------- ---------- ---------- ---------- --------HS

Yli_Oxa1 MQA------- ---------- ---------- ---------- ---------- ----------

Yli_Oxa2 ---------- ---------- ---------- ---------- ---------- ----------

Cdi_YidC M--------- ---------- ---------- ---------- ---------- ----------

Cdi_YidC2 M--------- ---------- ---------- ---------- ---------- ----------

Sco_YidC L--------- ---------- ---------- ---------- ---------- ----------

Sav_YidC L--------- ---------- ---------- ---------- ---------- ----------

Fra_YidC M--------- ---------- ---------- ---------- ---------- ----------

Pbe_YidC ARLIETPTLT TLA------- ---------- ---------- --IVL-LSPR MRKTP-VIVT

Rpa_YidC MRVIETPRLQ TVD------- ---------- ---------- --IEL-FSPS AENVSPVVLR

Ehr_YidC MRVLSNKEIN TPD------- ---------- ---------- --IIL-LSPE IEKINTVTLT

Wol_YidC MRILTNNMVN EPD------- ---------- ---------- --VVL-LSPA AEKTKAVNLF

Ori_YidC MRVIMTNKLG DVL------- ---------- ---------- --VEL-LSPS AEDSDTTTFT

Bsu_YidC1 ---------- ---------- ---------- ---------- ---------- ----------

Gka_YidC1 ---------- ---------- ---------- ---------- ---------- ----------

Bsu_YidC2 ---------- ---------- ---------- ---------- ---------- ----------

Lpn_YidC MAILTSDVLQ QTK------- ---------- ---------- --VKL-LTED ATLVV--PFV

Gka_YidC2 ---------- ---------- ---------- ---------- ---------- ----------

Cfa_Oxa1 ---------- ---------- ---------- ---------- ---------- ----------

Hsa_Oxa1 M--------- ---------- ---------- ---------- ---------- ----------

Mba_YidC MTFITTDLLN EHD------- ---------- ---------- --FDL-FD-- AQLKV--RLE

Dar_YidC MRIVKTDLVK AAE------- ---------- ---------- --FQL-FEDS AQLEV--KLT

Mja_YidC ---------- ---------- ---------- ---------- ---------- ----------

Lbo_YidC MPVQKEKITP KKS------- ---------- ---------- --F-I-VTDS YDFTG--PNN

Dde_YidC MEVVDTPLYK SIE------- ---------- ---------- --VEL-VTPE APKGE--LVF

Cfe_YidC MKIVQGKTYE FKD------- ---------- ---------- --ISL-VSLN PLPAN--VVL

Cla_YidC MEIAVSEHFD YKD------- ---------- ---------- --INL-VDTS PLNTT--LIL

Lpn_YidC MLIVKTDVLD SVE------- ---------- ---------- --FPL-LQNQ ANLIV--TLN

Ppr_YidC -DVVRTGLYN KNL------- ---------- ---------- --VVL-GSDA LTKQL--VFT

Aga_Oxa1 M--------- ---------- ---------- ---------- ---------- ----------

Dme_Oxa1 MSRLY----- ---------- ---------- ---------- ---------- ----------

---------- ---------- ---------- ---------- ---------- ----------

---------- ---------- ---------- ---------- ---------- ----------

---------- ---------- ---------- ---------- ---------- ----------

---------- ---------- ---------- ------DILS ---------- ----------

---------- ---------- ---------- ---------- ---------- ----------

---------- ---------- ---------- ------PSSS ---------- ----------

---------- ---------- ---------- ---------- ---------- ----------

---------- ---------- ---------- ---------- ---------- ----------

---------- ---------- ---------- ------VAPR ---------- ----------

---------- ---------- ---------- ------TVRF ---------- ----------

---------- ---------- ---------- ------SAAG ---------- ----------

---------- ---------- ---------- ------KLSL ---------- ----------

---------- ---------- ---------- ------KLSL ---------- ----------

---------- ---------- ---------- ---------- ---------- ----------

---------- ---------- ---------- ---------- ---------- ----------

---------- ---------- ---------- ---------- ---------- ----------

---------- ---------- ---------- ------GVLS ---------- ---TDNVAD-

---------- ---------- ---------- ------GILP ---------- ----------

---------- ---------- ---------- ------GLLD ---------- ----------

---------- ---------- ---------- ------ITTS ---------- ----------

---------- ---------- ---------- ---------- ---------- ----------

---------- ---------- ---------- ------CWRA ---------- ----------

---------- ---------- ---------- ------ARTL ---------- ----------

---------- ---------- ---------- ------ASAA ---------- ----------

AYYTNQRRIT KTYSFGPYCL NLALQIDGDS RGSIYPDWIC NSNGFLGMIL DPLPGYMAYQ

PSVGNDGSIK KTYKLQPYAF EVEVGVNRAS DDGVYPQWIL NSNGYFGIIL SPYPGYETLK

LDVAPQKAIE IAYLFESYAI DYDVKLIGFG NDSGEATWVG VRNKYFTAAL IPAALKMSLT

LDVAPEKSIT IVYTFDVYGI NYDVQFTGFD GVSGVAGWIA VRSKYFVASL IPAALKFTLK

---------- ---------- ---------- ---------- ---------- ----------

---------- ---------- ---------- ---------- ---------- ----------

---------- ---------- ---------- ---------- ---------- ----------

---------- ---------- ---------- ---------- ---------- ----------

---------- ---------- ---------- ---------- ---------- ----------

---------- ---------- ---------- ---------- ---------- ----------

---------- ---------- ---------- ---------- ---------- ----------

---------- ---------- ---------- ---------- ---------- ----------

---------- ---------- ---------- ---------- ---------- ----------

---------- ---------- ---------- ---------- ---------- ----------

---------- ---------- ---------- ---------- ---------- ----------

---------- ---------- ---------- ---------- ---------- ----------

---------- ---------- ---------- ---------- ---------- ----------

---------- ---------- ---------- ---------- ---------- ----------

---------- ---------- ---------- ---------- ---------- ----------

---------- ---------- ---------- ---------- ---------- ----------

---------- ---------- ---------- ---------- ---------- ----------

---------- ---------- ---------- ---------- ---------- ----------

EYSDGHVVVR KVVRFDSYLS MVSCEVTLDG KPEGTFSFAG IADTYFAAVF LPPLPLSGVD

EF-DGDLSAK KTLRFNGYVV GVETSVTRGG SNRGPFNWAG AQDQYFAALF LPPVDVIGSL

IGHRTPFVLA KRYVFDNYMF ELHVSLSERE DSREQVDWAS VSGKYFALIV LPIA-FVRRP

---------- ---------- ---------- ---------- ---------- ----------

---------- ---------- ---------- ---------- ---------- ----------

---------- ---------- ---------- ---------- ---------- ----------

---------- ---------- ---------- ---------- ---------- ----------

---------- ---------- ---------- ---------- ---------- ----------

---------- ---------- ---------- ---------- ---------- ----------

---------- ---------- ---------- ---------- ---------- ----------

-RQEGDIQYR ATLAVEQFMF RVTYEVTNGS GERGSGGWVG ITDKYWLSAA IPLLETQSIA

YDNGEGLTFR RTIAVERYLF TMKDEVSNIG NAKVTNGWLG ITDKYWASAL LPLLDPQVVG

WDNQQGVIFQ IKVTLDYYMF KIEQIIINNT DNTDDNQWVG IADKYWFTAL IPSHDSYSAK

WNNENGIVFR MKVSLNNYMF KVEQIVENNT KDTSKKNWFG FADKYWLTAI IPVKPYKNIT

WTNKHNVQFI VSVSIDNYMF TINQSIVNNS KQLNVVHWLG ISDKYWLTSF IPLSPKEVVG

---------- ---------- ---------- ---------- ---------- ----------

---------- ---------- ---------- ---------- ---------- ----------

---------- ---------- ---------- ---------- ---------- ----------

WNGPNGVSIR RIFTLGSYAI SIKDEVINKS DAQITGGWVA LLQHHFFTAW IPRGPAFTVQ

---------- ---------- ---------- ---------- ---------- ----------

---------- ---------- --------MH SVVGPLPWPR ---------- --KPPTAGLL

------ARKF LSRGCGSYGA KASPLPGKMH SVAGPSQWLG ---------- --KPLTTRLL

SADQNGIKVA KILTFGSYLI DIAWEVANGS DKTADNGWLA MVQHYFVSAW VPIVPVAEIA

APDANGVKVS KVYTFNSYEI GVRYDIVNGG AATTTDGWVA MLQHYFMSAW ILLVDYATVK

---------- ---------- ---------- ---------- ---------- ----------

HSSDRSYQLK KEFRFSENYF KLSISII--A YLESGVDFAG TGSRYFIAVA DPVYDNILLE

VGEMDGVRFE RTLSFETYLI SEKVRLTDTA GVDEGVNWGG VMCNYFMAVM APENSSLSIS

TQALNGVTLT KTITFPDGAY DLKVKLSKNE DYFTGVDILA NSDRYYTTLF YESAENTLSS

TQNLNNLVVT KKITFKYGNY DLEVNLSKDA AYFSNVTLMA ASDRYYSAFF YNNHENSIVA

GKSEDGLDVK KEFVFGSYLI EVNYKIANTG NSDAKGGWIA MQQHYFLSAW VPVSQPITVE

HNSGQGFTVR KIYTFDSYGI KLDTQVFNNM AVDKNLQWSG FADKYFLTAI LSSSPRITVQ

---------- ---------- ---------- ---------- ---VHRGRDY HIASGHATAT

---------- ---------- ---------- ---------- LQRITRGSSL PLAAPEKWSN

---------- ---------- -----SAPVA Y-VQQGMINL -HDLT----- --GLPWWATV

---------- -----STEVQ ----GSTPVA Y-MQDVLIKI -HDYS----- --GLPWWASI

---------- -------KKM Y---WQEARQ V-IAGIVNIV -IGFAAL--- --GLPF-FAL

---------- -----PEGTY D---YSPIKN A-IGGTINVV -LGLDSI--- ---MPF-YVV

---------- ---------- --------LE G-IYAFLDNL -FGFITG--- --YHPM-WVI

---------- -----AETFG W---FSGIAN Y-METILKVL -KDLSTV--- HVPYSYGFAI

---------- ---------V W---LSGITY G-LESTLKVL -KDLSAV--- HLPYAYGFAI

---------- ---------V W---LSGITS C-LESTLKVL -KDLSAL--- HVPYAYGFAI

---------- -----APEVG W---LSGITN S-METVLKVL -KDLSAL--- HVPYPYGFAI

---------- -----ADSVS W---FGFISD A-MELVLKIL -KDLSAV--- HVPYAYGFAI

---------- -----GGGGG W---FGFISE A-LEVVLKVL -KDLSAV--- HVPYSYGFAI

---------- -----ASAAS W---FGFISD G-MEFVLKVL -KDLSSV--- HVPYAYGFAI

---------- -----ASAAT W---FGFISD G-MEFVLKVL -KDLSAV--- HVPYAYGFAI

---------- ---------V P---IDVLAQ F-FEFVLQTL -DELESA--- KIPYSYGFAI

---------- ---------A W---LGPITD A-LEGALRGI -DVLDG---- KVPYSYGYSI

---------- ---------- W---LGPITD G-LESALEGI -DVLDG---- RVPYSYGFSI

---------- -----DDESY D---WKPLRD L-IGTVMDVP -LGLEEL--- ---LPF-YAV

---------- -----DADSW D---FDSVRE P-LADALNLL -LGLLDL--- ---LPF-FAV

---------- -----DEGSW D---LTSVRD V-IGGVIDIV -LGLNDM--- ---LPF-YVV

---------- -----AIVS- ----DWGPTS L-IERLLEVT -HV------- YTGLPWWGTI

---------- ---------- M---HNAIIN T-MTSSFQTV -HEFS----- --GLPWWALI

---------- -----VAGSG W---ASAPVR G-AEEMLLGL -HAAA----- --GLPWWACI

---------- -----WAVAV W---ASSPVR V-AEEVLLGV -HAAT----- --GLPWWGSI

---------- -----AVMPS W---VAPVAD A-LEQVLYAL -QELDKL--- HVPYSYGYSI

GGLMNFRFFA GPFDDETLKK W---FTFISE P-FAKFLLIL -MKFFH---- YLTNSWALSI

EGTHRFLVYA GPLADPTLRA F---FAFITE P-FAALLFII -MKFFRM--- -ITGSWGISI

EVHNTFTMYV GPLDYNTVKG L---RPFAEW M-ILPVFNWL -N-------- GFISNYGIII

THSESLVLFV GPVDYNVLHN L---RPFAEY I-ILPIFDLL -S-------- KLTSNYGLII

---------- ---------- ----IGFISE KLLIPILDFF -Y-------- GLVPSYGLAI

---------- ---------- ----IGYISD NLLLPILDFF -Y-------- GLVPSYGLAI

---------- ---------- ----IGYISD NLLIPILDFF -Y-------- GLVPSYGLAI

---------- ---------- ----IGYISD NILLPILDFF -Y-------- GLVPSYGLAI

---------- ---------- ----IGYISD NILLPILDFF -Y-------- GLVPSYGLAI

---------- ---------- ----IGFIST NIMLPILDFF -F-------- GIVHSYGFAI

---------- ---------- ----IGFIST NIMLPILDFF -Y-------- GIVPSYGFAI

---------- ---------- ----IGFISN NIMLPILDFF -Y-------- GIVPSYGLAI

---------- ---------- ----IGFLSN NVMLPIIDFF -Y-------- GIVPSYGLAI

---------- ---------- ----VGFLSN NVMLPILDFF -Y-------- GIVPSYGLAI

---------- ---------K V---FSLFGK P-IQNIMLAV -EHI------ GGSNGAGWAI

---------- ---------K I---FSLFGK P-IQNIMLAV -EHI------ GGSNGAGWAI

---------- -------MRP W---DRYIVY Y-ISQFILIA -S-------- LVRDSYGWAI

---------- -------MRP W---DRYIVY Y-ISQFILIA -S-------- LVHDSYGWAI

---------- -------MRP W---DRWIVY Y-MSAFILLA -K-------- LMGNSYGWAI

---------- -------NNP V---YQWLGR P-LQNIMIQT -AHI------ GGENGAGWGI

---------- ---------A W---LAPVSN A-LEDLLFTI -KGLLDL--- GVPYPTGNAI

---------- ---------A W---LAPVSD A-LEDLLFAI -QGLQGL--- GVPYSTGNAI

GPPIRFELFV GPKDVDMLRN W---LSVLAK P-LFLIVNYV -N----D--- TLVHNFGWAI

SGPTVTRLFV GPKALHILEN Y---LGFIAK P-LFLWLRWT -HD------- HWVPNWGWSI

AVADVYRVYI GPCAEQYLSA I---LYPLEV -LLKWLLRLF -Y-------- TLIPNWGVAI

EAALDTVKVL DKQTHIQQVS THPTWLWPSD I-YLNLLEHV -HV------- YTGLPWWAAI

---------- ---------- L---TVDIVR P-VETALNAI -HDFS----- --GLPWWAVI

---------- ---------- ----LNFIYW P-ISAILWFW -HKVSFVLDP GSGLSWVLAI

---------- ---------- ----LEMFIY P-VSGVMRLW -HYFADLFGC SQSQAWVASL

---------- ---------- ----FSFITW P-VSWVIVQF -HTYGAIFGP DTGWAWGLSI

---------- ---------- ----FSFITI P-VSWVIVQF -HSYGKVFGA DTGWAWGLSI

---------- ---------- ----LDPLYH L-AANAIVFF -HKFGPIFGA DSFFAWAFSV

TIRTEAYLYG GSKEVEVLRQ H---FSFLTR P-IFGILAFF -E-------- GLTGNWGVAI

TGSANTRLFA GAKEAGVVGQ W---FYFITK P-MFVALDFF -Y-------- HLVGNFGLSI

TASSTSYLFA GAKELKLLDD I---LYFITK P-VFLLLEYF -Y-------- DLIGNFGLAI

SASNVNYFFA GAKKLNLLDT V---LYFITK P-VFLLLEYF -N-------- FVLKNFGLAI

SLSVVHHLFL GAKEVKLLDK W---FYILTK P-LFYTLSFF -Y-------- KYCGNFGVSI

---------- ---------P W---DKYVVY P-LSELITVA -K-------- LTGDNYGLSI

---------- ---------P W---NEYIVY P-LSWLIKVA -G-------- LLGGSFGLSI

---------- --------MF F---HDYLIE P-FSALLKVA -G-------- LFHGEYGLSI

SASTEARLWV GPKLVSLLAD R---FSIIGQ G-LFWVLSHL -H-------- SFLHNWGWAI

---------- ---------K W---NHYFVY P-MSKLLLLG -H-------- WFGDNYGIAI

LAEAQVQAAT PSPTAVTEVA SGVAEYTPVG L-IQNLLEFM -HVN------ -LGLPWWGAI

FAEVQVQAAT PSPTAVPEVA SGVAEYTPVG L-IQNLLEFM -HVD------ -LGLPWWGAI

KGEASVSLYA GPQMQSALKA W---LTVVAA P-IFWALEAI -HK------- -LVGNWGWAI

SLSVSVPLYA GPEEYNIISA I---FYIFAS P-LFWLLVKL -HG------- -LVSNWGWAI

---------- ---------- M---FGSIFD I-YYKTLDAI -F-------- MIIKHPALAI

NYNLDFASYI GIRESEGMAQ I---TTPFRN G-IIWILKQI YR-------- FTIPNYGWSI

SAEFGIGYYI GPKESDRLAP W---FTFLAK P-LVSGLKFF -Y-------- SYAGNYGVAI

SGSFSTKGYI GPKDHKILKD W---FTFIAK P-MFLLLSWL -H-------- NYIGNWGFAI

SNTFKASGYI GSKEHDILRD W---FTFIAK P-MYEFLDFL -H-------- GYLGNWGWAI

DKIVGSKLYI GPEITSVLKS I---LWFLSS L-LFSLMKAI -Y-------- TVVGNWGWSI

SVTVVHRLFV GPKDIDILKG W---FTVIAK P-LLYTLKYF -Y-------- RYVGNYGVAI

LSTADGDKTI PEPPAIPQAG APIAEWTPVG I-VQNCMEFL -HIG------ -LDLPWWGCI

LQAVPKTEDI PAAPVPPPAD GLVGEWSPVG M-VQNCLEFL -HCT------ -WDIPWWGTI

ILT-TVGLRT LVTLPLAVYQ NKILA----R LEQISLEMP- ---ELIKELK AEAMK-KFWT

VLS-TFLFRS VVTLPLTIYQ HKITA----R IEKIALEMP- ---AIVEELK KEAKH-KFWS

VLILATITG- FY-SSLIQKY TIDYE----K MAENQAKTK- ---EFQAKFR EAGDE-LIK-

ILVLAVLTG- LY-TTILQAN LTDME----K MSKYQEQAQ- ---ELQDKMS AAGDD-AVE-

TVAGAIIGG- -T-YTLIYYF FTDIE----K QRHMQKLAK- ---ELQKEMR EAGDEKKLK-

ILL-TVLVKA -ATFPLTKKQ VESAM----A MKSLTPQIK- ---AIQERYA GDQ-----E-

ILL-TVLVKA -ATFPLSKKQ VESAM----A MRSLQPQIK- ---AIQQRYA GDQ-----E-

ILL-TVLVKA -ATFPLSKKQ VESAM----A MRSLQPQIK- ---AVQQLYA GDQ-----E-

ILL-TVLVKA -ATFPLTKKQ VESAI----A MRSLQPQVK- ---AIQERYA GDQ-----E-

ILL-TIIVKA -ATYPLTKQQ VESTL----A MQNLQPKIK- ---AIQQRYA GNQ-----E-

ILL-TVIVKA -ATLPLTKQQ VESTL----A MQNLQPQIK- ---AIQQRYA GNQ-----E-

ILL-TIAVKV -ATLPLTKKQ VESTL----A MQNLQPKIK- ---AIQQRYA GNQ-----E-

ILL-TVFVKV -ATLPLTKKQ VESTL----A MQNLQPKIK- ---AIQQRYA GNQ-----E-

IAL-TVLVKV -ATFPLTQKQ VESTL----S LQALQPRVK- ---ELQAKYA DDP-----E-

LLL-TVLVKL -ATFPLSKQQ VESSI----Q MQAMQPRIK- ---ELQAMYA NDP-----E-

IVL-TVLVKL -ATFPLSKKQ VESSM----Q MQAMQPRIK- ---ELQAMYA NDP-----E-

VMILALATG- LY-STLLQAN LMNME----K MSAYQSRMK- ---EIQERRK EAGDD-ALD-

VMVLALFTG- LY-STLLQSN LMDME----V MGKYQGRMK- ---EIQERRK EAGDD-ALE-

ILVLAIITG- TT-STILQDN LMDMS----G MGDHQEKME- ---DLKERRK AADDQ-ALD-

VVA-TIAVRL -VLFPLYVRA SSNAT----K MSKIKPQID- ---ELLQQIK TGDTV-DQM-

PLT-TFTLRS VWTLPLAILQ RKRIQ----K QSQLRPLVS- ---AMNPILK LNRVQ-QAIN

GLG-TVALRG AVTLPLAAYQ HYILA----K VENLQPEIK- ---NIARHLN QERAN-QLWS

LLS-TVALRG AVTLPLAAYQ HYILA----K VENLQPEIK- ---TIARHLN QERAN-QLWS

ILL-TLIVKL -LTYPLTKQQ VESAM----A VQALKPRID- ---LIKDRFG EDK-----D-

VLL-TVSLRL -MLYPLNTWS TKSMV----R MQQIAPQVT- ---AIQEKYK KDP-----K-

ILL-TVFLKL -LLYPLNAWS IRSMR----R MQKLSPYIQ- ---EIQQKYK KEP-----K-

IIF-AFLVKL -VTYPLSMAS TKSMK----K MAALQPVLQ- ---ELQVKYK DNP-----A-

IIF-ALLIKL -VTYPLTMAS TKSMK----K MAALQPMMK- ---ELQEKYK DNP-----Q-

VAL-TVVIRI -ALFPLSAGS IRSAR----R MKIAQPVMKT RQAEIKSKFS GDP-----K-

VAL-TVVIRL -ALFPLSAGS IRSAR----R MRIAQPVMQK RQAEIKARFA SNP-----Q-

VAL-TVVIRL -ALFPLSAGS IRSAR----R MRIAQPVMQK RQAEIKARYA NDP-----Q-

VAL-TVVIRL -ALFPLSAGS IRSAR----R MRIAQPAMKK RQDEIKSRYA KDP-----Q-

VAL-TVVIRL -ALFPLSAGS IRSAR----R MRIAQPAMKK RQDEIKSRYA KDP-----Q-

IAL-TLVIRL -GLYPLSAGQ IRNMR----K MRITQPLMKE RQEEIQKRYK DDP-----A-

IAL-TLVIRF -GLYPLSAGQ IRNMR----K MRITQPLMKE RQAEIQRRYK DDP-----T-

VAL-TLVIRA -SLYPLNAGS IRNMR----K MKVTQPLMKK RQEEIQKRYK DDP-----Q-

VAL-TLIVRF -ALYPLSAGS IRNMR----K MRIVQPLMQK RMAEIKERYK DEP-----Q-

IFL-TLVIRF -ALYPLNVGS IRNMR----R MKVINPLMQR RMREIQEKYR DDP-----Q-

III-TFVVQL -IVMPLRLAS QRKMTTQQEK TQKLQPQMK- ---LIQEALK KPTQP-QQM-

III-TFVVQL -IVMPLRLAS QRKMTTQQEK TQKLQPQMR- ---LIQEALK KPTQP-QQM-

IIF-TIIVRI -ILLPLNAIS IKSMA----K QQKVQPQMD- ---ALRKKYP GKVES--RQ-

IIF-TIIVRI -ILLPLNAIS IRSMA----K QQKVQPQMD- ---ALRKKYP GKVES--RQ-

IVF-TIIVRV -ILLPLNAIS IRSTT----K MQSIQPQIN- ---ELRKKYP GRTES--RT-

VII-TFVVRL -ILMPLMLVQ QNKSVRQQEK MARLQPQMK- ---LIQNAMK HKTPD-QQM-

IIV-TILVKM -VTYPLTKDQ VVSSL----N MKNLQPQIA- ---AIREKYE DDQ-----E-

IIV-TILVKF -VTYPLTRDQ VVSSL----N MKNLQPQIA- ---AIREKYE DDQ-----E-

VLV-TIAINF -ILFPLKLSN MKSMR----K MQALKPQVD- ---AINAKYK NVRDP-RAA-

IIL-TVIINL -VLLPLRLSS MKSAL----K MQKIQPQMK- ---AIQEKYK KYNDP-KRA-

ILV-TIAIKV -LFFPLTKRS FIAMQ----K MQELQPHMQ- ---RIQERYK GNT-----Q-

AST-TVIVRV -LLFPLFVQA ANEQG----K MSEVKPELN- ---VIDEKLK SAMT--EMQ-

PLV-TLTLRS TVTLPIAIST RLRAQ----K QHELRPLIS- ---ALGPILR AKNAN-KALT

VLL-TFTIRA -LLVKPMLNQ MRSMR----K MQELQPLMQ- ---EIRKKYP NDQ-------

FAL-VVTVRS -IIAPFSWMQ FKSGR----F AIMMRPKIK- ---RLKEEYA EDK-----E-

VSL-VILIRI -CLIPLFVKQ IKATR----G MQTLQPEMK- ---KIQERYK NDK-----Q-

VSL-VILIRI -CLIPLFVKQ IKATR----A MQTLQPEMK- ---KIQERYK NDK-----Q-

VLL-VICVRI -LIFPLFVKQ VKSQR----T MQMMQPRIK- ---EIKEKYG HDK-----Q-

LLL-TLVIKA -VLFPLANMS YKSMA----G MKKVQPELM- ---KIRERYT DDK-----T-

LFV-TVIIKL -LFLPLANKS YASMA----K MKAIQPQLQ- ---ALKDRHP DDK-----A-

LLL-TIVIKL -IMFPLSYKS YVSMF----K LKYLQPEIL- ---KIKELYK NDN-----T-

LLL-TLVIKL -LMLPLSNRS YVSMF----K MKSLQPEVA- ---RIKELYK NDS-----L-

LIV-TILIKI -MMFSFSNRS YSSMK----K MRDLQPRIQ- ---RLQELYG DDK-----I-

ILV-TILIRL -LILPLMIKQ LRSSK----A MQALQPEMQ- ---KLKEKYS SKQKT--QQ-

IVV-TILIRL -LILPLMIQQ TRNAK----A MQALQPEIE- ---ALRKKYS SKAQT--QQ-

ILV-TIIVRI -VVLPLFVNQ FKKQRIFQEK MAVIKPQVD- ---SIQVKLK KTPEK--QK-

IGL-VVLLRL -ALYPLSAAQ YKSGA----K MRRFQPRLA- ---QLKERYG DDR-----Q-

IVL-TLIVRF -CLLPLILKQ FRASL----A MQKLRPELL- ---KLQEKYK SKPET--QR-

AAC-TVLARC -LVFPLIVKG QREAA----K IHNHLPEIQ- ---KFSTRIR EAGDQ-EFY-

AAC-TVFARC -LIFPLIVTG QREAA----R IHNHLPEIQ- ---KFSSRIR EAGDH-EYY-

VVL-TIMIKA -VFFPLSAAS YKSMA----K MKMLTPRLA- ---QLKERFG D-----DKQ-

VLL-TLTVKA -VFYPLTAAS YRSMA----K MKALAPRLE- ---RLKAQHG D-----DRM-

LII-AIIVS- -LIINIATKL LVDQK----R VAELKKEIQ- ---EFQVKFK KKNPE-MME-

IIF-AILFKL -VFYPLNQKQ ADSMK----K MQELSPQLK- ---TINEKFA ND-----PK-

IIL-TILVKL -LFWPLSQKS YKSME----Q MKKLQPMVQ- ---KIKEKYG DD-----RQ-

VAL-TIVIRI -VLFPLTYKG MVSMN----K LKDLAPKMK- ---EIQAKYK GD-----PS-

VIM-TLIVRI -ILFPLTYKS MISMN----K LKDLAPKMK- ---EIRERYK GD-----PQ-

VLV-TVLIKL -AFYRLSATS YKSMA----S MRKLQPKLQ- ---ALRERYG DD-----KA-

III-TIILKA -LFFPLTHKS YKSMK----D MQKIQPMMA- ---ALKEKYK DD-----RE-

AIG-TVCVRT -LLFPLVIAS QRNAA----K MNNYMPQLQ- ---VLQMKMT EAGNA-DSA-

AIG-TLAVRT -IIFPLVILA QRNSA----K MNNNMPQMQ- ---MLQLKMT EAGNA-ESA-

EKARIMYNHS LKKQWNNLIV RENCHPAK-- TMV----LLW GQIPLWIVQS VAIRNLVSLP

EKTQIVYRRS IKKQWQNLIV RDNCHPMK-- TMI----VLW GQIPLWIFQS VALRNLVYLP

---------K MQARQQAMMA DQMEMSKN-- QFKPMAYILV LTVPIFFWLI EHIPTEAQ--

---------R LREEQMEAFG DQASMMKE-- QFRPMVWIML LTIPVFLWMY WKLGTGT---

---------K VQKKQMELMK MQSELMQQ-- QMIPM----F LTLPVFWIFF AWLRRGI---

---------K IQLETARLYK LAGINPLA-- GCL----PTL ATIPVWIGLY RALSNVAD--

---------R IQLETARLYK LAGINPLA-- GCL----PTL ATIPVWIGLY RALSNVAN--

---------R IQLETARLYK LAGINPLA-- GCL----PTL ATIPVWIGLY RALSNVAN--

---------R IQLETARLYK LSDVDPLA-- GCL----PTL VTIPVWIGLY RALSNVAN--

---------R IQLETSRLYK QAGVNPLA-- GCL----PTL ATIPVWIGLY QALSNVAN--

---------R IQLETARLYK QAGVNPLA-- GCF----PTL ATIPVWIGLY QALSNVAN--

---------R IQLETSRLYR QAGVNPLA-- GCF----PTL ATIPVWIGLY QALSNVAN--

---------R IQLETSRLYR QAGVNPLA-- GCF----PTL ATIPVWIGLY QALSNVAN--

---------N LQLETARLYK EAGVNPLA-- GCF----PTL ATIPVFIGLY NALSNAAK--

---------R LQLEQARLYR EAGFNPLA-- GCL----PLF ATLPVFIGLY RALSNAAA--

---------R LQMEQARLYK EAGFNPLA-- GCL----PVF ATLPVFIGLY RALSNAAS--

---------A IQDEQMDAMG DQLGMFKE-- QFRPMVWIMF LTIPVFLWMY WSIGVGAN--

---------R IQEEQMEAMA DNLGMFKE-- QFRPMVWIML LTIPVFLWMY AVVGFRGA--

---------R LEEEQMELMT DQMGMFKQ-- QFRPMVWIML VNIPLFLWLY WIVFGAGV--

---------R AMEKRRLIMK ENGVSTLA-- TLF----P-A VQLPLAYGFF QALRKMAN--

KEILLLSAKE ARKRQKELFA KNGVQLWK-- NFI----LPA FQVPLWIMMS ITMRDLSGSS

KRARLTYLKN MRRLVSELYV RDNCHPFK-- ATI----LVW IQFPMWIFMS VALRNFSTA-

KRARLTYLKN MRRLISELYV RDNCHPFK-- ATV----LVW IQLPMWIFMS FALRNLSTA-

---------K IQKETSVLYE QAGVNPLA-- GCL----PTL ATIPIFIGLF SSLTNVAN--

---------K AQLEIMSLYR ERGVNPAS-- GCL----PLL IQMPFLIGMF DLLKSSFA--

---------R AQMEVMALYK TNKVNPIT-- GCL----PLL IQLPFLIVMF DLLKSSFL--

---------K MQSELSRIYR EAGVNPVG-- GCL----PTL LQMPLLFAMF YVFRSSIQ--

---------K MQSELGRIYK EAGVNPLG-- GCL----PVL LQMPLLFAMF YVFRSSIE--

---------K QQEELGKLMN EFG-SPLA-- GCL----PLI VQMPILFALF ATLRGSPFAD

---------K QQEELGKLMK EFG-SPLA-- GCL----PLL VQMPILFALF ATLRGSPFAD

---------K QQAELGKLMK EFG-SPLA-- GCL----PLL VQMPILFALF ATLRGSPFAD

---------K QQEELGKVMK EFG-NPLS-- GCL----PLL VQMPILFALF ATLRGSPFAD

---------K QQEELGKVMK EFG-NPLS-- GCL----PLL VQMPILFALF ATLRGSPFAD

---------K QQEEMAKVMK EFG-NPLA-- GCL----PLL LQMPILFALF ATLRGSPFSD

---------K QQEEMGKLMQ EFG-NPLA-- GCL----PLL LQMPILFALF ATLRGSPFAN

---------K QQEEMGKLMK EF--NPLA-- GCL----PLL FQMPILFALF ATLRGSPFTN

---------K QQEEMVNVQK EFG-NPLA-- GCF----PLL VQMPVLLALF ATLRGSPFAS

---------K LREAQAKLYS ELGVNPLG-- GCL----PLL IQMPVLFALF ATLRGSPFAA

---------Q ISQLQMRVYK DNNMSMMGGM GCL----PLL IQLPIMIGIY QAVAYSKE--

---------Q ISQLQMRVYK ENNMSMMGGM GCL----PLL IQLPIMMGIY QAVAYSKE--

---------K LQEETSKLYK EAGINPYT-- GCL----PML IQLPVMYALY QAIWRTPQ--

---------K LQEETSKLYK EAGINPYT-- GCL----PML IQLPVMYALY QAIWRTPQ--

---------L LQQETNKLYK EAGVNPYT-- GCL----PVI IQLPVMYALY GAILRTPQ--

---------T LSGWQRELYS KNQVSLTGGI GCL----PLL IQLPIMWGIY QAVFYSQE--

---------R MNKEINRVYE ENGVNPLA-- GCG----PAL LSFPVLAGLY RAFNNAGI--

---------R MNKEINRVYE ENGVNPLA-- GCG----PAL LTFPVLAGLY RAFNNAGI--

---------D KNQETMDLYK KHGVNPMG-- GCL----PMV LQIPFFFAFY KVFTVSVE--

---------D MNTEMAALYK QHSVNPVG-- GCL----PLV IQMPFLIAFY GMLAVAIE--

---------K IHEEMAKLYR EAQYNPLS-- GCL----PTL VQMPIIFAMY RLFNNYFE--

---------M VAHEKKKILK KYGISQMK-- LFY-----PM AMFPLTIGIF LGIRRMCEIG

APIEMLAMKE RRKRRVKLYK EHGCEMWK-- SLI----GPL VQLPIWITMS LAVRAMCGT-

---------K LMEETRKLQK EMGVNPVA-- GCL----PVL VQMPVFIGLF HVLRSFNRTG

---------S ILEQQKEIQE EYGYSMAA-- GCV----PAL IQVPVFLGLY QVLLRMAR--

---------R QSEEMMKLYK ETGTNPLS-- SCL----PIL AQSPFFFALY HVLNGIASGD

---------R QSEEMMKLYK ESGTNPLS-- SCL----PIL AQSPFFFALY HVLNGIATGK

---------Q MQLEIMKLQK EHG-NPLL-- GCL----PIL LQIPLFISLF HVFTHLAP--

---------K QQQEMMALYK KHKINPAA-- GCL----PVL AQMPIFYALY KTLFVTIE--

---------K QQQEMMEIYR KEKINPVA-- GCL----PVL LQIPVFFSLY KVLFVTIE--

---------K MSKEVSLLFK KNDVSPMS-- GFI----PIL IQIPVFFALY KVLFVTIE--

---------K QHKETIALFK KNNVNPMS-- SIL----PIL VQIPVFFALY KVLFVTIE--

---------K LHQEIMALYK KEKVSLAG-- SFL----QSL IQLPIFFSLY KVLHVTIE--

---------K LQQETMALFQ KHGVNPLA-- GCF----PIL IQMPILIGFY HAIMRTQA--

---------K LQQEMMLLFQ KHGVNPMA-- GCF----PIL IQMPILIGFY HAIMRTRE--

---------E LQMEMMKLYQ EHNINPLA-M GCL----PML IQSPIMIGLY YAIRSTPE--

---------K YQQATMELFK KEKINPMG-- GCL----PLL IQMPIFFALY WVLVESVE--

---------K LQQEMMQLYQ KHGVNPAS-- GCL----PVL IQMPIFMALY YAISRTQE--

---------K ASSEMTFYQK KHDVKLFR-- PLI----LPL TQAPIFISFF IALREMAN--

---------K ASSEMALYQK KHGIKLYK-- PLI----LPV TQAPIFISFF IALREMAN--

---------R LNQEMMKLYQ TEKVNPLG-- GCL----PIL VQIPVFIALY WVLLGAVE--

---------K FQQAVMEMYK TEKVNPLG-- GCL----PML IQIPVFIGLY WALLASVE--

---------K LQEEQQRIMQ LNAELMKM-- SFR----PMI YTILIFIYLR HVYGFGGV--

---------M RQQKTMELYK KNNVNPVG-- GCL----PMV IQIPIFIALY TAFSDTID--

---------R MNQEVMELYK TYKVNPAG-- GCL----PML LQIPVFLGLY QGLLNAIE--

---------K LNAHVMELYK KNGANPMG-- GCL----PIL IQIPIFFAIY RVLLNAIE--

---------K MNLHMMELYK KHGANPMS-- GCL----PIL IQIPIFFAIY RVLLNAIE--

---------K ISQATMELYK QEKVNPLG-- GCL----PIL IQIPVFIALY WVLLESVE--

---------G MNKAVMELYR DHKVNPLG-- GCL----PML VQIPVFFALY KALMFSIE--

---------R YGQEMVLFMK EKNLNPLK-- NML----VPL AQAPIFISFF MGLREMAN--

---------R YAQEMMLFMR EKGVNPLK-- NMV----VPL AQAPLFISFF MGLRQMAN--

DIA----ATE L--------- ---------- ---------- ---------- ----------

DIA----VTE M--------- ---------- ---------- ---------- ----------

---------- M--------- ---------- ---------- ---------- ----------

---------- ---------- ---------- ---------- ---------- ----------

---------- ---------- ---------- ---------- ---------- ----------

--------EG L--------- ---------- ---------- ---------- ----------

--------EG L--------- ---------- ---------- ---------- ----------

--------EG L--------- ---------- ---------- ---------- ----------

--------EG L--------- ---------- ---------- ---------- ----------

--------EG L--------- ---------- ---------- ---------- ----------

--------EG L--------- ---------- ---------- ---------- ----------

--------EG V--------- ---------- ---------- ---------- ----------

--------EG V--------- ---------- ---------- ---------- ----------

--------EG L--------- ---------- ---------- ---------- ----------

--------EH L--------- ---------- ---------- ---------- ----------

--------EG L--------- ---------- ---------- ---------- ----------

---------- ---------- ---------- ---------- ---------- ----------

---------- ---------- ---------- ---------- ---------- ----------

---------- ---------- ---------- ---------- ---------- ----------

-------NEG F--------- ---------- ---------- ---------- ----------

-TN----LPS L--------- ---------- ---------- ---------- ----------

-EG----VEQ L--------- ---------- ---------- ---------- ----------

-EG----VEQ L--------- ---------- ---------- ---------- ----------

--------DG L--------- ---------- ---------- ---------- ----------

---------- L--------- ---------- ---------- ---------- ----------

---------- L--------- ---------- ---------- ---------- ----------

---------- L--------- ---------- ---------- ---------- ----------

---------- L--------- ---------- ---------- ---------- ----------

VPYNINLKPD QIAAIDPKPY KSPRHSIFIT EKSHFPVIAT IPNGTKLGTE ESIKINLQTT

VPYTLNLKPE QIAAVDPKPF NSASHSIFVT ETDHVPVIAS LPGGTKIGAG ENVQIQLQTK

VPYTLNLKPD QIAAVEPKPF TSASHSIFIT ETNHVPIVAS LPGGTKLGTG DSVPIQLETR

VPYLVNLKPD QIAAIEPKPF TSSKHSIFIS ETKHFPVIAS LPSGTKLGVG DKAQIKLQTL

VPYLVNLKPD QIAAIEPKPF TSSKHSIFIS ETKHFPVIAS LQSGTKLGVG DKAQIKLQTL

INYTVDLQPE QVERIVPQTF STKPQNIYVD EALHYPIAVF LPGGKMLGVG EKTQLEIQST

INYTVDVQPE QIERVVPQPY ATKTNNIYVS DGVHYPIAAL LPGGNKLGVG EKTNIEFQTS

INYTVDLQPE QLERIQPQAY TTSPQNIYIN DGVHYQILAL SPNGDKLIVG DKTKVEFQTV

ANYSVNLQPE QIEQIQPQAF ATAPQNIYVA DGEHVKVAAI LPSGNKLAVG EQTKIQYQTV

VTYDVNLQPE VAAEVVPTPY VSPSKNIFVT DSVHKPVVLV APKGTKIAVG EQVQFRLQGP

---------- L--------- ---------- ---------- ---------- ----------

---------- L--------- ---------- ---------- ---------- ----------

---------- L--------- ---------- ---------- ---------- ----------

---------- L--------- ---------- ---------- ---------- ----------

---------- L--------- ---------- ---------- ---------- ----------

---------- L--------- ---------- ---------- ---------- ----------

--------DG A--------- ---------- ---------- ---------- ----------

--------DG A--------- ---------- ---------- ---------- ----------

---------- M--------- ---------- ---------- ---------- ----------

---------- L--------- ---------- ---------- ---------- ----------

---------- F--------- ---------- ---------- ---------- ----------

-------VQG L--------- ---------- ---------- ---------- ----------

-VG----VKS M--------- ---------- ---------- ---------- ----------

L--GLEV--- ---------- ---------- ---------- ---------- ----------

---------- K--------- ---------- ---------- ---------- ----------

T--------- ---------- ---------- ---------- ---------- ----------

T--------- ---------- ---------- ---------- ---------- ----------

---------- M--------- ---------- ---------- ---------- ----------

---------- L--------- ---------- ---------- ---------- ----------

---------- M--------- ---------- ---------- ---------- ----------

---------- M--------- ---------- ---------- ---------- ----------

---------- M--------- ---------- ---------- ---------- ----------

---------- M--------- ---------- ---------- ---------- ----------

---------- I--------- ---------- ---------- ---------- ----------

---------- I--------- ---------- ---------- ---------- ----------

---------- I--------- ---------- ---------- ---------- ----------

---------- L--------- ---------- ---------- ---------- ----------

---------- I--------- ---------- ---------- ---------- ----------

--------PS L--------- ---------- ---------- ---------- ----------

--------PS L--------- ---------- ---------- ---------- ----------

---------- M--------- ---------- ---------- ---------- ----------

---------- L--------- ---------- ---------- ---------- ----------

---------- ---------- ---------- ---------- ---------- ----------

---------- L--------- ---------- ---------- ---------- ----------

---------- L--------- ---------- ---------- ---------- ----------

---------- L--------- ---------- ---------- ---------- ----------

---------- L--------- ---------- ---------- ---------- ----------

---------- L--------- ---------- ---------- ---------- ----------

---------- L--------- ---------- ---------- ---------- ----------

-------PES M--------- ---------- ---------- ---------- ----------

-------PES M--------- ---------- ---------- ---------- ----------

---------- ---TLGGF-- ---------- ---------- ------GWIP NLTELHS---

---------- ---TIGGF-- ---------- ---------- ------GWIP NLTVVNS---

---------- ---LADP--- ---------- ---------- ---------- --SLATA---

---------- ---------- ---------- ---------- ---------- -----IN---

---------- ---------- ---------- ---------- ---------- ----------

---------- ---LTEGF-- ---------- ---------- ------FWIP SLAGPTAFIE

---------- ---LTEGF-- ---------- ---------- ------FWIP SLAGPTSFVD

---------- ---LTEGF-- ---------- ---------- ------FWIP SLAGPTEFVD

---------- ---LTEGF-- ---------- ---------- ------FWIP SLAGPTAFTD

---------- ---FTEGF-- ---------- ---------- ------FWIP SLGGPTAFVD

---------- ---LTEGF-- ---------- ---------- ------FWIP SLGGPTAFVD

---------- ---LTEGF-- ---------- ---------- ------FWIP SLGGPTAFVD

---------- ---LTEGF-- ---------- ---------- ------FWIP SLGGPTAFVD

---------- ---LTEGF-- ---------- ---------- ------FWIP SLGGPT-FEN

---------- ---LDDGF-- ---------- ---------- ------YWIP SLGGPTAFVD

---------- ---LTDGF-- ---------- ---------- ------YWIP SLGGPTSFVD

---------- ------A--- ---------- ---------- ---------- --EVSLT---

---------- -----PL--- ---------- ---------- ---------- --YIELP---

---------- ---------- ---------- ---------- ---------- -----DS---

---------- ---SDQGY-- ---------- ---------- ------AWFQ NLIEVDPY--

---------- ---YEEGI-- ---------- ---------- ------LWFQ DLSIADPM--

---------- ---ATDGI-- ---------- ---------- ------LWFP DLTALDST--

---------- ---ATGGI-- ---------- ---------- ------LWFP DLTAPDST--

---------- ---LTQGF-- ---------- ---------- ------YFVP SLAGPTMLPD

---------- ---RGAPFI- ---------- ---------- -----SGWID DLTAPDSW--

---------- ---RGASFI- ---------- ---------- -----PGWID NLTAPDSWT-

---------- ---RQHGF-- ---------- ---------- ------LWAK DLSVPDPLGD

---------- ---RQQSF-- ---------- ---------- ------LWAK DLSLPDPLGD

NGNSYSEVLS KYDNGSKFLP SWKVSKGSEN IKVSQDGTVT AIKPGDLFIK ALGQVGFYV-

SGEPFSNVLS DVENGQSFLP SWSVTKGESV VSVSEDGTIQ ALAPGDLFIK ALGQVGFYT-

GGESFRNVIS GVDNGQRFLP AWTVTKGEGV VSVSETGTIT ALTTGDLFIK ALGQVGFYT-

TGESFTSRLS GVEGGTKFTP TWSVTKGDDL VKVSADGTVQ ALAEGDLFIK ALGQVGFYV-

TGESFTSRLS GVEGGTKFTP TWSVTKGDDL VKVSADGTIQ ALSEGDLFIK ALGQVGFYV-

EGKAFNQVIP EK-NSQILTP TYSVTKGEDR ISVNPDGTIE ALVPGDLFIK ALGQVGVTE-

EGKSLSKLVE QN-PESDIQP TYEVTKGEER VKINEDGTIE ALAPGELFIK ALGQVGVVD-

EGKSLNTLME EY-NQSEIKP LWTVTKGAER VQIDQDGSLV ALEPGELFIK ALGRVGAFE-

EGKPFQVLLA EH-PENKLTP DWKVTKGEDR IKIDADGNVE ALQPGELFID ALGRVGAQA-

GGKPFEQLVA EAGGDPTLRP TWKITKGEER AQIQPDGTLL ALQPGELFID KLGRVGAFP-

---------- ---AASSF-- ---------- ---------- ------FGI- SLGQRS----

---------- ---AASSF-- ---------- ---------- ------FGI- SLGQRS----

---------- ---QNGRF-- ---------- ---------- ------LWM- DLGRPP----

---------- ---QNGRF-- ---------- ---------- ------LWM- DLGRPP----

---------- ---QTGRF-- ---------- ---------- ------LWM- DLSKPP----

---------- ---AHSTF-- ---------- ---------- ------FGI- SLSQKS----

---------- ---FDEPW-- ---------- ---------- ------FFLP SLAGPTLDAD

---------- ---FDEAW-- ---------- ---------- ------FFLP SLAGPTLDSD

---------- ---RGAPW-- ---------- ---------- ------LWVS DLSQPEP---

---------- ---RQANW-- ---------- ---------- ------FWLH DLSGPDH---

---------- ---RGAMFI- ---------- ---------- -----PYWIP DLSLADQM--

---------- ---STEGV-- ---------- ---------- ------LWFQ NLAAPDPY--

---------- ---GTEGA-- ---------- ---------- ------LWFP DLLMMDH---

---------- ---NRETFS- ---------- ---------- --------AA DLEMYQP---

---------- ---EG----- ---------- ---------- LDTHEPL-TS DLEQLA----

---------- ---------- ---------- ---------- ---IGKINQP EAKAHSKVS-

---------- ---------- ---------- ---------- ---IGVIDDQ AAKAHDKVQ-

---------- ---NEG---- ---------- ---------- ---PGLVWRP RVGLSAAESL

---------- ---RHEPF-- ---------- ---------- ------LYIP DLSEQDFDPT

---------- ---RHAPFY- ---------- ---------- ------GWIH DLSAADYDPT

---------- ---RHAPFY- ---------- ---------- ------LWVK DLSNFDPI--

---------- ---RHAPFC- ---------- ---------- ------LWIK DLSTSDPI--

---------- ---RHAPFF- ---------- ---------- ------IWIK DLSALDWF--

---------- ---SEHSF-- ---------- ---------- ------LWF- DLGEKDP---

---------- ---AQHNF-- ---------- ---------- ------LWF- DLGEKDP---

---------- ---ASHSF-- ---------- ---------- ------LWF- SLGQSD----

---------- ---RQAPWL- ---------- ---------- ------GWIQ DLTARDP---

---------- ---KTHSF-- ---------- ---------- ------LWV- ELGHRDP---

---------- ---QTGGL-- ---------- ---------- ------LWFQ DLTLSDPT--

---------- ---QTGGL-- ---------- ---------- ------WWFQ DLTVSDPI--

---------- ---RGAPWI- ---------- ---------- ------LWIK DLASADP---

---------- ---RGAPWV- ---------- ---------- ------LWYT DLARPDP---

---------- --------Y- ---------- ---------- ---------- ELNG------

---------- ---WNSPFL- ---------- ---------- -------WVK DLSEPDPYQG

---------- ---RHAPFI- ---------- ---------- ------VWLA DLSAKDPF--

---------- ---KGAPWI- ---------- ---------- ------FWIK DLAIMDPY--

---------- ---KAAPWA- ---------- ---------- ------FWIT DLSVMDPW--

---------- ---RQAPFI- ---------- ---------- ------FWIN DLASADPY--

---------- ---RHAPFY- ---------- ---------- ------FWIT DLSGPDPF--

---------- ---RDGGL-- ---------- ---------- ------FWFT DLTICDQF--

---------- ---RDGGL-- ---------- ---------- ------FWFT DLTMADPF--

---------- -LIFPVALGV INLSIIEIQA ASSKL--QTI -FTNLFRGLS ILMV--PIAA

---------- -YILPVALGL INLAIIEVQA MSTRL--QNI -ANNVFRGLS VVMV--PVAC

---------- -IVIPFAGL- ---------- ---------- ---------S TYFE--VYLG

---------- -MVLPLLGI- ---------- ---------- ---------D L-NG--GRVL

---------- --VKPFNFF- ---------- ---------- ---------L F--D--WFHW

HPPLGWPDTA YLVLPLLLVF SQYLSIQIMQ S-SNDPAMKS -SQAVTKLLP LMIG--YFAL

HPPLGWSDTA YLVLPVMLVV SQYISVQIMQ S-SDDPNVKN -SQAITKFLP LMIG--YFSL

QPPLGWSDTA YLVLPAMLVV LQYMSVQIMQ S-SDDPNVKN -SQAIMKFLP LMIG--YFSL

HPPLGWSDTA YLVLPVLLVI SQYVSSQVMQ P-NNDPSQQG -AQAVVKFLP LLIG--YFAL

HPPLGWYDTA YLVLPVLLIA SQYVSMEIMK P-TDDPAQKN -TLLVFKFLP LMIG--YFAL

HPPLGWHDTC YLVLPVLLVA SQFVSMEIMK P-TDDPSQKN -TLLVLKFLP FMIG--WFSL

HPPLGWHDTA YLVLPVLLIA SQYVSMEIMK P-TDDPTQKN -TLLVFKFLP IMIG--YFSL

HPPLGWNDTA YLVLPVLLVV SQYVSMEIMK P-TDDPTQKN -TLLVFKFLP LMIG--YFSL

APPVGWANAA YLVMPVLLVA SQYASQKIIS S-NQDPSQQQ -AQAILKFLP LMIG--WFSL

HPPLGWYETC YLVLPVLLVV SQFVSQTIIS PQTDDPAQQQ -SQAILKFLP FMIG--FFSL

HPPLGWHDTA YLVLPVLLVA SQYVSQQIVS PQTDDPAQQQ -SQAILKFLP LMIG--FFSL

---------- -LVLPLVGEV ---------- ---------- ---------- AWTD--SVLG

---------- -VVMPIAGEV ---------- ---------- ---------- DWTH--GLLG

---------- -ITLPIFGEV E--------- ---------- ---------- AWSN--SVAG

---------- -LGLQAISAA AIIAVVRVGG ETAMAA---G -MKKVMTVVP IASI--FITK

---------- -HVFPVILGI TALCNIEWTL KTKFRPTLTD AFGNLTKMSI VFMM--AISL

---------- -WILPISVGV INLLIVEIFA LQSRF--QTY -ITYFVRAVS VLMI--PVAA

---------- -WILPISVGV INLLIVEICA LQSRF--QTY -ITYFVRAMS VLMI--PIAA

APPIGWEDAA YLTLPLLLVA VQYASSSVTS PPPKDENANT -QRALLVFLP LMVG--WFSL

------SK-E FHLLPILLGL VMFLQQRFMS SGDQQRQ--- -QRAMGTMMT VVFA--VMFY

--------TE FHLLPILLGI VMFAQQKISA SKDQQRQ--- -QETMGTMMA LLFT--FMFY

---------H IAFFPILMAG TVYLQQKITP TAQPNEQM-- --KIMLVLFP VMML--FFFN

---------H IALFPILMGG AVYLQQKITP TAQSNDQM-- --KAMLYIFP VMML--LFFN

----GAINWD IATLVGAFGL TLLLSQVLSS QG--NPQ--- -QSTANKITP IMITGMFLFF

----GSINWD IAILVGAFGL SLFISQLLSG MG--NPQ--- -QATANKITP VMITGMFLFF

----GAINWD IAILIGAFGL SLFVSQLLSG MG--NPQ--- -QSTANKITP VMITGMFLFF

----GEINWD IAILVGGFGL TLLVSQILSG RG--NPQ--- -QSTANKITP VMITGMFLFF

----GEINWD IAILVGGFGL TLLVSQILSG RG--NPQ--- -QSTANKITP VMITGMFLFF

----GEINWD ILGMIVFFGF SIYLNQELSG ASAQAQQ--- -QQTINKITP ILFSGMFLFF

----GTINYD ILGMILFFGA SIYINQELSG SSA--QQ--- -QQAINKITP LLFSGMFLFF

----GNIHWD ILIMVIGFGV SIYASQTISG KGNANPN--- -QDSINKITP ILFSGIFLFT

----GTIHWD IVAMIVFFGI SLYVSQMLSG QNGGNPQ--- -QDTVNKITP VIFSGMFLFF

----GTIHWD IIGMIVLFGV SIYLNQSLTN AG-EDPS--- -QSSMARITP VLFSAMFLFF

---------- --VLTIIATL LYVIQGYLSM VGEQKKAM-- --QMTLILSP AMTF--FISI

---------- --ILTIIATL LYVVQGYLSM VGEQKKAM-- --QMTLILSP AMTF--FISI

---------- -YIMPVLAAA FTFISTYISQ MSSQNGM--- -TKGMTYLMP LIIG--VSAI

---------- -YIMPILAAV FTFMSTYISQ MSSQNGM--- -TKGMTYLMP LIIG--VSAI

---------- -YIMPILAMV FTFLSTYISQ LSSQNGM--- -TKVMMYGMS IMVG--VMAL

---------- --VLAIIATV FTVIQGYIST IGEQKKTM-- --QSMMLFNP IMTL--FFSL

APPIGWDDAL YLLFPIMTTL SQFVSMEVLK PEEKTKEMQN -QSVLLKLLP FFIG--YISL

APPIGWEEAL YLIFPVLTTI SQFVSMEVLK PEEKTDEMKN -QSVLLKLLP LFIG--YISL

---------- -IILPLVMIA SQFVMQRMTP Q-PGDPAQ-- --QKMMMFMP LVFG--FMFY

---------- -LIIPVLIVA STILMQRMTP Q-AIDPAQQ- ---KMMIMMP AFLG--WISL

---------- -RMLPVLYVV SQIMFSKLTQ VP-HTEQ--- -QKIMTYVMP LFFF--FFFY

---------- -LGLQVITAA MYMASIRLGS ETNLSPG--- -MKKILQWAP WISV--PFLM

---------- -SVLPAAVGI ITLLNVELTT KASEGPKLPK MMANFARVGA IALF--SIAA

---VDFRTIA LVALIVIIVL ATHFNARLSV DRQMDMM--- -NRMMLWFMP LTIL--FTGV

---HGTDQPH AFVLPLIIAA CVFTTIVSTL RTGMYRF--- -LIVMVLVAP IGLL--QTGI

-LGALTVRVI TAVMIVLMSA SQFFTQRQLM TKPFMQQ--- -QKMLMYVFP VMFA--VFGI

-LGALMVRVV TAIMIVLMSA SQFYTQRQLM TKPFMQQ--- -QKMLMYVFP VMFA--IFGI

FLGAGTAKIL AVVLIVLMGA TTFFTQRQIM ARPQAMV--- -QKILLYGSP LMLA--IFGF

-VPLIFL--G IGILPLLMGA AMFVQLKLNP P-PPDPTQ-- --RMIFGLMP LIFM--FIFA

---IPVLYLV LGIWPIIMGI TMWVQMKLNP A-PPDPTQ-- --QMIFAWMP LLFT--FMLA

---------- IGILPVILGI TMIIQQKMNT STDQDKI--- -QASFIKFLP YVFI--FIFS

---------- IGILPIILGI TMIIQQKIGE KGNKDDI--- -QANVMKFFP YISI--FIFS

---------N IGAWPILTSA TMMLQQKINP A-PADPAQ-- --AQVLKFMP LILL--IVFN

---------- -YILPIVAGV ATFVQQKLMM AGQQNPQ--- -MAMMLWIMP IMII--VFAI

---------- -YILPVIAGV TTFIQQKIMM AGQQNPQ--- -MAMMLWMMP IMIV--IFAI

---------- --LMSLSAGI MYFVQAYIAQ KLPQNPSA-- --KLMVFIFP VMMT--IFSL

---------- -YILPVLNIA IMWATQKLTP T-PMDPMQ-- --AKMMQFMP LVFG--VMMA

---------- -YILPVLAAL TTFISLRLSP SMEQMPQ--- -MAMMLYIMP VMIF--IGAS

---------- -YILPLVVTA TMWGVLELGA ETSDLQWM-- --RNLIRVMP LAVL--PITI

---------- -YILPLAVTA TMWAVLELGA ETSDLQWM-- --RNVIRMMP LITL--PITM

---------- -YILPVIMMV SMFVQTKLNP TP--DPIQ-- --AKVMMMMP LIFG--FMFF

---------- -YILPVIMAA TMFLQTFLNP PP--DPMQ-- --AKMMKIMP LAFS--ALFF

---------- -VYLPIILSK ILFID----- ---------- ------FWHW LIYG--FIVS

---------G LNLLALLMVG TQVFQTRMTS V-SMDPNQ-- --KMLMYVMP VMML--YIFW

---------- --ITPVVMGA TMLLQQRLTP A--PDPTQ-- --AKIMMFMP VVFT--FMFL

---------- --ILPVLMGA TMFIQQKITP A-NTDPMQ-- --EKIMKFLP LIFT--FFFV

---------- --ILPIFMGL TMFIQQLITP M-AQDPMQ-- --EKIMKFLP LIFT--FFFL

---------- --VLPLIMGA TMLIQQKLNP A--PDPMQ-- --AKVMMFLP ILFT--GLFW

---------- IGPLPLLMGA TMFIQQKMTP S-TMDPMQ-- --AKMMLALP VVFT--FMFL

---------- -YALPIITSL TLFATIELGT DSANMQT--- -AKYILRALP LFIF--PFTI

---------- -YLLPLITSA TLYLTIEIGT DSANMNT--- -MKYVLRALP IVIF--PFTM

SVPSCLCLYW VTSSAYGLGQ NLLLLSPRVR --------RT VGIPAS

TVPSALCVYW VASSSFGLAQ NLLILSPEVR --------RS VGIPKK

FFPLWILWYM LCSLVMSQII RKSLNIGGM- ---------- ------

VFPAWILWYM MCSFSFSNII RKALNIQTTP --------T- ------

SLLGYVGWYF LTSYAVGMIL RKFLNMG--- ---------- ------

SVPSGLSLYW LTNNILSTAQ QVWLQKYGGA --------KN PVEKFR

SVPSGLSLYW LTNNILSTAQ QVWLQKSGGA --------KN PMMKS-

SVPSGLSLYW LTNNILSTTQ QVWLQKLGGA --------K- ------

SVPSGLSLYW LTNNILSTAQ QVWLQKLGGA --------KN PVKEYK

SVPSGLSIYW LTNNVLSTAQ QVYLRKLGGA --------KP NMDENV

SVPSGLSIYW FTNNILSTAQ QVWLRKLGGA --------KP VVNQGQ

SVPSGLSIYW FTNNVLSTAQ QVWLRKLGGA --------KP VVNENV

SVPSGLSIYW FTNNVLSTAQ QVWLRQLGGA --------KP VVNENV

NVPSGLTLYW FVNNLLSTGQ QLYLKATVKV --------NI PEAIA-

NVPAGLTLYW FFNNIITTAQ TVVLRKITKP --------IE VPTGN-

NVPAGLTLYW FANNIITTAQ TLILRKTTTA --------PE PPVAG-

PIQAWIVWYF LCSMGFTQII RKSLNIDISP --------S- ----AS

PIQVWIVWYF VCSMCFTQVL RKALNIQTTP --------T- ------

PVQAWIVWYF LCSLSFTQII RKALNVETTP --------T- ----G-

GFASAIILYF AVNSIFSLIQ SSLFKSSWFR --------KI AGMPPH

HAPAALTIYW ISSQLYSLLQ NVMMDLMLPI --------SF TPKKRN

TVPSSIVLYW LCSSLMGLSQ NLLLRSPRFR --------QL CRIPLK

TVPSSIVLYW LCSSFVGLSQ NLLLRSPGFR --------QL CRIPSK

NVPAGLSLYY LANTVLSSAI QIYLKKLGGA --------NV VMNELA

NFPSGLNIYW LSSMLLGMVQ QWFVSKQIQK --------TP NPIAVR

NFPSGLNIYW FSSMLLGLIQ QWFTNKILDS --------KH LKNEIR

NMPAGLGLYY LMFNIFSVAQ QFYINKTTTA --------DD MPKVNK

NLPAGLGLYY LMFNVFSIAQ TFYINKTSSA --------DD LPKISQ

PLPAGVLLYM VVANIFQAFQ TFLLNKETLP --------EN LQKIL-

PLPAGVLLYM VIANIFQALQ TFLLTREALP --------DN LQAIL-

PLPAGVLLYM VIANIFQAFQ TFLLTREALP --------DN LQSIL-

PLPAGVLLYM VIANIFQAGQ TFLLSREALP --------EN LQKIL-

PLPAGVLLYM VIANIFQAGQ TFLLSREALP --------EN LQKIL-

PLPAGVLMYI VMANVFQTIQ TLILMREPLP --------EN LQKLLS

PLPAGVLMYI VVANVFQTLQ TLVLMREPLP --------EN LQKLLS

PLPAGVLLYM LIANIFQTIQ AYILSKEPLP --------EN LQKLVA

PLPAGVLMYM VIGNIFQTAQ TYLLSREPLP --------EE LQKIVS

PLPAGVLLYI LVSNIFQTVQ TFLLSREPLP --------EN LQQLVP

SAPGALALYF LVGGLIAILQ QVITTFIIMP --------KV KKDVAD

SAPGALALYF LVGGLIAILQ QLITTFVIMP --------KV KRDVAD

GIQSAISLYW VISNLFQVVQ TFILQNPFKY --------QR ELEEQK

GIQSAISLYW VISNLFQVVQ TFILQNPFKY --------QR ELEEQK

NFQSAITLYW VISNLFQAVQ TFILQNPIKY --------RK EQEAK-

SFSGALALYW AAGNFVMIIQ QLIVTFILTP --------KV KQHVAK

TVPAGLALYW FWNNVFTTGI QVYLRNGG-A ---------- ------

TVPAGLALYW FWNNVFTTGI QVFLRNGG-A --------KA TVE---

NFPSGLVLYY LTSNLVSMGQ QWFFNKTSMA --------TL AAESIK

RYASGLGLYW IVGNIIGFAQ QFIMNKTELG --------RE IAAVRE

DAPSGLLVYW TAMNGVTLVQ QLVMKRTANK --------NK T-----

KMPAALLLHF FVNGILMLIQ GVALRNPFFR --------KK LGIHEK

QTPTAVCLYW ISSSGFSLIQ NVLLNKYMPL --------RE EPPMFH

LWHLGLLFYM VSNNIWTFFQ QRWIFDKIDA --------EI EAKKAK

FIPAAICLYW VANNLWTLIQ NNGMFLALWY --------PY DSDHKD

NFPVGVLVYW LTTNVWTMGQ QMYVIRNNPT PGSKAQAEKA IVKAIK

NFPVGVLVYW LTTNVWTMGQ QMYVIRNNPT PGSKAQAQRA VVKAIK

RFPIAVLLYW LTTNLWSMGQ QFFVIKKMPP LKPLGAGRPA TTSGAR

PFAAGLVLYW FWNTFLSVIQ QYIIMKRQGA --------DV DILGNA

SFPAGLVIYW AWNNLLSVIQ QGYIMRKNGV --------KV ELFDN-

SFPTGLIIYW ICSNSITIIQ QLIIKAYIGR --------KF NLKHD-

SFPAGLVIYW IFSNVITLIQ QSLIKLFLTR --------KL GINVE-

NFPAGLLIYW TWNNILSIVQ QFIINKINNK ---------- ------

NFPAALSLYW VVGNLFMIAQ TFLIKGPDIK --------KN PEPQK-

NFPAALSLYW VVGNIFSIVQ TYLIKGPEIA --------AH S-----

NVPAALPLYW FTSGLFLTVQ NIVLQMTHHK --------SK KTAAL-

FMPAGLVLYW VVNGGLGLLI QWWMIRQHGE --------KP SKIIQ-

SVPSALSLYW VVGGCFSIIQ SLILRSQLKA --------AK AAEN--

HFPTAVFMYW LSSNMFSLGQ VACLRIPAVR --------TI LKIPQG

HFPTAVFMYW LSSNLFSLVQ VSCLRIPAVR --------TV LKIPQG

WFPAGLVLYW VVNNVLSIAQ QWQITRLIDA --------GG KAAND-

FFPAGLVLYY VVNNVLSMAQ QWFINKQIEK --------SN KAALQ-

NTLGWLGWYI LCSFATSTVL RKILGIK--- ---------- ------

NMPSGVTLYW TFQNVLSIGQ QWILKKTEEK --------KK AKV---

NFPAGLVVYW LVNNVLSIGQ QWWMLRKS-- ---------- ------

TFPAGLTLYW FINNLCSVAQ QLVVNKIFKK --------QK EQAIM-

TFPAGLTLYW CVNNICSLIQ QVIVNKLFKN --------HK KEEIA-

NFPSGLVLYW IVNNTLSILQ QWYITRKYSD --------EK PAKKV-

NFPSGLVLYW LLNNILTIGQ QMYINKLVND ---------- ------

NFPGAILCYW ACSNFFSLVQ VGFLRIPKVR --------DF FKIDRR

NFPAAILTYW ACSNFISLGQ VAVLRIPSVR --------EY FKIEKG

65 427

Aga_Oxa1 ---------- ---------- ----TLGLGG -WTPVGIVQN CMEFLHI--- ------GL-D

Dme_Oxa1 ---------- ---------- ---MSIGLGG -WSPVGMVQN CLEFLHC--- ------TW-D

Cal_Oxa2 ---------- ---------- ---MRNFSID HNAIINTMTS SFQTVHE--- ------FS-G

Pin_Oxa2 ---------- ---------- ---MNGTGNE PWAIVQGVQS VLETVHT--- ------TT-G

Pra_Oxa2 ---------- ---------- ---MSTTGHE PWAIVQGVQS VLETVHT--- ------TT-G

Pso_Oxa2 ---------- ---------- ---MSGTGGE PWAIVQGVQS VLEAVHT--- ------TT-G

Aga_Oxa2 ---------- ---------- ----FWQTLS QSAPVAYVQQ GMINLHD--- ------LT-G

Dme_Oxa2 ---------- ---------- ---MYWQTLS NSTPVAYMQD VLIKIHD--- ------YS-G

AthOxa1-1 MAQTLSYRY- ---------- --MQTLAAAD SFFPIAALQQ CIDMVHT--- ------FT-G

POxa1-1 ---------- ---------- ----AIAAAD SFLPVAVLQH AIDAVHN--- ------FT-G

POxa1-2 MARSLDCRD- ---------- --MQAIAAAD SYFPVAALQH VIDAVHS--- ------FT-G

AthOxa1-2 MARGIDCRH- ---------- --MAAIAAAD SAFPVAALQH LIDAVHS--- ------FT-G

AthOxa2-1 ME-------- ---------- -----IPTDD SSLPVLAVVD FLEGFHE--- ------FT-G

Cal_Oxa1 MQ-------- ---------- -----IGLAQ GWGPTSLIER LLEVTHV--- ------YT-G

Cfa_Oxa1 -P-------- ---------- -----ELGLG SYTPVGLIQN LLEFMHV--- ------NL-G

Hsa_Oxa1 MP-------- ---------- -----ELGLG SYTPVGLIQN LLEFMHV--- ------DL-G

Mmu_Oxa1 -S-------- ---------- -----ELGLG SYTPVGLIQN LLEYIHV--- ------DL-G

Cfa_Oxa2 MG-------- ---------- -----YEALA ASAPVRGAEE MLLGLHA--- ------AA-G

Mmu_Oxa2 MG-------- ---------- -----YEALA ASAPVRTAEE VLLGAQE--- ------AT-G

Hsa_Oxa2 MG-------- ---------- -----YEALA ASSPVRVAEE VLLGVHA--- ------AT-G

CreAlb3.1 MSPSDLGDAL HRLG-AIYVL ADAPQRAGGW VAPVADALEQ VLYA----LQ EGLDKLHVP-

Cme_Oxa1 MGVDP--AAL QDSWHTTFAL ASAGAQAGLW NG-FVHLIET IITG----TG DTLAALGVP-

Cme_Oxa2 MT-------- ---------- -----AALSA ERNWYDPVVQ GIIMFQE--- ------YT-D

Cme_Alb3 ---------- ---------- ---------- -------MTA GHEQTKR--- ------SG-G

Pma1_YidC M--------- ---------- ---------I GYISDNILLP ILDFFYG--- ------L-V-

Pma2_YidC M--------- ---------- ---------I GYISDNILLP ILDFFYG--- ------L-V-

Syn_YidC ---------- ---------- -----MDFGV GFLSNNVMLP ILDFFYG--- ------I-V-

Mbr_Oxa1 ---------- ---------- ------GLGS FATPVGLIQN LVEFNTV--- ------ML-D

Mbr_Oxa2 MAAAAVQPAL S-------VG GTRLSLEALT LTGIVDGTEA ALVGLQA--- ------QT-Q

Osa_Oxa1 MALALSHPAT A---PAAAAE AVAPVAAAAD SFFPVAALQH VIDYIHT--- ------FT-G

Osa_Oxa2 MA-------- ---------- -----DGGSA SGFAVSSLID ILDGFHN--- ------LT-G

Olu_Oxa2 ---------- ---------- ---------- -------MSD SMTHLHA--- ------AT-G

Ota_Oxa2 ---------- ---------- ---------- ---------- -MSTLHH--- ------AS-G

Ota_Alb3.1 ---------- ---------- --QPQRMEGW LAPVSNALED LLFT----IK GQLLDLGVP-

Olu_Alb3.1 ---------- ---------- --APQRMEGW LAPVSDALED LLFA----IQ GQLQGLGVP-

Ota_Oxa1 MD-------- ---------- -----PVAVQ SWPTTAALMY AMEYFHV--- ------AH-G

Olu_Oxa1 ---------- ---------- -----PVASQ SWPTTAALMY AMEYFHV--- ------AH-G

Ptr_Alb3-1 MRDL------ ----PQQSLL QDVAESDSGW FGFLTIPIKL LLQA----IH SLLLTVGLNT

Tps_Alb3-1 ---------- ---------- --APNAQNGW FGFLTLPIEG LLKL----IH GGLDSMGMSS

Ptr_Alb3-2 MASVDGL--- ----VQQSAA ATAPSDDGGW WGAYIQLFKT TLNAVHSTIQ GPLQNVGI-E

Ptr_Oxa1 MQ-------- ---------- -----PWDPT WYNLADQAIL AVKSLHE--- ------FS-G

Pin_Oxa1 ---------- ---------- -----SVSDL GYSLSDMAIR SLDVIHS--- ------TT-G

Pra_Oxa1 MV-------- ---------- -----SVSDL GYSLSDIAIR SLDVIHA--- ------TT-G

Pso_Oxa1 ---------- ---------- -----SVSDL GFSLSDIAIR SLDVIHA--- ------TT-G

Sce_Oxa2 MT-------- ---------- -----PSTKR SFSLFQSVAD TFLTVHE--- ------AS-H

Sce_Oxa1 MF-------- ---------- -----GLAQT WYWPSDIIQH VLEAVHV--- ------YS-G

Tps_Oxa1 MS-------- ---------- -----PFEPT W-WPSDQLLL LLNHVHDV-- ------LP-A

Tps_Oxa2 ---------- ---------- ---------- ---------- ---------- ----------

Yli_Oxa1 MD-------- ---------- -----TGSLW SLWPSDIYLN LLEHVHV--- ------YT-G

POxa2 MT-------- ---------- -----DGVVN GILPVDSMIW LLDSYHD--- ------LT-G

Yli_Oxa2 MT-------- ---------- -----VRQLS TVDIVRPVET ALNAIHD--- ------FS-G

AthOxa2-2 ML-------- ---------- -----QDLSN YDYLTQPVIS LLDSYHD--- ------IT-G

Ptr_Oxa2 MW-------- ---------- -----PEVLQ NVSVWGGSGY LLKAIHA--- ------DG-V

Tps_Alb3-2 MM-------- -----QH-QV TLDAKDENSW WESYIQIYKN GLAFVHDNVD EPLRKLGF-D

AthAlb3.b MSPDSFDFIK DHAENLLYTI AD-TTQSNDW FSGIANYMET ILKV----LK DGLSTVHVP-

PAlb3.b-2 --------LF GRAESIVYTI AD-TTQNSDW LSGITYGLES TLKV----LK DGLSAVHLP-

PAlb3.b-1 --------LF GRAESIIYTI AD-TKQNSDW LSGITSCLES TLKV----LK DGLSALHVP-

OsaAlb3.b MDFAEVGELF GRVEAFLYTV AD-KEAAGDW LSGITNSMET VLKV----LK DGLSALHVP-

AthAlb3.a MASVDIGAIF TRAESLLYTI AD-VQKSGGW FGFISDAMEL VLKI----LK DGLSAVHVP-

OsaAlb3.a MAGLDLHAAL ERAEAALYTL ADSAQKNGGW FGFISEALEV VLKV----LK DGLSAVHVP-

PAlb3.a-1 MASVDFNTII SRAEGFLYTL AD-AQKNGGW FGFISDGMEF VLKV----LK DGLSSVHVP-

PAlb3.a-2 MASIDFNSII SRAEGFLYTL AD-AQKSGGW FGFISDGMEF VLKV----LK DGLSAVHVP-

CreAlb3.2 MAVSRRPAVV VKASLLDAAS AALTLAEGGP IDVLAQFFEF VLQT----LD EGLESAKIP-

OtaAlb3.2 MFN--AHAAL VSSPLFDLAA RLAGIQKGGW LGPITDALEG ALRG----ID G-VLDGKVP-

OluAlb3.2 ---------- ---------- --AGLQKGGW LGPITDGLES ALEG----ID S-VLDGRVP-

LPWWGCIAIG TVCVRTL-LF PLVIASQRNA AKMNNYMPQL QVLQMKMTEA RQA-------

IPWWGTIAIG TLAVRTI-IF PLVILAQRNS AKMNNNMPQM QMLQLKMTEA RQS-------

LPWWALIPLT TFTLRSVWTL PLAILQRKRI QKQSQLRPLV SAMNPILKLN LARRQSTIQS

LPWWATLLLS GVTVRAV-IF PFYVLQIQAM QRLIQARPDF SKLHSAYKYA RTFTPGS---

LPWWATLLLS GVTLRAA-VF PFYVFQIQAM QRLMHARPDF SKLYSAYKYA RTFTPGS---

LPWWATLMLS GVTVRAA-IF PFYVFQIQAM QRLVQAKPDF SKLYSAYKYA RTFTPGS---

LPWWATVILT TVGLRTLVTL PLAVYQNKIL ARLEQISLEM PELIKELKAE TAY-AK--NW

LPWWASIVLS TFLFRSVVTL PLTIYQHKIT ARIEKIALEM PAIVEELKKE AAM-AH--KW

FEWWASIVVA TILIRSS-TV PLLIKQMKDT TKLALMRPRL ESIREEMQNK G---------

FNWWASIVVT TLLIRSF-TL PLLINQLKAT SKLSIVRPHL EEVKQRVERQ G---------

FNWWASIIVT TLLIRGA-TV PLLINQLKAT TKLTLMRPHL EEIRQQMSDK A---------

LNWWASIALT TVLIRGV-TI PILLNQLKAT YKLNVLRPQL EELRQEMSTK A---------

LPWWMIIASS TVAVRL-ALL PLLILQLKKL KTISELLPKL PMPIPETPTL KG--------

LPWWGTIVVA TIAVRL-VLF PLYVRASSNA TKMSKIKPQI DELLQQIKTG DTVD------

LPWWGAIAAC TVLARC-LVF PLIVKGQREA AKIHNHLPEI QKFSTRIREA KLA-------

LPWWGAIAAC TVFARC-LIF PLIVTGQREA ARIHNHLPEI QKFSSRIREA KLA-------

LPWWGAIATC TVLARC-LVF PLIVKGQREA AKIHNHMPEM QKFSARIREA KLA-------

LPWWACIGLG TVALRGAVTL PLAAYQHYIL AKVENLQPEI KNIARHLNQE VAVRA--NKW

LPWWSNIILS TVALRGAVTL PLAAYQHYIL AKVENLQPEI KDIAKRLNQE VAVCA--RGW

LPWWGSILLS TVALRGAVTL PLAAYQHYIL AKVENLQPEI KTIARHLNQE VAVRA--NGW

YSYGYSIILL TLIVKL-LTY PLTKQQVESA MAVQALKPRI DLIKDRFGED K---------

GSYGFAIIFL TIIVKA-ITF PLNYKQMKST MAMQALAPKV RELQARYRDN P---------

LPWWATVAAV TVLARI-LVL PLTLNTFRNA ARMQSIKPDV DAIKERMQAA MHS-------

LTLW--LTGS AIALRL-ITL PLEAYQARQW LRLREAQRLA SAAYQLLTRS SQV-------

PSYGLAIVAL TVVIRL-ALF PLSAGSIRSA RRMRIAQPAM KKRQDEIKSR YAKDP-----

PSYGLAIVAL TVVIRL-ALF PLSAGSIRSA RRMRIAQPAM KKRQDEIKSR YAKDP-----

PSYGLAIIFL TLVIRF-ALY PLNVGSIRNM RRMKVINPLM QRRMREIQEK YRDDP-----

APWAVGICAT TILMRT-LML PLVFGSMRNN TILMNIQPQL QLHSQRIREC QTR-------

APWPVAVLLT AGLVRTVVGA PAAWYQAHWS SRVELQRRRL MQWADAIGHQ VMRRKA---N

LNWWACIALA TVLIRS-ATV PLLVNQLKAT QKLNAIRPEM EAIKEEMNA- --MD------

IPWWITISLS TVAMRL-LIL PVLITQIKKA AKIGKLLPEL PPPFPPPLSG RS--------

APWFVALAAS AALVRA-ATA PISARTIKAS ATVSAASALA KSRKRGNSEH V---------

LPWCATLAVS ALCARL-VTA PVAARTTKAS ATVSAASALA KATKQGDAER V---------

YPTGNAIIIV TILVKM-VTY PLTKDQVVSS LNMKNLQPQI AAIREKYEDD Q---------

YSTGNAIIIV TILVKF-VTY PLTRDQVVSS LNMKNLQPQI AAIREKYEDD Q---------

LEWWLAIVGA TVFMRT-VTF PLVVMQMRNT ARMQLCKPEL EALQAKMKSN PSQD------

LEWWLAIVGA TVFMRT-ITF PLIVMQMRNT AKMQLCKPEL EALQAKMKSN PQQD------

NSWGVAIVLL TILIKV-VTF PLTKTQLEST NKMQAMQPAI KELQAKYQSN P---------

NAWGISIIAM TVVIKA-LTF PLTKSQLEST NKMQALQPTI KSLQAKYQSN P---------

QSWGVSIAIF TTIVRT-LLV PLSIEQSKSA EYIKSLKPYV ADIKAKYKNN Q---------

LEYGWSIVGV TVILRL-CLF PVMVASQQTS SRMAHLQPEL QQIKARYEAL GTPS------

LPWWATIIAT TVAVRT-VFF PVTVISMRNA AKMKLFQPDM EKLKAQMDAN PTQS------

LPWWATIIAT TVAVRT-AFF PITVVSMRNA AKMKLFQPDM EKLRDEMDAN PTRD------

LPWWATIIAT TVAVRT-VFF PVTVISMRNA AKMKLFQPDM EKLRDEMDSN PTQT------

IPWIVLVPLT TMTLRTLVTL PFSIWQRRRI LKQQELRKLV QPITPIIKLR LAAVNSSNPL

LPWWGTIAAT TILIRC-LMF PLYVKSSDTV ARNSHIKPEL DALNNKLMST TD--------

YPYAVTIGVT TLAARL-LLF PIYAAGQRNS SRMAHMQPEM KKIMDATPKQ PD--------

------MSLT NICIRT-SLL PIAIQGAKTS VSFGKVAPEV QYLISSFQRD FRLLRE--GM

LPWWAAIAST TVIVRV-LLF PLFVQAANEQ GKMSEVKPEL NVIDEKLKSA AN--------

LPWWIIIASS TLAMRL-TLF PLHVLQMHKI KKISRSFSKL PPLFPPPLSG RS--------

LPWWAVIPLV TLTLRSTVTL PIAISTRLRA QKQHELRPLI SALGPILRAK LAFNAN--A-

LPWWVVIATS TVAFRT-ALL PILILQRKQT KRISQFLPKL PHFWPPQGSG RS--------

VPYWACFAAI NVMVRI-GLF PLVLYGAQTS TRFAKVVPEV QFLLSLFQAD WQRLRQ--N-

QTWGVSIFLF TAGVRA-LLV PFSIQQSKSS EYMKALKPYQ QKIKEKYTDK ----------

YSYGFAIILL TVLVKA-ATF PLTKKQVESA MAMKSLTPQI KAIQERYAGD Q---------

YAYGFAIILL TVLVKA-ATF PLSKKQVESA MAMRSLQPQI KAIQQRYAGD Q---------

YAYGFAIILL TVLVKA-ATF PLSKKQVESA MAMRSLQPQI KAVQQLYAGD Q---------

YPYGFAIILL TVLVKA-ATF PLTKKQVESA IAMRSLQPQV KAIQERYAGD Q---------

YAYGFAIILL TIIVKA-ATY PLTKQQVEST LAMQNLQPKI KAIQQRYAGN Q---------

YSYGFAIILL TVIVKA-ATL PLTKQQVEST LAMQNLQPQI KAIQQRYAGN Q---------

YAYGFAIILL TIAVKV-ATL PLTKKQVEST LAMQNLQPKI KAIQQRYAGN Q---------

YAYGFAIILL TVFVKV-ATL PLTKKQVEST LAMQNLQPKI KAIQQRYAGN Q---------

YSYGFAIIAL TVLVKV-ATF PLTQKQVEST LSLQALQPRV KELQAKYADD P---------

YSYGYSILLL TVLVKL-ATF PLSKQQVESS IQMQAMQPRI KELQAMYAND P---------

YSYGFSIIVL TVLVKL-ATF PLSKKQVESS MQMQAMQPRI KELQAMYAND P---------

----GNAIDS ARYGQEMVLF MKEKNLNPLK NM-LVPLAQA PIFISFFMGL REMANT----

----GNAIES ARYAQEMMLF MREKGVNPLK NM-VVPLAQA PLFISFFMGL RQMANA----

KYEQILLLSA KEARKRQKEL FAKNGVQLWK NF-ILPAFQV PLWIMMSITM RDLSGWSW--

-DHKGHLDAI LLGRQGLKAV MKKYNTRPIQ TI-MGSVAYI PIFVLMAYSA RDMVRS----

-DHKGHLDAV LLGRQGMKAV MKKYNTRPVQ TI-MGSLAYI PIFVLMAYSA RDMVRS----

-DHKGHLDAI LLGRKGVKAV TKKYNTRPVQ TV-MGSVAYI PIFVLMAYSA RDMVRS----

TEKEARIMYN HSLKKQWNNL IVRENCHPAK TM-VLLWGQI PLWIVQSVAI RNLVSMLDE-

SEKQTQIVYR RSIKKQWQNL IVRDNCHPMK TM-IVLWGQI PLWIFQSVAL RNLVYMLDQ-

----MDSVTM AEGQKKMKNL FKEYGVTPFT PM-KGMFIQG PLFICFFLAI RNMAEK----

----MDPTAV SEGQKEMKKL FKEHGVSPFT PL-KGLFIQA PVFISFFLAI TNMAEK----

----MDPMAL AEGQKQMKKL FKEYGVSPLT PL-KGLFIQG PIFVSFFLAI SNMTEK----

----QDPEAM AEGQRRMQLL FKEHGVTPFT PL-KGLIIQG PIFISFFFAI RNMAEK----

---------S IDQFSHFLKE SRAIGCPSFL WFFPYLSVQL PCFFLLMASI RKMSLD----

---------Q MRAMEKRRLI MKENGVSTLA T-LF-PAVQL PLAYGFFQAL RKMANH----

----GDQAEF YKASSEMTFY QKKHDVKLFR PL-ILPLTQA PIFISFFIAL REMANL----

----GDHIEY YKASSEMALY QKKHGIKLYK PL-ILPVTQA PIFISFFIAL REMANL----

----GDQAEF YKATIEMTRY QKKHDIKLLR PL-ILPLTQA PVFISFFIAL REMANL----

SKRVARLTYL KNMRRLVSEL YVRDNCHPFK AT-ILVWIQF PMWIFMSVAL RNFSTGAAH-

SKRVARLTYL KNMRRLVSEL YVRDNCHPFK AT-VLVWVQL PMWVFISVAL RNLSTGATH-

SKRDARLTYL KNMRRLISEL YVRDNCHPFK AT-VLVWIQL PMWIFMSFAL RNLSTGAAH-

---------- DKIQKETSVL YEQAGVNPLA GC-LPTLATI PIFIGLFSSL TNVAN-----

---------- QLLNLETARL YQEAKVNPLT GC-LPVFVQL PVWIALYRAL MNLAA-----

----GDQQRI RALQQQVFRL LRENQISPLR SL-VNPLVQM PLFISFFLGL RKIAKI----

QLAS-NDDLL SSLTCLQRKA LQRYGTSRWR ALVWGRVVGL PAFVFAALRV RQLALAG---

---------- QKQQEELGKV MKEFG-NPLS GC-LPLLVQM PILFALFATL RGSPFADPYL

---------- QKQQEELGKV MKEFG-NPLS GC-LPLLVQM PILFALFATL RGSPFADPYL

---------- QKLREAQAKL YSELGVNPLG GC-LPLLIQM PVLFALFATL RGSPFAATYD

----NDHDGA AQAAANLNGL FKEHGVHPLK GL-LPLFVQA PVFISFFMAL RQMANL----

TDAEAENLIQ EQVRAQQKVL MDEIGWRRWK LV-LPALVQV PIFITVSLAL RRLQDSDL--

------PKSA KEGKAKMTAL FQKHGVSPFT PL-KGLLIQG PIFMSFFFAI RNMIDK----

---------F RDQFSLYQKK RRELGCPSFL WNWAYFSIQI A--------F HGTHTK----

-----GVRDV LDAIGELRGK APGTGAHPAW LV-AGPLAQI PFFLCAVMAV RRLAADG---

-----SIKDV LEAMKELRER S-GVGAHPAW LV-AGPLAQI PLFACAMMAV RRLASEG---

---------- ERMNKEINRV YEENGVNPLA GC-GPALLSF PVLAGLYRAF NNAGI-----

---------- ERMNKEINRV YEENGVNPLA GC-GPALLTF PVLAGLYRAF NNAGI-----

------PELA TAYYAEMQKV WKKYDVNPFK SF-APVLINA PVFISFYFAI SKMAAG----

------PELA NAYYKEMQKV WKKYDVNPVK SF-APILINA PVFISFFFAI SKMAQG----

---------- EVMNQKIAEF YQTNEINPLA GC-LPSIVQI PVFIGLYRAV LELAQ-----

---------- EVMNQKIAEV YQTNEVNPLA GC-IPSLVQI PVFIGLYRAV LNLAK-----

---------- EAQNRATAKL YEDAQQNPLA GC-FVALIQL PVFLGLYRGV RLLAM-----

------RQDQ LQFSGQMKAI FAKYKVKPFR AF-AGPVIQM PLFMGMFFGL RKMPSI----

------PEST KEFQTKYKAL MKKHDVNPFK SV-LTPLSQI PVFLGFFWGL QDISKY----

------EKST REFQTKYKAL MKKHGVNPFK SM-LTPLAQI PVFLGFFWGL QDISKY----

------PETA QEFQKKYKAL MKKHDVNPFK SM-LTPMAQI PVFLGFFWGL QDISKY----

QPEQITLLAV KETRKRQKKL FKKYNVPLWK NA-LLPMVQI PLWVTVSMGI RTLTETQ---

------LQQG QLVAMQRKKL LSSHGIKN-R WLA-APMLQI PIALGFFNAL RHMANY----

------QATQ QKVMAQTRAL WRKYDCNPMK GL-VVPLASF PFFMGMFFGL KKAPDY----

EAAQAQMSLL KTTVATLRGI FRLHKVNLLD IF-KSPLMQI PVFWYFAIDI RKIINGSD--

------MTEM QMVAHEKKKI LKKYGISQMK L-F-YPMAMF PLTIGIFLGI RRMCEIG---

---------Y IEQISLFRNE RRAIGCPSYL WFLAFLSVQI PCFLLWMTSI RRMCLD----

-PQIEM-LAM KERRKRRVKL YKEHGCEMWK SLFIGPLVQL PIWITMSLAV RAMCGWTV--

---------V LDQLKLFRKE RKDIGCPSFL WVPAYFSIQI SCFFLWITSI RRMSLD----

-PLRERLMLM RTNLGTLGGI YKLHGIHPMA VF-LSPLLQV PLFWYVSVDL RKIVNGLD--

---------- NMQNRAISKL FEDAQANPLA GC-VTSFAQI PIFLGLYRSV TRLAQ-----

---------- EKIQLETARL YKLAGINPLA GC-LPTLATI PVWIGLYRAL SNVAD-----

---------- ERIQLETARL YKLAGINPLA GC-LPTLATI PVWIGLYRAL SNVAN-----

---------- ERIQLETARL YKLAGINPLA GC-LPTLATI PVWIGLYRAL SNVAN-----

---------- ERIQLETARL YKLSDVDPLA GC-LPTLVTI PVWIGLYRAL SNVAN-----

---------- ERIQLETSRL YKQAGVNPLA GC-LPTLATI PVWIGLYQAL SNVAN-----

---------- ERIQLETARL YKQAGVNPLA GC-FPTLATI PVWIGLYQAL SNVAN-----

---------- ERIQLETSRL YRQAGVNPLA GC-FPTLATI PVWIGLYQAL SNVAN-----

---------- ERIQLETSRL YRQAGVNPLA GC-FPTLATI PVWIGLYQAL SNVAN-----

---------- ENLQLETARL YKEAGVNPLA GC-FPTLATI PVFIGLYNAL SNAAK-----

---------- ERLQLEQARL YREAGFNPLA GC-LPLFATL PVFIGLYRAL SNAAA-----

---------- ERLQMEQARL YKEAGFNPLA GC-LPVFATL PVFIGLYRAL SNAAS-----

---------- ---------- ---------- --------PV ESMRDGGLFW FTDLT-----

---------- ---------- ---------- --------PV ESMRDGGLFW FTDLT-----

---------- ---------- ---------- -------NLD PSLYEEGILW FQDLS-----

---------- ---------- ---------- -------GNF AGFDSGGLLF WKNLM-----

---------- ---------- ---------- -------GNF SGFDTGGLLF WKNLV-----

---------- ---------- ---------- -------GNF AGFDSGGLLF WKNLM-----

---------- ---------- ---------- -------AAY TELTLGGFGW IPNLT-----

---------- ---------- ---------- -------AVT TEMTIGGFGW IPNLT-----

---------- ---------- ---------- ---------V PSFQTGGALW FTDLT-----

---------- ---------- ---------- ---------V PSFKSGGAFW FVDLT-----

---------- ---------- ---------- ---------V PSFKSGGAYW FLDLT-----

---------- ---------- ---------- ---------V PSFKTGGTLW FTDLT-----

---------- ---------- ---------- --------GH PGFDSGGVLW FQNLS-----

---------- ---------- ---------- --------NV EGFSDQGYAW FQNLI-----

---------- ---------- ---------- --------PV PSLQTGGLLW FQDLT-----

---------- ---------- ---------- --------PV PSLQTGGLWW FQDLT-----

---------- ---------- ---------- --------PV PSLQTGGLWW FQDLT-----

---------- ---------- ---------- --------VQ EQLATDGILW FPDLT-----

---------- ---------- ---------- --------VQ EQLAAGGTLW FPDLT-----

---------- ---------- ---------- --------VQ EQLATGGILW FPDLT-----

---------- ---------- ---------- ---------- DGLLDQGFYF VPSLAGPTTM

---------- ---------- ---------- ---------- DNRLDQGFFW LPSLEGPVR-

---------- ---------- ---------- --------YP DELRNGGLGW FRDLS-----

---------- ---------- ---------- ---------D LGG-TLPGLW LKDLS-----

LPSDQIAAIE PKPFTSSKHS IFISETKHFP VIASLPSGTK LGVGDKAQIK LQTLTGESFT

LPSDQIAAIE PKPFTSSKHS IFISETKHFP VIASLQSGTK LGVGDKAQIK LQTLTGESFT

LPAEVAAEVV PTPYVSPSKN IFVTDSVHKP VVLVAPKGTK IAVGEQVQFR LQGPGGKPFE

---------- ---------- ---------- --------PI ESMKTGGLLW FTDLT-----

---------- ---------- ---------- --------YQ ADLASQGPLW TASLT-----

---------- ---------- ---------- ---------V PSMKGGGSLW FTDLT-----

---------- ---------- ---------- --------HY PG-------- ----------

---------- ---------- ---------- --------AN NGLVTGGALW FQDLT-----

---------- ---------- ---------- --------GS NGLISGGVFW FSDLT-----

---------- ---------- ---------- ---------- DGAFDEPWFF LPSLAGPTD-

---------- ---------- ---------- ---------- DGAFDEAWFF LPSLAGPTD-

---------- ---------- ---------- ---------V PSFETGGPSM YPDLS-----

---------- ---------- ---------- ---------V PSFESGGPSM YPDLS-----

---------- ---------- ---------- ---------- ANKLDESFLF LPNLEGPTYG

---------- ---------- ---------- ---------- ENALDEPFLF LPNLEGPTYG

---------- ---------- ---------- ---------- DGVLEEPFLW IPSLEGPVAP

---------- ---------- ---------- --------FP EELSTGGMYW FTDLT-----

---------- ---------- ---------- ---------F PEYAHEGIGW VPDLS-----

---------- ---------- ---------- ---------F PEYAHEGIGW VTDLS-----

---------- ---------- ---------- ---------F PEYAHEGIGW VTDLS-----

---------- ---------- ---------- --------LI ESF---YPSW FSALG-----

---------- ---------- ---------- --------PV DGFANQGVAW FTDLT-----

---------- ---------- ---------- --------FP DLLSNGGLFW FTDLT-----

---------- ---------- ---------- --------LA QQLVDSSFLW ITDLT-----

---------- ---------- ---------- --------GV QGLSTEGVLW FQNLA-----

---------- ---------- ---------- --------NH PGFDCGGALW FQNLT-----

---------- ---------- ---------- --------VV KSMGTEGALW FPDLL-----

---------- ---------- ---------- --------HH PGFDSGGALW FQNLT-----

---------- ---------- ---------- --------LA QQLVESSVAW VPDLT-----

---------- ---------- ---------- ---------- EGRLDEPFLW IPSLQGPVTA

---------- ---------- ---------- ---------- EGLLTEGFFW IPSLAGPTTV

---------- ---------- ---------- ---------- EGLLTEGFFW IPSLAGPTAI

---------- ---------- ---------- ---------- EGLLTEGFFW IPSLAGPTTI

---------- ---------- ---------- ---------- EGLLTEGFFW IPSLAGPTTI

---------- ---------- ---------- ---------- EGLFTEGFFW IPSLGGPTSI

---------- ---------- ---------- ---------- EGLLTEGFFW IPSLGGPTTI

---------- ---------- ---------- ---------- EGVLTEGFFW IPSLGGPTTI

---------- ---------- ---------- ---------- EGVLTEGFFW IPSLGGPTTI

---------- ---------- ---------- ---------- EGLLTEGFFW IPSLGGPTTI

---------- ---------- ---------- ---------- EHLLDDGFYW IPSLGGPTSI

---------- ---------- ---------- ---------- EGLLTDGFYW IPSLGGPTSI

---------- ---------- ---------I CD-------- -Q-------- ----------

---------- ---------- ---------M AD-------- -P-------- ----------

---------- ---------- ---------I AD-------- -P-------- ----------

---------- ---------- ---------E TD-------- -S-------- ----------

---------- ---------- ---------E TD-------- -S-------- ----------

---------- ---------- ---------E TD-------- -S-------- ----------

---------- ---------- ---------E LD-------- -H-------- ----------

---------- ---------- ---------V VD-------- -N-------- ----------

---------- ---------- ---------T PD-------- -S-------- ----------

---------- ---------- ---------T AD-------- -D-------- ----------

---------- ---------- ---------T PD-------- -S-------- ----------

---------- ---------- ---------T TD-------- -T-------- ----------

---------- ---------- ---------D LP-------- -GG------- ----------

---------- ---------- ---------E VD-------- -P-------- ----------

---------- ---------- ---------L SD-------- -P-------- ----------

---------- ---------- ---------V SD-------- -P-------- ----------

---------- ---------- ---------V SD-------- -P-------- ----------

---------- ---------- ---------A LD-------- -S-------- ----------

---------- ---------- ---------A VD-------- -S-------- ----------

---------- ---------- ---------A PD-------- -S-------- ----------

AMRQSGLGTS WLWPLGP--- ---------D GAP-PIG-WE DAA------- ----------

----QGQGLS WLFP-FQ--- ---------N GAP-PIG-WH DAI------- ----------

---------- ---------- ---------L PD-------- -P-------- ----------

---------- ---------- ---------E PD-------- -P-------- ----------

SRLSGVEGGT KFTPTWSVTK GDDLVKVSAD GTVQALA-EG DATVQGKIPG LAAQSGFLFI

SRLSGVEGGT KFTPTWSVTK GDDLVKVSAD GTIQALS-EG DATVQGKIPG LAAQSGFLFI

QLVAEAGGDP TLRPTWKITK GEERAQIQPD GTLLALQ-PG EVTVEVAIPG LASDTGFLFI

---------- ---------- ---------A AD-------- -P-------- ----------

---------- ---------- ---------E HD-------- ---------- ----------

---------- ---------- ---------T PD-------- -P-------- ----------

---------- ---------- ---------- ---------- ---------- ----------

---------- ---------- ---------V PAV-DVAMTA PMG------- ----------

---------- ---------- ---------L PAM-DIATSA PMG------- ----------

-----ARDLS WLLPLDA--- ---------D LAP-PIG-WD DAS------- ----------

-----ARDLS WLLPLDS--- ---------D YAP-PIG-WE EAS------- ----------

---------- ---------- ---------I AD-------- -P-------- ----------

---------- ---------- ---------M AD-------- -P-------- ----------

A--EPGSAAD WILKGWT--- ---------D GVP-SLG-WP DTI------- ----------

A--DPAHGSD WLFKNWV--- ---------D GVP-GLG-WE DTI------- ----------

---PNFQGLD WLVQGWV--- ---------N GAP-ALG-WE TTL------- ----------

---------- ---------- ---------A SD-------- -P-------- ----------

---------- ---------- ---------V AD-------- -P-------- ----------

---------- ---------- ---------A SD-------- -P-------- ----------

---------- ---------- ---------A AD-------- -P-------- ----------

---------- ---------- ---------L SS-------- -PL------- ----------

---------- ---------- ---------Q AD-------- -P-------- ----------

---------- ---------- ---------Q AD-------- -P-------- ----------

---------- ---------- ---------E PD-------- -P-------- ----------

---------- ---------- ---------A PD-------- -P-------- ----------

---------- ---------- ---------E LP-------- -HG------- ----------

---------- ---------- ---------M MD-------- -H-------- ----------

---------- ---------- ---------E IP-------- -NG------- ----------

---------- ---------- ---------E AD-------- -P-------- ----------

E--TNYRGTE WLTQGWV--- ---------D GVP-PLS-WE TTL------- ----------

AARQNGSGIS WLFPF-I--- ---------E GHP-PLG-WP DTL------- ----------

ASRQNGSGIS WLFPF-V--- ---------D GHP-PLG-WS DTV------- ----------

AERQNGSGIS WLFPF-V--- ---------D GQP-PLG-WS DTV------- ----------

AARQSGQGIS WLFPF-T--- ---------D GHP-PLG-WS DTL------- ----------

AARQSGSGIS WLFPF-V--- ---------D GHP-PLG-WY DTV------- ----------

AARQSGAGIS WLLPF-V--- ---------D GHP-PLG-WH DTI------- ----------

AARQSGSGIS WLFPF-V--- ---------D GHP-PLG-WH DTA------- ----------

AARQSGSGIS WLFPF-V--- ---------D GHP-PLG-WN DTA------- ----------

-----GGGLE WLVPF-E--- ---------N GAP-PVG-WA NAA------- ----------

AARNDGNGFA WLWPF-V--- ---------D GHP-PLG-WY ETG------- ----------

ASRNAGSGFA WLWPF-V--- ---------D GHP-PLG-WH DTT------- ----------

---------- --FYAL---- -PIITSLTLF ATIELGTDSA RMS-AANMQT --AKYILRAL

---------- --FYLL---- -PLITSATLY LTIEIGTDSA RLS-AANMNT --MKYVLRAL

---------- --MHVF---- -PVILGITAL CNIEWTLKTL ELR-PTLTDA --FGNLTKMS

---------- --TYVL---- -PILAATSTY GNLELSVRNK S----GFWTT --LLQGGQYI

---------- --TFIL---- -PILAATSTY GSLELSMRTK S----GLWTD --LLQIGQYG

---------- --TYIL---- -PIIAAASTY GNLELSIRKK S----SFWTQ --VLQFGQYG

---------- --SLIF---- -PVALGVINL SIIEIQAASR TKLPSKLQTI --FTNLFRGL

---------- --SYIL---- -PVALGLINL AIIEVQAMSR TRPSTRLQNI --ANNVFRGL

---------- --LYIL---- -PVITGLTFL ITVECNAQEG MEG-NPMAGT --VKTVCRVF

---------- --LYIF---- -PVLTALTFL ITVECNTQEG MEG-NPAAGT --MKNVSRAL

---------- --LYIL---- -PILTGLTFW ITVECNMQEG LEG-NPIAAT --MKKVSRVF

---------- --TYIL---- -PLLTAVTFL IMVESNMQEG LEG-NPVAGT --MKKFSRII

---------- -SFPVF---- -PILIATFHY INIQISFDTS TITG-LLMRY --YKLYLEIL

---------- --YLGL---- -QAISAAAII AVVRVGGETG QH---AMAAG --MKKVMTVV

---------- --TYIL---- -PLVVTATMW GVLELGAETG MQ-S-SDLQW --MRNLIRVM

---------- --IYIL---- -PLAVTATMW AVLELGAETG VQ-S-SDLQW --MRNVIRMM

---------- --IYVL---- -PLVVTATMW CVLELGAETG VQ-S-NDLQF --MRNIIRVM

---------- --TWIL---- -PISVGVINL LIVEIFALQK IG-MSRFQTY --ITYFVRAV

---------- --TWIL---- -PVSVGVVNL LIVEIFALQK IG-TSRFQMH --VTNFVRAV

---------- --TWIL---- -PISVGVINL LIVEICALQK IG-MSRFQTY --ITYFVRAM

---------- -AYLTL---- -PLLLVAVQY ASSSVTSPPI DPK-DENANT --QRALLVFL

---------- -AYLVL---- -PCLLVVSQS ISQKILQPPV --Q-DPQQQQ --ANAILRFL

---------- --FYGL---- -PALTSATML FMIQMGTETG GA---QLPPF --ALNLMRIF

---------- --FYLL---- -PLCNMVLML RNLEYSFPLM PFPYWLQSQS --LKVLLQGA

KALGQVGFYV DGEINWDIAI LVGGFGLTLL VSQIL----- SGR-GPANPQ QSTANKITPV

KALGQVGFYV DGEINWDIAI LVGGFGLTLL VSQIL----- SGR-GPANPQ QSTANKITPV

DKLGRVGAF- DPTIHWDIIG MIVLFGVSIY LNQSLTNA-- ----GQEDPS QSSMARITPV

---------- --YYVL---- -PVIASATML ATIEFGSEGV K----QNNVM --MKNVFRGL

---------- --ILLL---- -PLVLGITNW FTLELSTIKR PVSPESREDR --LGTALRYL

---------- --LYIL---- -PVLTALIFL VTVELNLQEG MEG-NPMARK --MKNFSRGM

---------- ----IF---- -G-------- ---------- -----VLAKY --YRVYLEIL

---------- -PGGIL---- -PVLTAAALF ANVNANFASA AAQ-SRGMTI --VKLVLEWM

---------- -PGAVL---- -PIVTAGALF ANVNANFAAA AQQ-SRGMTI --VKLCLEWM

---------- -LYLLF---- -PIMTTLSQF VSMEVLKPEE DEK-TKEMQN --QSVLLKLL

---------- -LYLIF---- -PVLTTISQF VSMEVLKPEE TEK-TDEMKN --QSVLLKLL

---------- --TYSL---- -PILSSLTFL ASVELGTVEG MQ-T-SQSAQ --MKWFLRAL

---------- --TYSL---- -PILSSLTFL ASVELGAVEG MQ-T-SQSAQ --MKWFLRAL

---------- -AFLIL---- -PVFLVISQY LSMELMQPKT --D-DPAQQQ --SNAILKVL

---------- -AFLSI---- -PVFLTISQV ISMNMMQPKT --D-DPQQQQ --ANVILKVL

---------- -AFLIM---- -PVILVVLQS VTMQVLQPPV DEEERETLER --SQTILKFL

---------- --LYIL---- -PFTSALSFL ALIELGKEQM VAQNAQSGHL --MVNFFRVM

---------- --TLAL---- -PVISSALMA ASVELGGDAM GG---DMQRN --LKFGMRCF

---------- --TMAL---- -PIISSALMV ASVELGGEAM GD---EMKGK --MKFGMRCF

---------- --TMAL---- -PCISSALMV ASVELGGEAM TG---EMKDK --MKFGMRCF

---------- -VPLLA---- -PILVGTLAV LNVELNGRLM FSNSTRVQEA MSILNVSRLG

---------- --YLGL---- -QVITAAVFI SFTRLGGETG AQ---QFSSP --MKRLFTIL

---------- --LMIL---- -PVLSAGTFL VMTELTKDQM MSSDPVRGRN --MVNAMRAL

---------- --WYGL---- -PIITGLLLY WNVETAVGKK ALSQSRMALF --LKDAFQSL

---------- --YLGL---- -QVITAAMYM ASIRLGSETG TN---NLSPG --MKKILQWA

---------- -VLPIF---- -PFLIAGLHG VNVH------ ---------Y --YRKYLNFM

---------- --SGVL---- -PAAVGIITL LNVELTTKAQ AQAGPKLPKM --MANFARVG

---------- -LYPLF---- -PFLIAGLHY TNTQVCT--- ---------- ----------

---------- --WFGL---- -PVLAGLVMY ANVEVAIGRR SLSKADTGVL --LKDVFQSL

---------- -AFCVM---- -PVLLVLGQS FTMNVLQQPD DDETKKQMES --TKTILKFL

---------- -AYLVL---- -PLLLVFSQY LSIQIMQS-S QSN-DPAMKS --SQAVTKLL

---------- -AYLVL---- -PVMLVVSQY ISVQIMQS-S QSD-DPNVKN --SQAITKFL

---------- -AYLVL---- -PAMLVVLQY MSVQIMQS-S QSD-DPNVKN --SQAIMKFL

---------- -AYLVL---- -PVLLVISQY VSSQVMQP-P QNN-DPSQQG --AQAVVKFL

---------- -AYLVL---- -PVLLIASQY VSMEIMKP-P QTD-DPAQKN --TLLVFKFL

---------- -CYLVL---- -PVLLVASQF VSMEIMKP-P QTD-DPSQKN --TLLVLKFL

---------- -AYLVL---- -PVLLIASQY VSMEIMKP-P QTD-DPTQKN --TLLVFKFL

---------- -AYLVL---- -PVLLVVSQY VSMEIMKP-P QTD-DPTQKN --TLLVFKFL

---------- -AYLVM---- -PVLLVASQY ASQKIISS-Q NNQ-DPSQQQ --AQAILKFL

---------- -CYLVL---- -PVLLVVSQF VSQTIISPQP KTD-DPAQQQ --SQAILKFL

---------- -AYLVL---- -PVLLVASQY VSQQIVSPQP KTD-DPAQQQ --SQAILKFL

PLFIFPFTIN FPGAILCYWA CSNFFSLVQV GFLRIPKVRD FFKIDRIVTH KPETLP---K

PIVIFPFTMN FPAAILTYWA CSNFISLGQV AVLRIPSVRE YFKIEKMLTH APSALP---P

IVFMMAISLH APAALTIYWI SSQLYSLLQN VMMDLMLPIS FTPKKRININ ----------

TIFAVPVLVN LPQGVFFYWL GASCSSMAQT IAMNNNNFRR RIGLKPRITE TKPPAA---A

SLLAIPWLAT LPQGIFFYWL GASWSSMAQI AAMDNNNFRR RIGLKPRITQ TESPAA---A

TIFAIPLMAN LPQGVFFYWL GASWSSMAQT IAMDNNNFRR RIGLKPRIAH TESPAA---A

SILMVPIAAS VPSCLCLYWV TSSAYGLGQN LLLLSPRVRR TVGIPAVPSE LTHPYQ---H

SVVMVPVACT VPSALCVYWV ASSSFGLAQN LLILSPEVRR SVGIPKTQTE LSEPYD---L

ALLTVPMTMS FPQAIFCYWI TSNLFSLMYG LVIKRPQVKK MLRIPDLPPP PPGQQP----

AAASVPLTMS FPKAIFCYWI TSNLFSLTYG LVLKAPGVKE FLGVPKVPVA PPTTAA---K

AVASVPLTMG FPNAIFCYWV TSNLFSLFYG LTLKAPGVKK FLGLPEIPVA PASTTP----

AFLSIPVLIG IEKALFCYWL TSNLFTLVYG LTLRRPDVRK LLNLPDVVNS STRQP-----

SVPLFFVGYA IPQGSLVYWV TNSSVNIFQQ LSLKHPTVGA KLGLLSQGAT PGGHSM---E

PIASIFITKG FASAIILYFA VNSIFSLIQS SLFKSSWFRK IAGMPPKLSL AEMQANNPKA

PLAVLPITIH FPTAVFMYWL SSNMFSLGQV ACLRIPAVRT ILKIPQRVVH DSNKLLP---

PLITLPITMH FPTAVFMYWL SSNLFSLVQV SCLRIPAVRT VLKIPQRVVH DLDKLPP---

PLVVLPVTIH FPSAVFMYWL SSNVFSLCQV ACLRIPAVRT VLKIPQRVVH DPDKLPP---

SVLMIPVAAT VPSSIVLYWL CSSLMGLSQN LLLRSPRFRQ LCRIPLTKSD SDSPYK---D

SVLMIPVAAT VPSALVLYWL CSSLMGLAQN LLLRSPGFRQ LCRIPPSKSD SETPYR---D

SVLMIPIAAT VPSSIVLYWL CSSFVGLSQN LLLRSPGFRQ LCRIPSTKSD SETPYK---D

PLMVGWFSLN VPAGLSLYYL ANTVLSSAIQ IYLKKLGGAN VVMNELGPVT KPGSGR---R

PFMVGWFSLN VPSGLGLYWV TNNIVSTIQT IGIKRYLASK QPERVGAPRT ---LDGAVDD

ALILLPLTAQ FPAILFCYWL PNNLFSMLQA FALRHQRVRR WLGCPPLGAR SAAATA---G

VLVITPGVVN LPSAVLLFWL TNSCIQTALT SAWWRQRWHR WIQQTALQRE LDKFDQA---

MITGMFLFFP LPAGVLLYMV IANIFQAGQT FLLSREALPE NLQKILNDQL SKPALATEAI

MITGMFLFFP LPAGVLLYMV IANIFQAGQT FLLSREALPE NLQKILNEQQ SKPALATEAI

LFSAMFLFFP LPAGVLLYIL VSNIFQTVQT FLLSREPLPE NLQQLVEEER RRAAQASTGE

SIVLLPVTIN LPTAIFVYWC TANMFSLSQM LMLKIPGLKK SLGIPEQIQH ----------

AIAMIPVAAA VPSGLSLYWA SSSVLTLTQN LMLRHPSVRQ RVGLPAVPSE LPRPLT---S

AVLTVPFTMS FAKGIFCYWI TSNLFTLTYG FVIRRPAVRK FCNLPALEAQ SASAKK---Q

TIPLFLIGYV IPQGSLVYWT TNGLITVAQQ LSLKNDAVKK VLGLPDTRAH QKFPRV---G

TLPMLVIGMQ LPQAVHCYWL TSSAWALAQN RALSTTFARE ALGINALAKV TREIAA---S

TLPMLLIGLQ LPQAVHCYWI TSSAYAYAQN RALSTAYARE ALGLNELAKT TREIVR---E

PFFIGYISLT VPAGLALYWF WNNVFTTGIQ VYLRNGGA-- ---------- ----------

PLFIGYISLT VPAGLALYWF WNNVFTTGIQ VFLRNGGAKA TVE------- ----------

AVAMVPLTAS FPQGVFVYWI TSNMFSGVQT SITRTKGFKA AMGIPDVSAV ADAPAD---A

AVAMVPLTAS FPQGVFVYWI TSNIFSGFQT SITRTKAFKS TMGIPDVSAV AEAPAE---A

PIMIGWFSLS VPAALSVYWV INNIITTGTS LIIRNSMSVE TVTPSGTATA SAPPRA---N

PFMIGWFALN VPAALGVYWV VNNIVTTATT LYVR------ ---------- ----------

PLLIGFFALQ VPAGLTIYWF TSNIFTLTQS LAVRAYFSAN PPQIELPDYW DTALKN---Q

SIGMVPVCVN FEAAMLCYWT SNNFMTLTQT AILKAPAARS YFGIWDAPKP VPGQEP---E

ALMMVPLTMN FQSGIFVYWV TSNMFTLTQT ALMRLNVVKR ALNIPVTEVQ RLEAST---I

ALMMIPLTMN FQSGIFVYWV SSNMYTLVQT ALMRLNFVKR ALKIPVSEVQ RLEASS---I

ALMMVPLTMN FQSGIFVYWV TSNMYTLTQT ALLRVNFIKR ALKIPVTEVQ RLEAAS---I

CVVMLAMSSQ APFLLSLYWI SSQLFSLVQN IILNWIYPYQ R--------- ----------

PIISIPATMN LSSAVVLYFA FNGAFSVLQT MILRNKWVRS KLKITEVAKP RTPIAG---A

AIVMVPLTAY FNSAVLCYWV TNNSFTMGQS LFFKLEPVKK MFGIWDPPKP VPGQET---G

AVFMPCFMAQ QPSGVQIYLT TSMLFSLLQS MAMRDDAVRE YIGLPALNAK PVDMGD---S

PWISVPFLMK MPAALLLHFF VNGILMLIQG VALRNPFFRK KLGIHEIVPL PAAAPG---A

MLPLFFIGYC IPQGSLVYWV TNSSLTAIQQ VSLKLPVVRA KLGLLDKDFP KAPALS---A

AIALFSIAAQ TPTAVCLYWI SSSGFSLIQN VLLNKYMPLR EEPPMFTAAN ----------

--VWFFVGS- ---------- ---------- ---------- ---------- ----------

AVFMPCFTSQ MPAGVQIYLV TSFLFTMGQS AALRTEAFRA AVGLPSLATA PPPEAK---Y

PLMIGFFSLQ VPAGLTIYWF TSNLFTVSQS LIIRGYYAAN PPEVKLPDY- ----------

PLMIGYFALS VPSGLSLYWL TNNILSTAQQ VWLQKYGGAK NPVEKFTNLV TKEDKT---Q

PLMIGYFSLS VPSGLSLYWL TNNILSTAQQ VWLQKSGGAK NPMMKSSDDI V---------

PLMIGYFSLS VPSGLSLYWL TNNILSTTQQ VWLQKLGGAK ---------- ----------

PLLIGYFALS VPSGLSLYWL TNNILSTAQQ VWLQKLGGAK NPVKEYIDKL AKEEST---N

PLMIGYFALS VPSGLSIYWL TNNVLSTAQQ VYLRKLGGAK PNMDENASKI ISAGRA---K

PFMIGWFSLS VPSGLSIYWF TNNILSTAQQ VWLRKLGGAK PVVNQGGSGI ITAGRA---K

PIMIGYFSLS VPSGLSIYWF TNNVLSTAQQ VWLRKLGGAK PVVNENASGI ITAGRA---K

PLMIGYFSLS VPSGLSIYWF TNNVLSTAQQ VWLRQLGGAK PVVNENASGI ITAGRA---K

PLMIGWFSLN VPSGLTLYWF VNNLLSTGQQ LYLKATVKVN -IPEAIKAPA TAGSST---P

PFMIGFFSLN VPAGLTLYWF FNNIITTAQT VVLRKITKPI EVPTGAGAGS SPASAE---P

PLMIGFFSLN VPAGLTLYWF ANNIITTAQT LILRKTTTAP EPPVAKSGAA G---------

GFTD---

GFVGEQG

-------

VAEM---

AAEM---

AAEM---

IRS----

LWLA---

SFDLSKK

SFDLK--

SFDLNKR

SPPLKKK

ISESSHF

NQSIQRH

GFLKPLG

GFLEPLG

GFLKPLG

LFA---A

LSA---A

IFA---A

NGAAGGA

------R

H---TNR

LDQASKH

GGSDRSK

GGSDR-K

AKEVFEP

-------

LRRHGNP

MFNLMRP

MMQEKEH

L---GGE

L---HSD

-------

-------

A---LSR

A---LGR

-------

-------

A---VSA

T---KAA

T---TSP

T---QTP

T---TSP

-------

T---ENM

L---EK-

VQEFPKK

G---VRK

L---SSS

EALPKEH

-------

QFIAKKG

-WGASKT

QISSTTR

-------

-------

LGPC-NK

RSAE---

RTAS---

RSSG---

RSAG---

IKKA---

ISSG---

-------

49 515

Aga_Oxa1 ---------- ------GLGG WTPVGIVQNC MEFLH----- IGLD---LPW WGCIAIGTVC

Dme_Oxa1 M-SRV----- ---PKTGLGG WSPVGMVQNC LEFLH----- CTWD---IPW WGTIAIGTLA

Aga_Oxa2 ---------- ---FWQTLSQ SAPVAYVQQG MINLH----- DLTG---LPW WATVILTTVG

Dme_Oxa2 M-GTTIN--- AGVYWQTLSN STPVAYMQDV LIKIH----- DYSG---LPW WASIVLSTFL

Cal_Oxa1 M-TSG----- ----LAQGW- -GPTSLIERL LEVTH----- VYTG---LPW WGTIVVATIA

Cal_Oxa2 M--PGR---- ----NFSIDH NAIINTMTSS FQTVH----- EFSG---LPW WALIPLTTFT

Cfa_Oxa1 ---------M APPV--GLGS YTPVGLIQNL LEFMH----- VNLG---LPW WGAIAACTVL

Hsa_Oxa1 M--------M APPV--GLGS YTPVGLIQNL LEFMH----- VDLG---LPW WGAIAACTVF

Mmu_Oxa1 ---------M APPV--GLGS YTPVGLIQNL LEYIH----- VDLG---LPW WGAIATCTVL

Cfa_Oxa2 M-GPGW---- ----YEALAA SAPVRGAEEM LLGLH----- AAAG---LPW WACIGLGTVA

Mmu_Oxa2 M-SPGW---- ----YEALAA SAPVRTAEEV LLGAQ----- EATG---LPW WSNIILSTVA

Hsa_Oxa2 M-HANW---- ----YEALAA SSPVRVAEEV LLGVH----- AATG---LPW WGSILLSTVA

CreAlb3.1 M-S------- -SAIRAGGWV APVADALEQV LYALQEGLDK LHVP---YSY GYSIILLTLI

Cme_Oxa1 L-RLQ----- ----QKAGLW NGFVHLIETI ITGTGDTLAA LGVP---GSY GFAIIFLTII

Pma1_YidC M--------- ---------I GYISD---NI LLPILDFFY- -GLV---PSY GLAIVALTVV

Pma2_YidC M--------- ---------I GYISD---NI LLPILDFFY- -GLV---PSY GLAIVALTVV

Syn_YidC M-FG------ ---------V GFLSN---NV MLPILDFFY- -GIV---PSY GLAIIFLTLV

Lbr_OxaI ---------- QLEEDFWDWA VSFLQPVEKQ VEIMRSLRHD GFMSLDLGGW GHVFFFYGLC

Lin_OxaI M-APTSSIAC QLEEDFWDWA VSFLQPVEKQ VEIMRSLRHD GFMSFDLGGW GHVFFFYGLC

Lma_OxaI --APTSSIAC NLEEDFWDWA VSFLQPVEKQ VEIMRSLRHD GFMSFDLGGW GHVFFFYGLC

Tbr_OxaI ---------M EIEEDFWDWI VGFLQPVEKQ VEIMRHLRNE GVFGFDFGGW GNVFFFYGIF

Tcr_OxaI-1 M-AAAPLTVF QIEDDFWDWI VSFMQPVEKQ LEVMRSLRHS GFLGFDFGTW GNVFFFYGIV

Tcr_OxaI-2 M-AAAPLTFF QIEDDFWDWI VSFMQPVEKQ LEVMRSLRHS GFLGFDFGTW GNVFFFYGIV

Lbr_OxaII M-GSTA---- ----TTAHFA SADPSLFTQV FLSCQ----- QALS---IEA GTLLLLTGAV

Lin_OxaII M-GSAV---- ----AKAHIA SADPSLFTQM FLSCQ----- QALG---MEA GTLLLLAGAL

Lma_OxaII --ESAA---- ----ARAHIA SADPSLFTQL FISCQ----- QALG---MEA GTLLFLAGAL

OtaAlb3.1 ---------- ----RMEGWL APVSNALEDL LFTIKGQLLD LGVP---YPT GNAIIIVTIL

OluAlb3.1 ---------- ----RMEGWL APVSDALEDL LFAIQGQLQG LGVP---YST GNAIIIVTIL

Ptr_Alb3-1 M-RSFG---- ----SDSGWF GFLTIPIKLL LQAIHSLLLT VGLN---TSW GVAIVLLTIL

Tps_Alb3-1 ---------- ----AQNGWF GFLTLPIEGL LKLIHGGLDS MGMS---SAW GISIIAMTVV

Ptr_Alb3-2 C-RRQV---- ----DDGGWW GAYIQLFKTT LNAVHSTIQG -PLQ---NSW GVSIAIFTTI

Sce_Oxa2 ---------- --------MS FSLFQSVADT FLTVH----- EASH---IPW IVLVPLTTMT

Sce_Oxa1 M-LPSL---- ----A-QTW- YWPSDIIQHV LEAVH----- VYSG---LPW WGTIAATTIL

Tbr_OxaII M--------- -----VGIAP SQEPAFITNM FVSFQ----- EGLN---LGA PEAILLLGVL

Tcr_OxaII ---------- -----MNLLP LPDASPFADL FVTCQ----- ETLG---LEP AIAILLFGAL

Tps_Alb3-2 -----M---- ----DENSWW ESYIQIYKNG LAFVHDNIVD EPLR---KTW GVSIFLFTAG

Yli_Oxa1 M-ERDI---- ----TGSLWS LWPSDIYLNL LEHVH----- VYTG---LPW WAAIASTTVI

Yli_Oxa2 ---------- --------MT VDIVRPVETA LNAIH----- DFSG---LPW WAVIPLVTLT

AthAlb3.b MSS------- -SRAQSNDWF SGIANYMETI LKVLKDGLST VHVP---YSY GFAIILLTVL

PAlb3.b-1 ---------- ---VQNSDWL SGITSCLEST LKVLKDGLSA LHVP---YAY GFAIILLTVL

PAlb3.b-2 ---------- ---VQNSDWL SGITYGLEST LKVLKDGLSA VHLP---YAY GFAIILLTVL

OsaAlb3.b MRR------- -VVVAAGDWL SGITNSMETV LKVLKDGLSA LHVP---YPY GFAIILLTVL

AthAlb3.a M-F------- -SLNKSGGWF GFISDAMELV LKILKDGLSA VHVP---YAY GFAIILLTII

OsaAlb3.a M-S------- -ACRKNGGWF GFISEALEVV LKVLKDGLSA VHVP---YSY GFAIILLTVI

PAlb3.a-1 M-L------- -HDNKNGGWF GFISDGMEFV LKVLKDGLSS VHVP---YAY GFAIILLTIA

PAlb3.a-2 M-L------- -HDNKSGGWF GFISDGMEFV LKVLKDGLSA VHVP---YAY GFAIILLTVF

CreAlb3.2 M-S------- -QPVAEGGPI DVLAQFFEFV LQTLDEGLES AKIP---YSY GFAIIALTVL

OtaAlb3.2 M-A------- -HAAQKGGWL GPITDALEGA LRGIDGVL-D GKVP---YSY GYSILLLTVL

OluAlb3.2 ---------- ----QKGGWL GPITDGLESA LEGIDSVL-D GRVP---YSY GFSIIVLTVL

VR-TLLFPLV IASQRNAAKM NNYMPQLQ-- --VLQMKMTE ARQAGNAIDS A---------

VR-TIIFPLV ILAQRNSAKM NNNMPQMQ-- --MLQLKMTE ARQSGNAIES A---------

LRTLVTLPLA VYQNKILARL EQISLEMP-- --ELIKELKA ETAYAMKFNW TEKEARIMYN

FRSVVTLPLT IYQHKITARI EKIALEMP-- --AIVEELKK EAAMAKHFKW SEKQTQIVYR

VR-LVLFPLY VRASSNATKM SKIKPQID-- --ELLQQIKT GDTDQ----- --------M-

LRSVWTLPLA ILQRKRIQKQ SQLRPLVS-- --AMNPILKL NLARRVQAKM KYEQILLLSA

AR-CLVFPLI VKGQREAAKI HNHLPEIQ-- --KFSTRIRE AKLAGDQAEF YK--------

AR-CLIFPLI VTGQREAARI HNHLPEIQ-- --KFSSRIRE AKLAGDHIEY YK--------

AR-CLVFPLI VKGQREAAKI HNHMPEMQ-- --KFSARIRE AKLAGDQAEF YK--------

LRGAVTLPLA AYQHYILAKV ENLQPEIK-- --NIARHLNQ EVAVRANLKW SKRVARLTYL

LRGAVTLPLA AYQHYILAKV ENLQPEIK-- --DIAKRLNQ EVAVCARFGW SKRVARLTYL

LRGAVTLPLA AYQHYILAKV ENLQPEIK-- --TIARHLNQ EVAVRANLGW SKRDARLTYL

VK-LLTYPLT KQQVESAMAV QALKPRID-- --LIKDRFGE DKDK------ ----------

VK-AITFPLN YKQMKSTMAM QALAPKVR-- --ELQARYRD NPQL------ ----------

IR-LALFPLS AGSIRSARRM RIAQPAMKKR QDEIKSRYAK DPQK------ ----------

IR-LALFPLS AGSIRSARRM RIAQPAMKKR QDEIKSRYAK DPQK------ ----------

IR-FALYPLN VGSIRNMRRM KVINPLMQRR MREIQEKYRD DPQK------ ----------

MR-VLTLIPS LYSHRNSLRM AHIGPQVS-- --EITNSQNK VKNDRTL--- SSAEKRVI-K

MR-VLTLIPS LYSHRNSLRM AHIGPQIS-- --EITNNQNK AKNDRTL--- SSAEKRVI-K

MR-VLTLIPS LYSHRNSLRM AHIGPQIS-- --EITNNQNK AKNDRTL--- SSAEKRVI-K

MR-LCTLVPS LLSHRNALRL SHINPQLS-- --EIATCQNR AKSDRSL--- STAEKRVI-K

MR-LCTLVPS LYSHRNALRM SYIGPQIS-- --EITNNQNR VKNDRTL--- SSAEKRVI-K

MR-LCTLVPS LYSHRNALRM SYIGPQIS-- --EITNNQNR VKNDRTL--- STAEKRVI-K

TR-LCTLFFS LYGERAAERM RLALPELK-- --KPQEDFNR VYFDFA---- SAMEVQMA-A

TR-LCTLFFS LYGERAGERM RLALPELK-- --KPQEDFNR VYFDLA---- SAMEVQIA-A

TR-LCTLFFS LYGERAGERM RLALPELT-- --KPQEDFNR VYFDLA---- SAMEVQIA-A

VK-MVTYPLT KDQVVSSLNM KNLQPQIA-- --AIREKYED DQER------ ----------

VK-FVTYPLT RDQVVSSLNM KNLQPQIA-- --AIREKYED DQER------ ----------

IK-VVTFPLT KTQLESTNKM QAMQPAIK-- --ELQAKYQS NPEV------ ----------

IK-ALTFPLT KSQLESTNKM QALQPTIK-- --SLQAKYQS NPEV------ ----------

VR-TLLVPLS IEQSKSAEYI KSLKPYVA-- --DIKAKYKN NQEA------ ----------

LRTLVTLPFS IWQRRRILKQ QELRKLVQ-- --PITPIIKL RLAAVTNKSL TPEQITLLAV

IR-CLMFPLY VKSSDTVARN SHIKPELD-- --ALNNKLMS TTDQQ----- ----GQ--L-

CR-VATLGFS LYGERASERM RKAICKLK-- --TPHEAYQR VYHEGA---- TSLDIQLA-A

SR-LCTLCFS LYGERASERM RNAMRRLK-- --APHEAFQR VYYEGS---- AALDIQLA-A

VR-ALLVPFS IQQSKSSEYM KALKPYQQ-- --KIKEKYTD -KNM------ ----------

VR-VLLFPLF VQAANEQGKM SEVKPELN-- --VIDEKLKS AANTE----- ----MQ--M-

LRSTVTLPIA ISTRLRAQKQ HELRPLIS-- --ALGPILRA KLAFNANAAL TAPQIEMLAM

VK-AATFPLT KKQVESAMAM KSLTPQIK-- --AIQERYAG DQEK------ ----------

VK-AATFPLS KKQVESAMAM RSLQPQIK-- --AVQQLYAG DQER------ ----------

VK-AATFPLS KKQVESAMAM RSLQPQIK-- --AIQQRYAG DQER------ ----------

VK-AATFPLT KKQVESAIAM RSLQPQVK-- --AIQERYAG DQER------ ----------

VK-AATYPLT KQQVESTLAM QNLQPKIK-- --AIQQRYAG NQER------ ----------

VK-AATLPLT KQQVESTLAM QNLQPQIK-- --AIQQRYAG NQER------ ----------

VK-VATLPLT KKQVESTLAM QNLQPKIK-- --AIQQRYAG NQER------ ----------

VK-VATLPLT KKQVESTLAM QNLQPKIK-- --AIQQRYAG NQER------ ----------

VK-VATFPLT QKQVESTLSL QALQPRVK-- --ELQAKYAD DPEN------ ----------

VK-LATFPLS KQQVESSIQM QAMQPRIK-- --ELQAMYAN DPER------ ----------

VK-LATFPLS KKQVESSMQM QAMQPRIK-- --ELQAMYAN DPER------ ----------

-RYGQEMVLF MKEKNLNPLK NMLVPLAQAP IFISFFMGLR EMAN-P---- ----------

-RYAQEMMLF MREKGVNPLK NMVVPLAQAP LFISFFMGLR QMAN-P---- ----------

HSLKKQWNNL IVRENCHPAK TMVLLWGQIP LWIVQSVAIR NLVSML---- ----------

RSIKKQWQNL IVRDNCHPMK TMIVLWGQIP LWIFQSVALR NLVYML---- ----------

--RAMEKRRI MKENGVSTLA -TLFPAVQLP LAYGFFQALR KMANHN---- ----------

KEARKRQKEL FAKNGVQLWK NFILPAFQVP LWIMMSITMR DLSGWS---- ----------

--ASSEMTFY QKKHDVKLFR PLILPLTQAP IFISFFIALR EMANLP---- ----------

--ASSEMALY QKKHGIKLYK PLILPVTQAP IFISFFIALR EMANLP---- ----------

--ATIEMTRY QKKHDIKLLR PLILPLTQAP VFISFFIALR EMANLP---- ----------

KNMRRLVSEL YVRDNCHPFK ATILVWIQFP MWIFMSVALR NFSTGA---- ----------

KNMRRLVSEL YVRDNCHPFK ATVLVWVQLP MWVFISVALR NLSTGA---- ----------

KNMRRLISEL YVRDNCHPFK ATVLVWIQLP MWIFMSFALR NLSTGA---- ----------

--IQKETSVL YEQAGVNPLA GCLPTLATIP IFIGLFSSLT NVAN------ ----------

--LNLETARL YQEAKVNPLT GCLPVFVQLP VWIALYRALM NLAA------ ----------

--QQEELGKV MKEFG-NPLS GCLPLLVQMP ILFALFATLR GSPFADPYLN LVLPSDQIAA

--QQEELGKV MKEFG-NPLS GCLPLLVQMP ILFALFATLR GSPFADPYLN LVLPSDQIAA

--LREAQAKL YSELGVNPLG GCLPLLIQMP VLFALFATLR GSPFAATYDN LILPAEVAAE

DGYNRMKYAL CKRHRCAQWK -SFLTVLTAP ITMSAFLSIR RLAMY----- ----------

DGYNRMKYAL CKKHNCSQWK -SFLTTFTTP ITMSAFLSIR RLAMY----- ----------

DGYNRMKYVL CKKHNCSQWK -SFLTTLTMP ITMSAFLSIR RLAMY----- ----------

EGYNRMKYAL MKKHHCAQWK -NFLSMITAP VTLSAFISVR RLAVY----- ----------

DGYNRMKYAL MKKHGCAQWK -SFFTMLTAP ITMSAFFSIR RLAVY----- ----------

DGYNRMKYAL MKKHGCAQWK -SFFTMLTAP ITMSAFFSIR RLAVY----- ----------

SVLKSHRRTV FGKYHTSSLK -CIASLGMAP VIITGLYQVS ALCENV---- ----------

SVLKSHRRAV FGKYHTSNLK -CIASVGMAP VIMTGLYQVS ALCENA---- ----------

SVLKSHRRAV FGKYHTSNLK -CIASVGMAP VIMTGLYQVS TLCENT---- ----------

--MNKEINRV YEENGVNPLA GCGPALLSFP VLAGLYRAFN NAGI------ ----------

--MNKEINRV YEENGVNPLA GCGPALLTFP VLAGLYRAFN NAGI------ ----------

--MNQKIAEF YQTNEINPLA GCLPSIVQIP VFIGLYRAVL ELAQ------ ----------

--MNQKIAEV YQTNEVNPLA GCIPSLVQIP VFIGLYRAVL NLAK------ ----------

--QNRATAKL YEDAQQNPLA GCFVALIQLP VFLGLYRGVR LLAM------ ----------

KETRKRQKKL FKKYNVPLWK NALLPMVQIP LWVTVSMGIR TLTETQ---- ----------

--VAMQRKKL LSSHGIKNRW -LAAPMLQIP IALGFFNALR HMANYP---- ----------

TALKGERRRV FAEEKTSNAQ -CLSSILGSP IVLFGMFQAK SLCENP---- ----------

TTLKGERRRV FLEEKTSNLQ -CFSSLLGTP VVFFGIVQTT AMCGDP---- ----------

--QNRAISKL FEDAQANPLA GCVTSFAQIP IFLGLYRSVT RLAQ------ ----------

--VAHEKKKI LKKYGISQMK -LFYPMAMFP LTIGIFLGIR RMCEG----- ----------

KERRKRRVKL YKEHGCEMWK SLFIPLVQLP IWITMSLAVR AMCGWTVKV- ----------

--IQLETARL YKLAGINPLA GCLPTLATIP VWIGLYRALS NVAD------ ----------

--IQLETARL YKLAGINPLA GCLPTLATIP VWIGLYRALS NVAN------ ----------

--IQLETARL YKLAGINPLA GCLPTLATIP VWIGLYRALS NVAN------ ----------

--IQLETARL YKLSDVDPLA GCLPTLVTIP VWIGLYRALS NVAN------ ----------

--IQLETSRL YKQAGVNPLA GCLPTLATIP VWIGLYQALS NVAN------ ----------

--IQLETARL YKQAGVNPLA GCFPTLATIP VWIGLYQALS NVAN------ ----------

--IQLETSRL YRQAGVNPLA GCFPTLATIP VWIGLYQALS NVAN------ ----------

--IQLETSRL YRQAGVNPLA GCFPTLATIP VWIGLYQALS NVAN------ ----------

--LQLETARL YKEAGVNPLA GCFPTLATIP VFIGLYNALS NAAK------ ----------

--LQLEQARL YREAGFNPLA GCLPLFATLP VFIGLYRALS NAAA------ ----------

--LQMEQARL YKEAGFNPLA GCLPVFATLP VFIGLYRALS NAAS------ ----------

------V--- ---------- ---------- ESMRDGGLFW FTDLTIC--- ----------

------V--- ---------- ---------- ESMRDGGLFW FTDLTMA--- ----------

---DPAY--- ---------- ---------- TELTLGGFGW IPNLTEL--- ----------

---DPVT--- ---------- ---------- TEMTIGGFGW IPNLTVV--- ----------

------V--- ---------- ---------- EGFSDQGYAW FQNLIEV--- ----------

----WLD--- ---------- ---------- PSLYEEGILW FQDLSIA--- ----------

------V--- ---------- ---------- PSLQTGGLLW FQDLTLS--- ----------

------V--- ---------- ---------- PSLQTGGLWW FQDLTVS--- ----------

------V--- ---------- ---------- PSLQTGGLWW FQDLTVS--- ----------

---SFVQ--- ---------- ---------- EQLATDGILW FPDLTAL--- ----------

---SIVQ--- ---------- ---------- EQLAAGGTLW FPDLTAV--- ----------

---SFVQ--- ---------- ---------- EQLATGGILW FPDLTAP--- ----------

---------- ---------- ---------- DGLLDTQFYF VPSLAGPTTM AMRQSGLGTS

---------- ---------- ---------- DN-LDQGFFW LPSLEGPVR- ----QGQGSS

IEPKPSSKHS IFISETKHFP VIASLPSGTK LGVGDKAQIK LQTLTGTSRL SGVEGGTKFT

IEPKPSSKHS IFISETKHFP VIASLQSGTK LGVGDKAQIK LQTLTGTSRL SGVEGGTKFT

VVPTPSPSKN IFVTDSVHKP VVLVAPKGTK IAVGEQVQFR LQGPGGEQLV AEAGGDTLLR

------E--- ---------- ---------- TDLEMAPFLW VKDLTMP--- ----------

------E--- ---------- ---------- TDLEMAPFLW VKDLTMP--- ----------

------E--- ---------- ---------- TDLEMAPFLW VKDLTMP--- ----------

------E--- ---------- ---------- TDLERAPFLW IVDLTMP--- ----------

------E--- ---------- ---------- SDLEMAPFLW VKDLTMP--- ----------

------E--- ---------- ---------- SDLEMAPFLW VKDLTMP--- ----------

------A--- ---------- ---------- LDVGASSYLW CSALTLP--- ----------

------S--- ---------- ---------- LDVGTSSYLW CTALTLP--- ----------

------S--- ---------- ---------- LDVGTSSYLW CTALTLP--- ----------

---------- ---------- ---------- DGAFDEPWFF LPSLAGPT-- ----DARDLS

---------- ---------- ---------- DGAFDEAWFF LPSLAGPT-- ----DARDLS

---------- ---------- ---------- ANKLDESFLF LPNLEGPTYG A--EPGSAAD

---------- ---------- ---------- ENALDEPFLF LPNLEGPTYG A--DPAHGSD

---------- ---------- ---------- DGVLEEPFLW IPSLEGPVAP ---PNFQGLD

------I--- ---------- ---------- ESFYPSSALG FSSFDLS--- ----------

------V--- ---------- ---------- DGFANQGVAW FTDLTQA--- ----------

------Y--- ---------- ---------- LEFGTSPFLW CTSLTMP--- ----------

------R--- ---------- ---------- LDFGTSSFLW CPALTMP--- ----------

---------- ---------- ---------- EGRLDEPFLW IPSLQGPVTA E--TNYRGTE

------V--- ---------- ---------- QGLSTEGVLW FQNLAAP--- ----------

------V--- ---------- ---------- KSMGTEGALW FPDLLMM--- ----------

---------- ---------- ---------- EGLLTEGFFW IPSLAGPTTV AARQNGSGIS

---------- ---------- ---------- EGLLTEGFFW IPSLAGPTTI AERQNGSGIS

---------- ---------- ---------- EGLLTEGFFW IPSLAGPTAI ASRQNGSGIS

---------- ---------- ---------- EGLLTEGFFW IPSLAGPTTI AARQSGQGIS

---------- ---------- ---------- EGLFTEGFFW IPSLGGPTSI AARQSGSGIS

---------- ---------- ---------- EGLLTEGFFW IPSLGGPTTI AARQSGAGIS

---------- ---------- ---------- EGVLTEGFFW IPSLGGPTTI AARQSGSGIS

---------- ---------- ---------- EGVLTEGFFW IPSLGGPTTI AARQSGSGIS

---------- ---------- ---------- EGLLTEGFFW IPSLGGPTTI -----GGGLE

---------- ---------- ---------- EHLLDDGFYW IPSLGGPTSI AARNDGNGFA

---------- ---------- ---------- EGLLTDGFYW IPSLGGPTSI ASRNAGSGFA

---------- ---------- ---------- ---DQ----- ---------- ---------F

---------- ---------- ---------- ---DP----- ---------- ---------F

---------- ---------- ---------- ---DH----- ---------- ---------S

---------- ---------- ---------- ---DN----- ---------- ---------S

---------- ---------- ---------- ---DP----- ---------- ---------Y

---------- ---------- ---------- ---DP----- ---------- ---------M

---------- ---------- ---------- ---DP----- ---------- ---------T

---------- ---------- ---------- ---DP----- ---------- ---------I

---------- ---------- ---------- ---DP----- ---------- ---------I

---------- ---------- ---------- ---DS----- ---------- ---------T

---------- ---------- ---------- ---DS----- ---------- ---------T

---------- ---------- ---------- ---DS----- ---------- ---------T

WLWPLPDG-- ---------- ---------- ---APPIG-- ---------- ---WEDAAAY

WLFPFQNG-- ---------- ---------- ---APPIG-- ---------- ---WHDAIAY

PTWSVTKGDD LVKVSADGTV QALAEGDATV QGKIPGLAAQ SGFLFIKALG QVGFYVDGEI

PTWSVTKGDD LVKVSADGTI QALSEGDATV QGKIPGLAAQ SGFLFIKALG QVGFYVDGEI

PTWKITKGEE RAQIQPDGTL LALQPGEVTV EVAIPGLASD TGFLFIDKLG RVGFDPDGTI

---------- ---------- ---------- ---DP----- ---------- ---------T

---------- ---------- ---------- ---DP----- ---------- ---------T

---------- ---------- ---------- ---DP----- ---------- ---------T

---------- ---------- ---------- ---DP----- ---------- ---------T

---------- ---------- ---------- ---DP----- ---------- ---------T

---------- ---------- ---------- ---DP----- ---------- ---------T

---------- ---------- ---------- ---DP----- ---------- ---------L

---------- ---------- ---------- ---DP----- ---------- ---------F

---------- ---------- ---------- ---DP----- ---------- ---------F

WLLPLADL-- ---------- ---------- ---APPIG-- ---------- ---WDDASLY

WLLPLSDY-- ---------- ---------- ---APPIG-- ---------- ---WEEASLY

WILKWTDG-- ---------- ---------- ---VPSLG-- ---------- ---WPDTIAF

WLFKWVDG-- ---------- ---------- ---VPGLG-- ---------- ---WEDTIAF

WLVQWVNG-- ---------- ---------- ---APALG-- ---------- ---WETTLAF

---------- ---------- ---------- ---SP----- ---------- ---------L

---------- ---------- ---------- ---DP----- ---------- ---------Y

---------- ---------- ---------- ---DP----- ---------- ---------Y

---------- ---------- ---------- ---DP----- ---------- ---------Y

WLTQWVDG-- ---------- ---------- ---VPPLS-- ---------- ---WETTLAF

---------- ---------- ---------- ---DP----- ---------- ---------Y

---------- ---------- ---------- ---DH----- ---------- ---------S

WLFPFIEG-- ---------- ---------- ---HPPLG-- ---------- ---WPDTLAY

WLFPFVDG-- ---------- ---------- ---QPPLG-- ---------- ---WSDTVAY

WLFPFVDG-- ---------- ---------- ---HPPLG-- ---------- ---WSDTVAY

WLFPFTDG-- ---------- ---------- ---HPPLG-- ---------- ---WSDTLAY

WLFPFVDG-- ---------- ---------- ---HPPLG-- ---------- ---WYDTVAY

WLLPFVDG-- ---------- ---------- ---HPPLG-- ---------- ---WHDTICY

WLFPFVDG-- ---------- ---------- ---HPPLG-- ---------- ---WHDTAAY

WLFPFVDG-- ---------- ---------- ---HPPLG-- ---------- ---WNDTAAY

WLVPFENG-- ---------- ---------- ---APPVG-- ---------- ---WANAAAY

WLWPFVDG-- ---------- ---------- ---HPPLG-- ---------- ---WYETGCY

WLWPFVDG-- ---------- ---------- ---HPPLG-- ---------- ---WHDTTAY

Y----ALPII TSLTLFATIE LGTDSARM-- SAANMQTAKY ILRALPL--F IFPFTINFPG

Y----LLPLI TSATLYLTIE IGTDSARL-- SAANMNTMKY VLRALPI--V IFPFTMNFPA

L----IFPVA LGVINLSIIE IQAASRTKL- PSKLQTIFTN LFRGLSI--L MVPIAASVPS

Y----ILPVA LGLINLAIIE VQAMSRTRP- STRLQNIANN VFRGLSV--V MVPVACTVPS

L----GLQAI SAAAIIAVVR VGGETG-Q-- -HAMAAGMKK VMTVVPI--A SIFITKGFAS

H----VFPVI LGITALCNIE WTLKTLELTK KPTLTDAFGN LTKMSIV--F MMAISLHAPA

Y----ILPLV VTATMWGVLE LGAETG-M-- QSSDLQWMRN LIRVMPL--A VLPITIHFPT

Y----ILPLA VTATMWAVLE LGAETG-V-- QSSDLQWMRN VIRMMPL--I TLPITMHFPT

Y----VLPLV VTATMWCVLE LGAETG-V-- QSNDLQFMRN IIRVMPL--V VLPVTIHFPS

W----ILPIS VGVINLLIVE IFALQKIG-- MSRFQTYITY FVRAVSV--L MIPVAATVPS

W----ILPVS VGVVNLLIVE IFALQKIG-- TSRFQMHVTN FVRAVSV--L MIPVAATVPS

W----ILPIS VGVINLLIVE ICALQKIG-- MSRFQTYITY FVRAMSV--L MIPIAATVPS

L----TLPLL LVAVQYASSS VTSPPDPK-- -DENANTQRA LLVFLPL--M VGWFSLNVPA

L----VLPCL LVVSQSISQK ILQPP-VQ-- -DPQQQQANA ILRFLPF--M VGWFSLNVPS

NWDIAILVGG FGLTLLVSQI LSGRGL---P ANPQQS---T ANKITPVMIT GMFLFFPLPA

NWDIAILVGG FGLTLLVSQI LSGRGL---P ANPQQS---T ANKITPVMIT GMFLFFPLPA

HWDIIGMIVL FGVSIYLNQS LTNAG----Q EDPSQS---S MARITPVLFS AMFLFFPLPA

Y----ALPAI CAGMFLLNFE MNQRMQRGGR S-ASSMYVRW AVRASSV--V GVYFFAAQPS

Y----ALPAI CAGMFLLNFE MNQRMQRGGR S-ASGLYVRW VVRASSV--V GIYFFAGQPS

Y----ALPAI CAGMFLLNFE MNQRMQRGGR S-ASGLYVRW AVRASSV--V GIYFFAGQPS

Y----GLPMI CAGMFLMNFE LNQMMQRGGR S-STGLYVRW GMRVGSV--I GVYFFSSQPA

Y----GLPLI CAGMFLVNFE LNQQMQRGGR S-SSGIYIRW AVRAASL--V GVYMFASQPS

Y----GLPLI CAGMFLVNFE LNQQMQRGGR S-SSGIYIRW AVRAASL--V GVYVFASQPS

L----VLPTL TCLITLLNFE LALSKEIK-- -TGWMRNVIW GARLGCL--C VVPVVSSFRS

A----VLPTL TCVITLLNFE LSLSKEIK-- -TGWMRNVIW GARLGCL--C VIPVLSSFRS

L----VLPIL TCVITLLNFE LSLSKEIK-- -TGWMRNVIW GARLGCL--C VIPVLSSFRS

L----LFPIM TTLSQFVSME VLKPEDEK-- -TKEMQNQSV LLKLLPF--F IGYISLTVPA

L----IFPVL TTISQFVSME VLKPETEK-- -TDEMKNQSV LLKLLPL--F IGYISLTVPA

L----ILPVF LVISQYLSME LMQP-KTD-- -DPAQQQSNA ILKVLPI--M IGWFSLSVPA

L----SIPVF LTISQVISMN MMQP-KTD-- -DPQQQQANV ILKVLPF--M IGWFALNVPA

L----IMPVI LVVLQSVTMQ VLQPPKEE-- -RETLERSQT ILKFLPL--L IGFFALQVPA

L----LAPIL VGTLAVLNVE LNGRLMFSSS QQEAMTSILN VSRLGCV--V MLAMSSQAPF

L----GLQVI TAAVFISFTR LGGETG-AQQ ---FSSPMKR LFTILPI--I SIPATMNLSS

G----ILPLA FCGLTLANFE LSISKELK-- -TGWMSNVVW GARLGCL--C VLPVALQFRS

G----VLPLV FCVLTLMNFE LSISKELK-- -KGWMSNLIW GGRLACF--C ILPAVAQIRA

C----VMPVL LVLGQSFTMN VLQQPEET-- -KKQMESTKT ILKFLPL--M IGFFSLQVPA

L----GLQVI TAAMYMASIR LGSET-GTNN ---LSPGMKK ILQWAPW--I SVPFLMKMPA

G----VLPAA VGIITLLNVE LTTKAQAQGT TPKLPKMMAN FARVGAI--A LFSIAAQTPT

L----VLPLL LVFSQYLSIQ IMQSSQSN-- -DPAMKSSQA VTKLLPL--M IGYFALSVPS

L----VLPAM LVVLQYMSVQ IMQSSQSD-- -DPNVKNSQA IMKFLPL--M IGYFSLSVPS

L----VLPVM LVVSQYISVQ IMQSSQSD-- -DPNVKNSQA ITKFLPL--M IGYFSLSVPS

L----VLPVL LVISQYVSSQ VMQPPQNN-- -DPSQQGAQA VVKFLPL--L IGYFALSVPS

L----VLPVL LIASQYVSME IMKPPQTD-- -DPAQKNTLL VFKFLPL--M IGYFALSVPS

L----VLPVL LVASQFVSME IMKPPQTD-- -DPSQKNTLL VLKFLPF--M IGWFSLSVPS

L----VLPVL LIASQYVSME IMKPPQTD-- -DPTQKNTLL VFKFLPI--M IGYFSLSVPS

L----VLPVL LVVSQYVSME IMKPPQTD-- -DPTQKNTLL VFKFLPL--M IGYFSLSVPS

L----VMPVL LVASQYASQK IISSQNNQ-- -DPSQQQAQA ILKFLPL--M IGWFSLNVPS

L----VLPVL LVVSQFVSQT IISPQKTD-- -DPAQQQSQA ILKFLPF--M IGFFSLNVPA

L----VLPVL LVASQYVSQQ IVSPQKTD-- -DPAQQQSQA ILKFLPL--M IGFFSLNVPA

AILCYWACSN FFSLVQVGFL RIPKVRDFFK IDRIVEE--- ---------- ----------

AILTYWACSN FISLGQVAVL RIPSVREYFK IEKMLEE--- ---------- ----------

CLCLYWVTSS AYGLGQNLLL LSPRVRRTVG IPAVPGS--- ---------- ----------

ALCVYWVASS SFGLAQNLLI LSPEVRRSVG IPKTQRE--- ---------- ----------

AIILYFAVNS IFSLIQSSLF KSSWFRKIAG MPPKLAT--- ---------- ----------

ALTIYWISSQ LYSLLQNVMM DLMLPISFTP KKRIDIN--- ---------- ----------

AVFMYWLSSN MFSLGQVACL RIPAVRTILK IPQRVQE--- ---------- ----------

AVFMYWLSSN LFSLVQVSCL RIPAVRTVLK IPQRVQE--- ---------- ----------

AVFMYWLSSN VFSLCQVACL RIPAVRTVLK IPQRVQE--- ---------- ----------

SIVLYWLCSS LMGLSQNLLL RSPRFRQLCR IPLTKIK--- ---------- ----------

ALVLYWLCSS LMGLAQNLLL RSPGFRQLCR IPPSKLKR-- ---------- ----------

SIVLYWLCSS FVGLSQNLLL RSPGFRQLCR IPSTKIK--- ---------- ----------

GLSLYYLANT VLSSAIQIYL KNVVMNELGP VTKPRAA--- ---------- ----------

GLGLYWVTNN IVSTIQTIGI KRYLASKQPG APRTLTTNG- ---------- ----------

GVLLYMVIAN IFQAGQTFLL SREALPENLQ KILNDSK--- ---------- ----------

GVLLYMVIAN IFQAGQTFLL SREALPENLQ KILNEK---- ---------- ----------

GVLLYILVSN IFQTVQTFLL SREPLPENLQ QLVEEEP--- ---------- ----------

AMFAYWIGLS TAGLLQPVLL RWQPFRDFFR FPEPPDR--- ---------- ----------

AMFAYWIGLS TAGLLQPILL RWQPFRDFFQ FPDPPDK--- ---------- ----------

AMFAYWIGLS TAGLLQPILL RWQPFRDFFQ FPDPPDK--- ---------- ----------

AMFAYWIGLS TAGLLQPLLL RWQPFRDFFK LPDPPLE--- ---------- ----------

AMFAYWIGLS TAGLLQPLLL RWQPFREFFQ FPDPPLE--- ---------- ----------

AMFAYWIGLS TAGLLQPLLL RWQPFREFFQ FPDPPLE--- ---------- ----------

GVCLYLIGMN AVGLLQPLLL RSPVIRRWLR FPSPETQSTT VPWTAVRSRA ATLRERIVAA

GVCLYLVGMN AVGLLQPLLL RSAVIRRWLG FPSAETRSAT VPQTAVSGRA ATLRERIVAA

GVCLYLVGMN AMGLLQPLLL RSAVIRRWFG FPSAETRSAT VPGTAVSGRA ATLRERIVAA

GLALYWFWNN VFTTGIQVYL R--------- ---------- ---------- ----------

GLALYWFWNN VFTTGIQVFL RKATVE---- ---------- ---------- ----------

ALSVYWVINN IITTGTSLII RNSMSVETVT PSGTAGTK-- ---------- ----------

ALGVYWVVNN IVTTATTLYV R--------- ---------- ---------- ----------

GLTIYWFTSN IFTLTQSLAV RAYFSANPPQ IELPDGES-- ---------- ----------

LLSLYWISSQ LFSLVQNIIL NWIYPYQR-- ---------- ---------- ----------

AVVLYFAFNG AFSVLQTMIL RNKWVRSKLK ITEVAIKK-- ---------- ----------

GVCLYFLGMG LVGLLQPILL RSNKFRSFFN FPTEGKFYVA ---------- ----------

GVALYFLGMS LVGLIQPLLL RSSKFRTWFN FPLQQKLHHS RCTAGVS--- ----------

GLTIYWFTSN LFTVSQSLII RGYYAANPPE VKLPDGAT-- ---------- ----------

ALLLHFFVNG ILMLIQGVAL RNPFFRKKLG IHEIVKRK-- ---------- ----------

AVCLYWISSS GFSLIQNVLL NKYPLREEPP MFAAGEH--- ---------- ----------

GLSLYWLTNN ILSTAQQVWL QKNPVEKFTN LVTKETT--- ---------- ----------

GLSLYWLTNN ILSTTQQVWL QK-------- ---------- ---------- ----------

GLSLYWLTNN ILSTAQQVWL QKNPMMKSSD DIV------- ---------- ----------

GLSLYWLTNN ILSTAQQVWL QKNPVKEYID KLAKQGC--- ---------- ----------

GLSIYWLTNN VLSTAQQVYL RKPNMDENAS KIISPT---- ---------- ----------

GLSIYWFTNN ILSTAQQVWL RKPVVNQGGS GIITSKM--- ---------- ----------

GLSIYWFTNN VLSTAQQVWL RKPVVNENAS GIITGRT--- ---------- ----------

GLSIYWFTNN VLSTAQQVWL RKPVVNENAS GIITKA---- ---------- ----------

GLTLYWFVNN LLSTGQQLYL KKVNIPEAIK APATSAS--- ---------- ----------

GLTLYWFFNN IITTAQTVVL RKITKPIEVP TGAGARR--- ---------- ----------

GLTLYWFANN IITTAQTLIL RKTTTAPEPP VAKS------ ---------- ----------

---------- ---------- ---------- ---------- ---------- ----------

---------- ---------- ---------- ---------- ---------- ----------

---------- ---------- ---------- ---------- ---------- ----------

---------- ---------- ---------- ---------- ---------- ----------

---------- ---------- ---------- ---------- ---------- ----------

---------- ---------- ---------- ---------- ---------- ----------

---------- ---------- ---------- ---------- ---------- ----------

---------- ---------- ---------- ---------- ---------- ----------

---------- ---------- ---------- ---------- ---------- ----------

---------- ---------- ---------- ---------- ---------- ----------

---------- ---------- ---------- ---------- ---------- ----------

---------- ---------- ---------- ---------- ---------- ----------

---------- ---------- ---------- ---------- ---------- ----------

---------- ---------- ---------- ---------- ---------- ----------

---------- ---------- ---------- ---------- ---------- ----------

---------- ---------- ---------- ---------- ---------- ----------

---------- ---------- ---------- ---------- ---------- ----------

---------- ---------- ---------- ---------- ---------- ----------

---------- ---------- ---------- ---------- ---------- ----------

---------- ---------- ---------- ---------- ---------- ----------

---------- ---------- ---------- ---------- ---------- ----------

---------- ---------- ---------- ---------- ---------- ----------

---------- ---------- ---------- ---------- ---------- ----------

VAPADDPAAA GARAKANDAD DDVLKVRGRA RLQGQQYTAS THAAAAPVHG SRYARGTNPL

VAPAEKPAAA GVTVKASDAA ADVLKFSGRG RLQGQQCTAF AYSGAAPAHG SRYARGANPL

VALTENPAAA GVTVNASDAA ADVLRFCGRA HLQGQQCTAF AYSGAAPAHG SRYARGANSL

---------- ---------- ---------- ---------- ---------- ----------

---------- ---------- ---------- ---------- ---------- ----------

---------- ---------- ---------- ---------- ---------- ----------

---------- ---------- ---------- ---------- ---------- ----------

---------- ---------- ---------- ---------- ---------- ----------

---------- ---------- ---------- ---------- ---------- ----------

---------- ---------- ---------- ---------- ---------- ----------

---------- ---------- ---------- ---------- ---------- --------PV

---------- ---------- ---------- ---------- ---------- ---TRGMNPI

---------- ---------- ---------- ---------- ---------- ----------

---------- ---------- ---------- ---------- ---------- ----------

---------- ---------- ---------- ---------- ---------- ----------

---------- ---------- ---------- ---------- ---------- ----------

---------- ---------- ---------- ---------- ---------- ----------

---------- ---------- ---------- ---------- ---------- ----------

---------- ---------- ---------- ---------- ---------- ----------

---------- ---------- ---------- ---------- ---------- ----------

---------- ---------- ---------- ---------- ---------- ----------

---------- ---------- ---------- ---------- ---------- ----------

---------- ---------- ---------- ---------- ---------- ----------

---------- ---------- ---------- ---------- ---------- ----------

---------- ---------- ---------- ---------- ---------- ----------

---------- ---------- ---------- ---------- ---------- ----------

---------- ---------- ---------- --T--

---------- ---------- ---------- --Q-G

---------- ---------- ---------- -----

---------- ---------- ---------- --A-K

---------- ---------- ---------- --NRH

---------- ---------- ---------- -----

---------- ---------- ---------- --SLG

---------- ---------- ---------- --NLG

---------- ---------- ---------- --NLG

---------- ---------- ---------- -----

---------- ---------- ---------- -----

---------- ---------- ---------- -----

---------- ---------- ---------- --ARL

---------- ---------- ---------- DA-RR

---------- ---------- ---------- -----

---------- ---------- ---------- -----

---------- ---------- ---------- -----

---------- ---------- ---------- --P-R

---------- ---------- ---------- --R-R

---------- ---------- ---------- --R-R

---------- ---------- ---------- --VKD

---------- ---------- ---------- --K-H

---------- ---------- ---------- --K-H

MRETPLHRQE PSSSRVSPHE GGRGSGSSGA AAH-K

MQETPLHRQA PSSSHASPHE SGGAGDSSGD AAH-K

MQETPLHRQA PSSSHVSPHE NGGAGDNSGA AAH-K

---------- ---------- ---------- -----

---------- ---------- ---------- -----

---------- ---------- ---------- ----N

---------- ---------- ---------- -----

---------- ---------- ---------- ----S

---------- ---------- ---------- -----

---------- ---------- ---------- -----

WF-------- ---------- ----GGAQSA MGYRS

MR-------- ---------- ----GETARP MGK-Q

---------- ---------- ---------- ----V

---------- ---------- ---------- -----

---------- ---------- ---------- -----

---------- ---------- ---------- ----F

---------- ---------- ---------- -----

---------- ---------- ---------- -----

---------- ---------- ---------- ----F

---------- ---------- ---------- ----V

---------- ---------- ---------- ----V

---------- ---------- ---------- ----V

---------- ---------- ---------- ----V

---------- ---------- ---------- ----A

---------- ---------- ---------- ----N

---------- ---------- ---------- -----

64 598

Aga_Oxa1 LE-------- ---------- ---------- ---------- ---------- ----------

Dme_Oxa1 MTTNAM---- ---------- ---------- ---------- ---------- ----------

Aga_Oxa2 ---------- ---------- ---------- ---------- ---------- ----------

Dme_Oxa2 MRRSI----- ---------- ---------- ---------- ---------- ----------

Cal_Oxa1 MTRLF----- ---------- ---------- ---------- ---------- ----------

Cal_Oxa2 ---------- ---------- ---------- ---------- ---------- ----------

Cfa_Oxa1 ---------- -MALGFM--- ---------- ---------- ---------- ----------

Hsa_Oxa1 MLRLLQFWLC FLAMGLM--- ---------- ---------- ---------- ----------

Cfa_Oxa2 MLR------- ---------- ---------- ---------- ---------- ----------

Hsa_Oxa2 MLRLA----- ---------- ---------- ---------- ---------- ----------

CreAlb3.1 MSRMG----- ---------- ---------- ---------- ---------- ----------

Ctr_YidC MDKRLFVILS LTLFFQHQNT QQKQQQSKLE ESLTTIYEIY RYDLQLGLAL LKQGYLAEIL

Cca_YidC MKRSLFVSIG IAIFFGYNDF RSCRIEVLAT KSMWTTSNIH RNNQEPGGVF WRNEYIEGIL

Cch_YidC MNSTGLALIA VIQFMTP-AQ K----QPLKS LVL------- ---------- ----------

Cph_YidC MNSIGLVLIG LIQFMAP-EN K----VELKS MVL------- ---------- ----------

Pma1_YidC M--------- ---------- ---------- ---------- ---------- ----------

Pma2_YidC M--------- ---------- ---------- ---------- ---------- ----------

Syn_YidC ---------- ---------- ---------- ---------- ---------- ----------

Lga_YidC1 ---------- ---------- ---------- ---------- ---------- ----------

Ljo_YidC1 ---------- ---------- ---------- ---------- ---------- ----------

Lga_YidC2 MKNI------ ---------- ---------- ---------- ---------- ----------

Ljo_YidC2 MKNI------ ---------- ---------- ---------- ---------- ----------

Lac_YidC2 MRNL------ ---------- ---------- ---------- ---------- ----------

Lac_YidC1 ---------- ---------- ---------- ---------- ---------- ----------

Msy_YidC MV-LIYTFFF GTQNF----- ---------- ---------- ---------- ----------

Mag_YidC MKKVIYVLLF GAQSC----- ---------- ---------- ---------- ----------

Ota_Alb3.1 ---------- ---------- ---------- ---------- ---------- ----------

Olu_Alb3.1 ---------- ---------- ---------- ---------- ---------- ----------

Sus_YidC MVRLAFMG-- AVYIFPT--- -------VRS WQL------- ---------- ----------

Aba_YidC MRKLIFALTF IMTVFFK--- -------VKS WVL------- ---------- ----------

Lbo_YidC MQTRLALILS MGIYFFFPNT STKKNTEISK FYM------- ---------- ----------

Lin_YidC IKKNIAVVLS VLVFLLYPSA SSSHSETILS YQL------- ---------- ----------

Yli_Oxa1 MRRASLRPSL FPE------- ---------- ---------- ---------- ----------

Yli_Oxa2 MTR------- ---------- ---------- ---------- ---------- ----------

Cdi_YidC1 ---------- ---------- ---------- ---------- ---------- ----------

Cdi_YidC2 ---------- ---------- ---------- ---------- ---------- ----------

Sco_YidC ---------- ---------- ---------- ---------- ---------- ----------

Sav_YidC ---------- ---------- ---------- ---------- ---------- ----------

Fra_YidC ---------- ---------- ---------- ---------- ---------- ----------

Pbe_YidC -RNVIALVLS TLLALVLGPK RAAYLAQFDD LIL------- ---------- ----------

Rpa_YidC MRNTLAVILS GLVYFFNIPQ MEKQRTQLDD IAL------- ---------- ----------

Bsu_YidC1 ---------- ---------- ---------- ---------- ---------- ----------

Gka_YidC1 ---------- ---------- ---------- ---------- ---------- ----------

Bsu_YidC2 MT-------- ---------- ---------- ---------- ---------- ----------

Gka_YidC2 MKKV------ ---------- ---------- ---------- ---------- ----------

Mba_YidC MRRLLVLIFT FSSNWQKYNQ PP--AAALVS LEL------- ---------- ----------

Dar_YidC MKRLIFVLLS VGIEYFMPKH TP---QQLRS LKL------- ---------- ----------

Ppr_YidC -KRTLAVILS ITVLLFAPEK KP---QPLKS LTL------- ---------- ----------

Dde_YidC MKRPIAVVLS LFVYLSEFMG TP---PALKH FRL------- ---------- ----------

Cfe_YidC MKRLIATVIS ILFYFFIPKR TV---INISS FVL------- ---------- ----------

Cla_YidC MKRIIAVVLS FLFYFFIPKA PQ---T-IAK FYL------- ---------- ----------

Lpn_YidC MRRILYMALA LIGAWQIDYP K----PEIVK GLL------- ---------- ----------

Xca_YidC MTRVLIFAWL MVAEWGKDKA PN--ATIVLD AEL------- ---------- ----------

AthAlb3.b ---------- ---------- ---------- ---------- ---------- ----------

PAlb3.b-1 ---------- ---------- ---------- ---------- ---------- ----------

PAlb3.b-2 ---------- ---------- ---------- ---------- ---------- ----------

OsaAlb3.b ---------- ---------- ---------- ---------- ---------- ----------

AthAlb3.a MTAF------ ---------- ---------- ---------- ---------- ----------

OsaAlb3.a MLPL------ ---------- ---------- ---------- ---------- ----------

PAlb3.a-1 MT-------- ---------- ---------- ---------- ---------- ----------

PAlb3.a-2 MT-------- ---------- ---------- ---------- ---------- ----------

CreAlb3.2 ---------- ---------- ---------- ---------- ---------- ----------

OtaAlb3.2 ---------- ---------- ---------- ---------- ---------- ----------

OluAlb3.2 ---------- ---------- ---------- ---------- ---------- ----------

---------- ---------- ---------- ---------- ---------- ----------

---------- ---------- ---------- ---------- ---------- ----------

---------- ---------- ---------- ---------- ---------- ----------

---------- ---------- ---------- ---------- ---------- ----------

---------- ---------- ---------- ---------- ---------- ----------

---------- ---------- ---------- ---------- ---------- ----------

---------- ---------- ---------- ---------- ---------- ----------

---------- ---------- ---------- ---------- ---------- ----------

---------- ---------- ---------- ---------- ---------- ----------

---------- ---------- ---------- ---------- ---------- ----------

---------- ---------- ---------- ---------- ---------- ----------

PFRTDENQEE FDRVKHHPYN AKDQYPLLRR DLIQIRKSQH YALNIVSYEL AELIYEVIHF

PFSSEDNKEG FDRKAQVPSE ASFPYPLLRR GILSDAKKSY HALNIVSLNS VASGYRVSTF

--------KK HLDGHLQDLL GKQLTKDTAP QKAIEIAYLF SGESYAIDYD VKLIGFGNDI

--------KK HLNANREDLI KNSQTSDDAP EKSITIVYTF TGDVYGINYD VQFTGFDGVL

---------- ---------- ---------- ---------- ---------- ----------

---------- ---------- ---------- ---------- ---------- ----------

---------- ---------- ---------- ---------- ---------- ----------

---------- ---------- ---------- ---------- ---------- ----------

---------- ---------- ---------- ---------- ---------- ----------

---------- ---------- ---------- ---------- ---------- ----------

---------- ---------- ---------- ---------- ---------- ----------

---------- ---------- ---------- ---------- ---------- ----------

---------- ---------- ---------- ---------- ---------- ----------

---------- ---------- -------VSN GINVVPRVAQ DEQSQRFGIK YDYSSDAYPT

---------- ---------- -------VSN GITVSPHIYL IN--EKFTSK LEVNRNSTPF

---------- ---------- ---------- ---------- ---------- ----------

---------- ---------- ---------- ---------- ---------- ----------

--------KR YKGN------ ---------- DVVVRKVVRF QK-----SVS CEVT------

--------KN YKDD------ --------HG DLSAKKTLRF TN-------- -------GVE

--------KD FTGPNGRPET VVVSR-SDAR SYQLKKEFRF YPSENYFKLS ISIINFSKEK

--------RE HYAAQRRV-- EMVEQLALRT PFVLAKRYVF YPDNYMFELH VSLSADVLEE

---------- ---------- ---------- ---------- ---------- ----------

---------- ---------- ---------- ---------- ---------- ----------

---------- ---------- ---------- ---------- ---------- ----------

---------- ---------- ---------- ---------- ---------- ----------

---------- ---------- ---------- ---------- ---------- ----------

---------- ---------- ---------- ---------- ---------- ----------

---------- ---------- ---------- ---------- ---------- ----------

--------KT YRATLAAPPI VLLMRAGLGD -IQYRATLAV D-EQFMFRVT YEVTNGSGET

--------VQ FRETVDPPAI ELFAESGWGE GLTFRRTIAV D-ERYLFTMK DEVSNIGNAP

---------- ---------- ---------- ---------- ---------- ----------

---------- ---------- ---------- ---------- ---------- ----------

---------- ---------- ---------- ---------- ---------- ----------

---------- ---------- ---------- ---------- ---------- ----------

--------LN YKEHDDINFD LFDAQAGLQN GIKVAKILTF KRGSYLIDIA WEVANGSDKA

--------LK HGAAEDHPFQ LFEAQTGLAN GVKVSKVYTF KKNSYEIGVR YDIVNGGAAP

--------KN YREK-NLP-- QAVVLGSTGQ GFTVRKIYTF SGDSYGIKLD TQVFNNMAVP

--------KK YTESIEEP-- LVELVGLDMD GVRFERTLSF DPETYLISEK VRLTDTAGVS

--------NE SKFKDENQIS LVSIR--VLN GVTLTKTITF YPDAYDLKVK LS--------

--------SD EKYKDENSIN LVDIR--ILN NLVVTKKITF HKYNYDLEVN LS--------

--------LD YPSVEDKPFP LLQANSSLED GLDVKKEFVF TKGSYLIEVN YKIANTGNSL

--------LQ FPQTKDGPVK LLTATSGWPN GVSIRRIFTL QRGSYAISIK DEVINKSDAA

---------- ---------- ---------- ---------- ---------- ----------

---------- ---------- ---------- ---------- ---------- ----------

---------- ---------- ---------- ---------- ---------- ----------

---------- ---------- ---------- ---------- ---------- ----------

---------- ---------- ---------- ---------- ---------- ----------

---------- ---------- ---------- ---------- ---------- ----------

---------- ---------- ---------- ---------- ---------- ----------

---------- ---------- ---------- ---------- ---------- ----------

---------- ---------- ---------- ---------- ---------- ----------

---------- ---------- ---------- ---------- ---------- ----------

---------- ---------- ---------- ---------- ---------- ----------

---------- ---------- ---------- ---------- ---------- ----------

---------- ---------- ---------- ---------- ---------- ----------

---------- ---------- ---------- ---------- ---------- ----------

---------- ---------- ---------- ---------- ---------- ----------

---------- ---------- ---------- ---------- ---------- ----------

---------- ---------- ---------- ---------- ---------- ----------

---------- ---------- ---------- ---------- ---------- ----------

---------- ---------- ---------- ---------- ---------- ----------

---------- ---------- ---------- ---------- ---------- ----------

---------- ---------- ---------- ---------- ---------- ----------

---------- ---------- ---------- ---------- ---------- ----------

DNRSISGVPE VEWISGGPTT SIYPDWICNS NGFLGMILDP LKEIDPGFKI QAISGLIEID

NSTMLSGIPE VEIMSNAALS GVYPQWILNS NGYFGIILSP LTDIPAGYAA SYVPGLSLLS

AGNEYQVQWD GGLAEVFREQ SGEATWVGVR NKYFTAALIP QSKSNGIYLE GKREAAL---

PGNEYQLEWD GGLLKEYREQ SGVAGWIAVR SKYFVASLIP SSPTDGVYLK GSGKAAL---

---------- ---------- ---------- ---------- ---------- ----------

---------- ---------- ---------- ---------- ---------- ----------

---------- ---------- ---------- ---------- ---------- ----------

---------- ---------- ---------- ---------- ---------- ----------

---------- ---------- ---------- ---------- ---------- ----------

---------- ---------- ---------- ---------- ---------- ----------

---------- ---------- ---------- ---------- ---------- ----------

---------- ---------- ---------- ---------- ---------- ----------

---------- ---------- ---------- ---------- ---------- ----------

L--------- ---KYSRDVL QNNSPLF--- -YLTPKQ--- ---------- ----------

LSFSSKNDFT GFAKFRRDIL EDKIKNF--- -YALKYY--- ---------- ----------

---------- ---------- ---------- ---------- ---------- ----------

---------- ---------- ---------- ---------- ---------- ----------

--------LD KPIPAKGPIS EGTFSFAGIA DTYFAAVFLR ---------- ----------

TSVTRGGSNV TALPIVGGAL RGPFNWAGAQ DQYFAALFLA ---------- ----------

LSFASQKRYL RTFGFSIKTK ESGVDFAGTG SRYFIAVADP LDHKPHGIVL DNRPL----V

REDSRQGAKV RAVAYALKGG REQVDWASVS GKYFALIVLP NDADSLKRLV LSAPQHHIAF

---------- --------KV LDK------- ---------- ---------- ----------

---------- ---------- ---------- ---------- ---------- ----------

---------- ---------- ---------- ---------- ---------- ----------

---------- ---------- ---------- ---------- ---------- ----------

---------- ---------- ---------- ---------- ---------- ----------

---------- ---------- ---------- ---------- ---------- ----------

---------- ---------- ---------- ---------- ---------- ----------

VVAQPYGAAY QRGISGGGID RGSGGWVGIT DKYWLSAAIP PQDTPFKAEL DVTDGRASYL

VTLYPFALIS RHGAEAKAVF KVTNGWLGIT DKYWASALLP DTTAQLQARF SSNLAQTDYL

---------- ---------- ---------- ---------- ---------- ----------

---------- ---------- ---------- ---------- ---------- ----------

---------- ---------- ---------- ---------- ---------- ----------

---------- ---------- ---------- ---------- ---------- ----------

IAPHAYYQLQ RDDLNKAKFK TADNGWLAMV QHYFVSAWVP KDKTQREFYM RKVEGQAGVI

LAPTAYYRLL RDSQGKGDYK TTTDGWVAML QHYFMSAWIL KDERSCRFEL KETAGSAAAL

LVGTV---QQ VMTYSSASKY DKNLQWSGFA DKYFLTAILS EGGSIASVEL RKNGAESTVS

RDVRLGMTLS TTSLRETGVV DEGVNWGGVM CNYFMAVMAP AEGALPFKGV L-EGGRSVVE

-----KNEDY FITPKAKGDR FTGVDILANS DRYY-TTLFY EVDKTLDVYI QNDSAENTL-

-----KDAAY FITPDVENDS FSNVTLMAAS DRYY-SAFFY NFEKPLSAVV TKDNHENSI-

WKGYFNTQLL RSSPSKSNLV DAKGGWIAMQ QHYFLSAWVP NADSENKFYT LATDKTIGAV

WNGYVFRKLS RPTIDDGGLR QITGGWVALL QHHFFTAWIP QKDQASLYVL AQDGPVAELR

---------- ---------- ---------- ---------- ---------- ----------

---------- ---------- ---------- ---------- ---------- ----------

---------- ---------- ---------- ---------- ---------- ----------

---------- ---------- ---------- ---------- ---------- ----------

---------- ---------- ---------- ---------- ---------- ----------

---------- ---------- ---------- ---------- ---------- ----------

---------- ---------- ---------- ---------- ---------- ----------

---------- ---------- ---------- ---------- ---------- ----------

---------- ---------- ---------- ---------- ---------- ----------

---------- ---------- ---------- ---------- ---------- ----------

---------- ---------- ---------- ---------- ---------- ----------

---------- ---------- -----TIPET LGGGWTPVGI VQN-CMEFLH IG--------

---------- -------TSA AGRPDIPAAS IGGGWSPVGM VQN-CLEFLH CT--------

---------- ---------- ---------- -TSQSAPVAY VQQ-GMINLH DL--------

---------- ---------- -------ASV LTSNSTPVAY MQD-VLIKIH DY--------

---------- ---------- ---TILENSS IGQGWGPTSL IER-LLEVTH VY--------

---------- ---------- --------MN RSDHNAIINT MTS-SFQTVH EF--------

---------- --RRLRLLQP RIPATPATQE LGGSYTPVGL IQN-LLEFMH VN--------

---------- --RRLRLLQS GVAATPAPQE LGGSYTPVGL IQN-LLEFMH VD--------

---------- ---------- -----GGWYE ALAASAPVRG AEE-MLLGLH AA--------

---------- ---------- ---LAVHWYE ALAASSPVRV AEE-VLLGVH AA--------

---------- -------RLH RTLQSVMTRA GGWVAPVADA LEQ-VLYALQ EGLD---KLH

QEYNLYQPGA YLKSQGGLMN FRFFAGPD-D HGWFTFISEP FAK-FLLILM KFFH-----Y

PKNQAYPPGT LLPQKEGTHR FLVYAGP-AD RGFFAFITEP FAA-LLFIIM KFFR-----M

-KM-----SL PASATEVHNT FTMYVGPDYD FGWDW-LTRP FAEWMILPVF NWLN-----G

-KF-----TL PQNEKTHSES LVLFVGPDYD FGWDW-LTRP FAEYIILPIF DLLS-----K

---------- ---------- ---------- --IGYISDNI LLP-ILDFFY GL--------

---------- ---------- ---------- --IGYISDNI LLP-ILDFFY GL--------

---------- ---------- --------MD FGVGFLSNNV MLP-ILDFFY GI--------

---------- ---------- ---MFSLMLP YAVFSLFGKP IQN-IMLAVE HQ----IG--

---------- ---------- ---MFSLMIP YAIFSLFGKP IQN-IMLAVE HQ----IG--

---------- ---------- ----LGLMLS GNWDRYIVYY ISQ-FILWIA SL--------

---------- ---------- ----LALMLS GNWDRYIVYY ISQ-FILWIA SL--------

---------- ---------- ----AVLVIS GNWDRWIVYY MSA-FILWLA KL--------

---------- ---------- --MYMGAVLI YGVYQWLGRP LQN-IMIQTA HM----IG--

---------- -------YSL VNYYSNRKVQ YGFYGLIVYP FSA-LINAIT NSLPS----L

---------- -------NKA LNKYASENFK LGFYGLLVFP TAW-LSAHLS TSLSH----L

---------- ---------- --------RM EGWLAPVSNA LED-LLFTIK GQLL---DLG

---------- ---------- --------RM EGWLAPVSDA LED-LLFAIQ GQLQ---GLG

---------- ---------- MQQVTFATVD FGWLSVLAKP LFL-IVNYVN DT--------

---------- ---------- ASMVTLHTLD FGYLGFIAKP LFL-WLRWTH DH--------

YDN-----IL LNPGENYNLD FASYIGIESN QGITTPFRNG IIW-ILKQIY RF--------

VR-------R AVAQPAVADV YRVYIGPAES GGILYPLEVL LKW-LLRLFY TL--------

---------- ----VAPIQT HIQQFPPGIT GSWSLWPSDI YLN-LLEHVH VY--------

---------- ---------- --RARGSART SQSTVDIVRP VET-ALNAIH DF--------

---------- ---------- ---------- --MLNFIYWP ISA-ILWFWH KVVSFVLDPG

---------- ---------- ---------- --MLEMFIYP VSG-VMRLWH YIFADLFGCS

---------- ---------- ---------- --LFSFITWP VSW-VIVQFH TVYGAIFGPD

---------- ---------- ---------- --LFSFITIP VSW-VIVQFH SVYGKVFGAD

---------- ---------- ---------- --MLDPLYHL AAN-AIVFFH KGFGPIFGAD

LET-----QS IAPGATIRTE AYLYGGSEVD WGHFSFLTRP IFG-ILAFFE GL--------

LDP-----QV VPIGGTGSAN TRLFAGAEAD WGWFYFITKP MFV-ALDFFY HL--------

---------- ---------- -MIGLSMGVS PHWDKYVVYP LSE-LITYVA KL--------

---------- ---------- --IRILFALS KGWNEYIVYP LSW-LIKYVA GL--------

---------- ---------- QKLAMGILIN VGFHDYLIEP FSA-LLKGVA GL--------

---------- ---------- ------LGAV QGWNHYFVYP MSK-LLLTLG HW--------

VPV-----AE IAPGAKGEAS VSLYAGPMQD YGWLTVVAAP IFW-ALEAIH KL--------

VDY-----AT VAPGKSLSVS VPLYAGPEYD FGIFYIFASP LFW-LLVKLH GL--------

SPR-----IT VTPGQSVTVV HRLFVGPDID LGWFTVIAKP LLY-TLKYFY RY--------

NSS-----LS IASGSSAEFG IGYYIGPESN YGWFTFLAKP LVS-GLKFFY SY--------

---------- SFVKSSGSFS TKGYIGPDHE YGWFTFIAKP MFL-LLSWLH NY--------

---------- VFASASNTFK ASGYIGSEHE YGWFTFIAKP MYE-FLDFLH GY--------

SQP-----IT VKPKEDKIVG SKLYIGPITD YGILWFLSSL LFS-LMKAIY TV--------

GPA-----FT VAPGQSASTE ARLWVGPLVD YSRFSIMGQG LFW-VLSHLH SF--------

---------- ---------- -----MLFQS NDWFSGIANY MET-ILKVLK DGLS---TVH

---------- ---------- --------QN SDWLSGITSC LES-TLKVLK DGLS---ALH

---------- ---------- --------QN SDWLSGITYG LES-TLKVLK DGLS---AVH

---------- ---------- -----MAFAA GDWLSGITNS MET-VLKVLK DGLS---ALH

---------- ---------- ---LPYNNKS GGWFGFISDA MEL-VLKILK DGLS---AVH

---------- ---------R GGRRGGGGKN GGWFGFISEA LEV-VLKVLK DGLS---AVH

---------- ---------- ----NISRKN GGWFGFISDG MEF-VLKVLK DGLS---SVH

---------- ---------- ----NISRKS GGWFGFISDG MEF-VLKVLK DGLS---AVH

---------- ---------- ------MLAE GGPIDVLAQF FEF-VLQTLD EGLE---SAK

---------- ---------- -------MQK GGWLGPITDA LEG-ALRGID GVL----DGK

---------- ---------- --------QK GGWLGPITDG LES-ALEGID SVL----DGR

LDLPWWGCIA IGTVCVRT-L LFPLVIASQR NAA----KMN NYMPQ----L QVLQMKMTEA

WDIPWWGTIA IGTLAVRT-I IFPLVILAQR NSA----KMN NNMPQ----M QMLQLKMTEA

TGLPWWATVI LTTVGLRTLV TLPLAVYQNK ILA----RLE QISLE----M PELIKELKAE

SGLPWWASIV LSTFLFRSVV TLPLTIYQHK ITA----RIE KIALE----M PAIVEELKKE

TGLPWWGTIV VATIAVRL-V LFPLYVRASS NAT----KMS KIKPQ----I DELLQQIKTG

SGLPWWALIP LTTFTLRSVW TLPLAILQRK RIQ----KQS QLRPL----V SAMNPILKLN

LGLPWWGAIA ACTVLARC-L VFPLIVKGQR EAA----KIH NHLPE----I QKFSTRIREA

LGLPWWGAIA ACTVFARC-L IFPLIVTGQR EAA----RIH NHLPE----I QKFSSRIREA

AGLPWWACIG LGTVALRGAV TLPLAAYQHY ILA----KVE NLQPE----I KNIARHLNQE

TGLPWWGSIL LSTVALRGAV TLPLAAYQHY ILA----KVE NLQPE----I KTIARHLNQE

VPYSYGYSII LLTLIVKL-L TYPLTKQQVE SAM----AVQ ALKPR----I DLIKDRFGE-

LTNSWALSIV LLTVSLRL-M LYPLNTWSTK SMV----RMQ QIAPQ----V TAIQEKYKK-

ITGSWGISII LLTVFLKL-L LYPLNAWSIR SMR----RMQ KLSPY----I QEIQQKYKK-

FISNYGIIII IFAFLVKL-V TYPLSMASTK SMK----KMA ALQPV----L QELQVKYKD-

LTSNYGLIII IFALLIKL-V TYPLTMASTK SMK----KMA ALQPM----M KELQEKYKD-

-VPSYGLAIV ALTVVIRL-A LFPLSAGSIR SAR----RMR IAQPAMKKRQ DEIKSRYAK-

-VPSYGLAIV ALTVVIRL-A LFPLSAGSIR SAR----RMR IAQPAMKKRQ DEIKSRYAK-

-VPSYGLAII FLTLVIRF-A LYPLNVGSIR NMR----RMK VINPLMQRRM REIQEKYRD-

GSNGAGWAII IITFVVQL-I VMPLRLASQR KMTTQQEKTQ KLQPQ----M KLIQEALKKP

GSNGAGWAII IITFVVQL-I VMPLRLASQR KMTTQQEKTQ KLQPQ----M RLIQEALKKP

VRDSYGWAII IFTIIVRI-I LLPLNAISIK SMA----KQQ KVQPQ----M DALRKKYPGK

VHDSYGWAII IFTIIVRI-I LLPLNAISIR SMA----KQQ KVQPQ----M DALRKKYPGK

MGNSYGWAII VFTIIVRV-I LLPLNAISIR STT----KMQ SIQPQ----I NELRKKYPGR

GENGAGWGIV IITFVVRL-I LMPLMLVQQN KSVRQQEKMA RLQPQ----M KLIQNAMKHK

DGWSSIIAIL VLVLLTRL-F SLFITFKATI VQS----AQE DLRVK----K AAIENKYKGF

GGWGTIIVIL VLTIILRS-A MLAVTFKQTV NQS----KQE ELKSK----K AKIDAKYADF

VPYPTGNAII IVTILVKM-V TYPLTKDQVV SSL----NMK NLQPQ----I AAIREKYED-

VPYSTGNAII IVTILVKF-V TYPLTRDQVV SSL----NMK NLQPQ----I AAIREKYED-

LVHNFGWAIV LVTIAINF-I LFPLKLSNMK SMR----KMQ ALKPQ----V DAINAKYKNV

WVPNWGWSII ILTVIINL-V LLPLRLSSMK SAL----KMQ KIQPQ----M KAIQEKYKKY

TIPNYGWSII IFAILFKL-V FYPLNQKQAD SMK----KMQ ELSPQ----L KTINEKFAN-

-IPNWGVAII LVTIAIKV-L FFPLTKRSFI AMQ----KMQ ELQPH----M QRIQERYKG-

TGLPWWAAIA STTVIVRV-L LFPLFVQAAN EQG----KMS EVKPE----L NVIDEKLKSA

SGLPWWAVIP LVTLTLRSTV TLPIAISTRL RAQ----KQH ELRPL----I SALGPILRAK

SGLSWVLAIV LLTFTIRA-L LVKPMLNQMR SMR----KMQ ELQPL----M QEIRKKYPN-

QSQAWVASLF ALVVTVRS-I IAPFSWMQFK SGR----FAI MMRPK----I KRLKEEYAE-

TGWAWGLSIV SLVILIRI-C LIPLFVKQIK ATR----GMQ TLQPE----M KKIQERYKN-

TGWAWGLSIV SLVILIRI-C LIPLFVKQIK ATR----AMQ TLQPE----M KKIQERYKN-

SFFAWAFSVV LLVICVRI-L IFPLFVKQVK SQR----TMQ MMQPR----I KEIKEKYGH-

-TGNWGVAIL LLTLVIKA-V LFPLANMSYK SMA----GMK KVQPE----L MKIRERYTD-

-VGNFGLSIL FVTVIIKL-L FLPLANKSYA SMA----KMK AIQPQ----L QALKDRHPD-

TGDNYGLSII LVTILIRL-L ILPLMIKQLR SSK----AMQ ALQPE----M QKLKEKYSSK

LGGSFGLSII VVTILIRL-L ILPLMIQQTR NAK----AMQ ALQPE----I EALRKKYSSK

FHGEYGLSII LVTIIVRI-V VLPLFVNQFK KQRIFQEKMA VIKPQ----V DSIQVKLKKK

FGDNYGIAII VLTLIVRF-C LLPLILKQFR ASL----AMQ KLRPE----L LKLQEKYKSK

-VGNWGWAIV VLTIMIKA-V FFPLSAASYK SMA----KMK MLTPR----L AQLKERFGD-

-VSNWGWAIV LLTLTVKA-V FYPLTAASYR SMA----KMK ALAPR----L ERLKAQHGD-

-VGNYGVAII IITIILKA-L FFPLTHKSYK SMK----DMQ KIQPM----M AALKEKYKD-

-AGNYGVAII ILTILVKL-L FWPLSQKSYK SME----QMK KLQPM----V QKIKEKYGD-

-IGNWGFAIV ALTIVIRI-V LFPLTYKGMV SMN----KLK DLAPK----M KEIQAKYKG-

-LGNWGWAIV IMTLIVRI-I LFPLTYKSMI SMN----KLK DLAPK----M KEIRERYKG-

-VGNWGWSIV LVTVLIKL-A FYRLSATSYK SMA----SMR KLQPK----L QALRERYGD-

-LHNWGWAII GLVVLLRL-A LYPLSAAQYK SGA----KMR RFQPR----L AQLKERYGD-

VPYSYGFAII LLTVLVKA-A TFPLTKKQVE SAM----AMK SLTPQ----I KAIQERYAG-

VPYAYGFAII LLTVLVKA-A TFPLSKKQVE SAM----AMR SLQPQ----I KAVQQLYAG-

LPYAYGFAII LLTVLVKA-A TFPLSKKQVE SAM----AMR SLQPQ----I KAIQQRYAG-

VPYPYGFAII LLTVLVKA-A TFPLTKKQVE SAI----AMR SLQPQ----V KAIQERYAG-

VPYAYGFAII LLTIIVKA-A TYPLTKQQVE STL----AMQ NLQPK----I KAIQQRYAG-

VPYSYGFAII LLTVIVKA-A TLPLTKQQVE STL----AMQ NLQPQ----I KAIQQRYAG-

VPYAYGFAII LLTIAVKV-A TLPLTKKQVE STL----AMQ NLQPK----I KAIQQRYAG-

VPYAYGFAII LLTVFVKV-A TLPLTKKQVE STL----AMQ NLQPK----I KAIQQRYAG-

IPYSYGFAII ALTVLVKV-A TFPLTQKQVE STL----SLQ ALQPR----V KELQAKYAD-

VPYSYGYSIL LLTVLVKL-A TFPLSKQQVE SSI----QMQ AMQPR----I KELQAMYAN-

VPYSYGFSII VLTVLVKL-A TFPLSKKQVE SSM----QMQ AMQPR----I KELQAMYAN-

R-AGADSA-- ---------R YGQEMVLFMK EKNLNPL--K NMLVPLAQAP IFISFFMGLR

R-SGAESA-- ---------R YAQEMMLFMR EKGVNPL--K NMVVPLAQAP LFISFFMGLR

T-YAKKNWTE KEARIMYNHS LKKQWNNLIV RENCHPA--K TMVLLWGQIP LWIVQSVAIR

A-MAHKKWSE KQTQIVYRRS IKKQWQNLIV RDNCHPM--K TMIVLWGQIP LWIFQSVALR

-DT--DQ--- --------MR AMEKRRLIMK ENGVSTL--A TLFP-AVQLP LAYGFFQALR

L--RRQAKTM KYEQILLAKE ARKRQKELFA KNGVQLW--K NFILPAFQVP LWIMMSITMR

K-AGQEF--- --------YK ASSEMTFYQK KHDVKLF--R PLILPLTQAP IFISFFIALR

K-AGHEY--- --------YK ASSEMALYQK KHGIKLY--K PLILPVTQAP IFISFFIALR

V-VRNQKWSK RVARLTYLKN MRRLVSELYV RDNCHPF--K ATILVWIQFP MWIFMSVALR

V-VRNQGWSK RDARLTYLKN MRRLISELYV RDNCHPF--K ATVLVWIQLP MWIFMSFALR

-----DK--- --------DK IQKETSVLYE QAGVNPL--A GCLPTLATIP IFIGLFSSLT

-----DP--- --------KK AQLEIMSLYR ERGVNPA--S GCLPLLIQMP FLIGMFDLLK

-----EP--- --------KR AQMEVMALYK TNKVNPI--T GCLPLLIQLP FLIVMFDLLK

-----NP--- --------AK MQSELSRIYR EAGVNPV--G GCLPTLLQMP LLFAMFYVFR

-----NP--- --------QK MQSELGRIYK EAGVNPL--G GCLPVLLQMP LLFAMFYVFR

-----DP--- --------QK QQEELGKVMK EFG-NPL--S GCLPLLVQMP ILFALFATLR

-----DP--- --------QK QQEELGKVMK EFG-NPL--S GCLPLLVQMP ILFALFATLR

-----DP--- --------QK LREAQAKLYS ELGVNPL--G GCLPLLIQMP VLFALFATLR

GTQ--QQ--- --------MQ ISQLQMRVYK DNNMSMMGGM GCLPLLIQLP IMIGIYQAVA

GTQ--QQ--- --------MQ ISQLQMRVYK ENNMSMMGGM GCLPLLIQLP IMMGIYQAVA

-DV--SR--- --------QK LQEETSKLYK EAGINPY--T GCLPMLIQLP VMYALYQAIW

-DV--SR--- --------QK LQEETSKLYK EAGINPY--T GCLPMLIQLP VMYALYQAIW

-DT--SR--- --------TL LQQETNKLYK EAGVNPY--T GCLPVIIQLP VMYALYGAIL

GTP--QQ--- --------MT LSGWQRELYS KNQVSLTGGI GCLPLLIQLP IMWGIYQAVF

ENK--MK--- --------MR KQKEIADLYK KNNINVL--D GFLPQLVAMP IFFAMWRAIQ

KNK--MK--- --------AR QQQEVAELYK KHGINPL--D AFVTMIISLP VFIMMWRVIQ

-----DQ--- --------ER MNKEINRVYE ENGVNPL--A GCGPALLSFP VLAGLYRAFN

-----DQ--- --------ER MNKEINRVYE ENGVNPL--A GCGPALLTFP VLAGLYRAFN

GDP--RA--- --------AD KNQETMDLYK KHGVNPM--G GCLPMVLQIP FFFAFYKVFT

KDP--KR--- --------AD MNTEMAALYK QHSVNPV--G GCLPLVIQMP FLIAFYGMLA

-----DP--- --------KM RQQKTMELYK KNNVNPV--G GCLPMVIQIP IFIALYTAFS

-----NT--- --------QK IHEEMAKLYR EAQYNPL--S GCLPTLVQMP IIFAMYRLFN

-AN--TM--- --------QM VAHEKKKILK KYGISQM--K LFYP-MAMFP LTIGIFLGIR

-LFNNKAATA PQIEMLAMKE RRKRRVKLYK EHGCEMW--K SLIGPLVQLP IWITMSLAVR

-----DQ--- --------QK LMEETRKLQK EMGVNPV--A GCLPVLVQMP VFIGLFHVLR

-----DK--- --------ES ILEQQKEIQE EYGYSMA--A GCVPALIQVP VFLGLYQVLL

-----DK--- --------QR QSEEMMKLYK ETGTNPL--S SCLPILAQSP FFFALYHVLN

-----DK--- --------QR QSEEMMKLYK ESGTNPL--S SCLPILAQSP FFFALYHVLN

-----DK--- --------QQ MQLEIMKLQK EHG-NPL--L GCLPILLQIP LFISLFHVFT

-----DK--- --------TK QQQEMMALYK KHKINPA--A GCLPVLAQMP IFYALYKTLF

-----DK--- --------AK QQQEMMEIYR KEKINPV--A GCLPVLLQIP VFFSLYKVLF

-DQ--TQ--- --------QK LQQETMALFQ KHGVNPL--A GCFPILIQMP ILIGFYHAIM

-DA--TQ--- --------QK LQQEMMLLFQ KHGVNPM--A GCFPILIQMP ILIGFYHAIM

-DP--KQ--- --------KE LQMEMMKLYQ EHNINPLA-M GCLPMLIQSP IMIGLYYAIR

-DP--TQ--- --------RK LQQEMMQLYQ KHGVNPA--S GCLPVLIQMP IFMALYYAIS

-----DK--- --------QR LNQEMMKLYQ TEKVNPL--G GCLPILVQIP VFIALYWVLL

-----DR--- --------MK FQQAVMEMYK TEKVNPL--G GCLPMLIQIP VFIGLYWALL

-----DR--- --------EG MNKAVMELYR DHKVNPL--G GCLPMLVQIP VFFALYKALM

-----DR--- --------QR MNQEVMELYK TYKVNPA--G GCLPMLLQIP VFLGLYQGLL

-----DP--- --------SK LNAHVMELYK KNGANPM--G GCLPILIQIP IFFAIYRVLL

-----DP--- --------QK MNLHMMELYK KHGANPM--S GCLPILIQIP IFFAIYRVLL

-----DK--- --------AK ISQATMELYK QEKVNPL--G GCLPILIQIP VFIALYWVLL

-----DR--- --------QK YQQATMELFK KEKINPM--G GCLPLLIQMP IFFALYWVLV

-----DQ--- --------EK IQLETARLYK LAGINPL--A GCLPTLATIP VWIGLYRALS

-----DQ--- --------ER IQLETARLYK LAGINPL--A GCLPTLATIP VWIGLYRALS

-----DQ--- --------ER IQLETARLYK LAGINPL--A GCLPTLATIP VWIGLYRALS

-----DQ--- --------ER IQLETARLYK LSDVDPL--A GCLPTLVTIP VWIGLYRALS

-----NQ--- --------ER IQLETSRLYK QAGVNPL--A GCLPTLATIP VWIGLYQALS

-----NQ--- --------ER IQLETARLYK QAGVNPL--A GCFPTLATIP VWIGLYQALS

-----NQ--- --------ER IQLETSRLYR QAGVNPL--A GCFPTLATIP VWIGLYQALS

-----NQ--- --------ER IQLETSRLYR QAGVNPL--A GCFPTLATIP VWIGLYQALS

-----DP--- --------EN LQLETARLYK EAGVNPL--A GCFPTLATIP VFIGLYNALS

-----DP--- --------ER LQLEQARLYR EAGFNPL--A GCLPLFATLP VFIGLYRALS

-----DP--- --------ER LQMEQARLYK EAGFNPL--A GCLPVFATLP VFIGLYRALS

EMANTP---- -ESMRD-GGL FWFTDL-TIC DQF------- ---------- ----------

QMANAP---- -ESMRD-GGL FWFTDL-TMA DPF------- ---------- ----------

NLVSMP---- PTELTL-GGF GWIPNL-TEL DHS------- ---------- ----------

NLVYMP---- PTEMTI-GGF GWIPNL-TVV DNS------- ---------- ----------

KMANHN---- -EGFSD-QGY AWFQNL-IEV DPY------- ---------- ----------

DLSGTK---- -PSLYE-EGI LWFQDL-SIA DPM------- ---------- ----------

EMANLP---- -PSLQT-GGL LWFQDL-TLS DPT------- ---------- ----------

EMANLP---- -PSLQT-GGL WWFQDL-TVS DPI------- ---------- ----------

NFSTAA---- -EQLAT-DGI LWFPDL-TAL DST------- ---------- ----------

NLSTAA---- -EQLAT-GGI LWFPDL-TAP DST------- ---------- ----------

NVAN------ -DGLLT-GF- YFVPSL-AGP TTMMRQSGLG TSWLWP---- ----------

SSFA------ --LRGA-PFI GWIDDL-TAP DVLS------ --WSKP---- ----------

SSFL------ --LRGA-SFI GWIDNL-TAP DVLS------ --WTTP---- ----------

SSIQ------ --LRQH-GF- LWAKDL-SVP DSIDFGF--- ---AIP---- ----------

SSIE------ --LRQQ-SF- LWAKDL-SLP DSIDLGF--- ---SIP---- ----------

GSPFAVSDQI AAIEPK-PFT SFISETKHFP VIASLPSGTK LGVGDKAQIK TGESFSRLSG

GSPFAVSDQI AAIEPK-PFT SFISETKHFP VIASLQSGTK LGVGDKAQIK TGESFSRLSG

GSPFAVAEVA AEVVPT-PYV NFVTDSVHKP VVLVAPKGTK IAVGEQVQFR GGKPFQLVAE

YSKE------ --LAAS-SF- FGI-SL-GQR S--------- ---------- ----------

YSKE------ --LAAS-SF- FGI-SL-GQR S--------- ---------- ----------

RTPQ------ --LQNG-RF- LWM-DL-GRP D--------- -----P---- ----------

RTPQ------ --LQNG-RF- LWM-DL-GRP D--------- -----P---- ----------

RTPQ------ --LQTG-RF- LWM-DL-SKP D--------- -----P---- ----------

YSQE------ --LAHS-TF- FGI-SL-SQK S--------- ---------- ----------

AVPS------ --IKAT-TW- LGITSW-RAG NFIW------ ---------- ----------

SLPE------ --FKST-VW- LGLTSW-RGE WQYG------ ---------- ----------

NAGI------ -DGAFE-P-W FFLPSL-AGP TDA-----RD LSWLLP---- ----------

NAGI------ -DGAFE-A-W FFLPSL-AGP TDA-----RD LSWLLP---- ----------

VSVE------ --MRGA-PWL -WVSDL-SQP ETL------- -----P---- ----------

VAIE------ --LRQA-NW- FWLHDL-SGP D--------- -----H---- ----------

DTID------ --LWNS-P-- LWVKDL-SEP DVITS----P A---IP---- ----------

NYFE------ --FRGA-M-- IWIPDL-SLA DSVTLPFALP ---------- ----------

RMCEIG---- -QGLST-EG- LWFQNL-AAP D--------- -----P---- ----------

AMCGIV---- -KSMGT-EG- LWFPDL-LMM DHS------- ---------- ----------

SFNR------ --TGLG-SEV YIFSDV-Q-- -SFARLFGVP LSAFIS---- ----------

RMAR------ --PKEG-DTV HGM--L-TNV RSFTRFFGVP LPAYNS---- ----------

GIAS------ --GDTI-GKI --------LL ESAAHIFGAP LAAKFT---- ----------

GIAT------ --GKTI-GVI --------LL ASAAHIFGAP LAAKFT---- ----------

HLAP------ --MNEG-PGL VWRPRV-GQV EQIAKLWGIS LSNSFG---- ----------

VTIE------ --LRHE-PFL -YIPDL-SEQ DPTVFNL--- -FGLLP---- ----------

VTIE------ --MRHA-PFY GWIHDL-SAA DPTIFNL--- -FGLIP---- ----------

RTQA------ --ISEH-SF- LWF-DL-GEK D--------- -----P---- ----------

RTRE------ --IAQH-NF- LWF-DL-GEK D--------- -----P---- ----------

STPE------ --IASH-SF- LWF-SL-GQS D--------- ---------- ----------

RTQE------ --IKTH-SF- LWV-EL-GHR D--------- -----P---- ----------

GAVE------ --MRGA-PWI LWIKDL-ASA D--------- -----P---- ----------

ASVE------ --LRGA-PWV LWYTDL-ARP D--------- -----P---- ----------

FSIE------ --LRHA-PFY FWITDL-SGP DNLGQML--- ---GLP---- ----------

NAIE------ --LRHA-PFI VWLADL-SAK D--------- -----P---- ----------

NAIE------ --LKGA-PWI FWIKDL-AIM D--------- -----P---- ----------

NAIE------ --LKAA-PWA FWITDL-SVM D--------- -----P---- ----------

ESVE------ --LRQA-PFI FWINDL-ASA D--------- -----P---- ----------

ESVE------ --LRQA-PWL GWIQDL-TAR D--------- -----P---- ----------

NVAD------ -EGLLTEGF- FWIPSL-AGP TTVARQNGSG ISWLFP---- ----------

NVAN------ -EGLLTEGF- FWIPSL-AGP TTIERQNGSG ISWLFP---- ----------

NVAN------ -EGLLTEGF- FWIPSL-AGP TAISRQNGSG ISWLFP---- ----------

NVAN------ -EGLLTEGF- FWIPSL-AGP TTIARQSGQG ISWLFP---- ----------

NVAN------ -EGLFTEGF- FWIPSL-GGP TSIARQSGSG ISWLFP---- ----------

NVAN------ -EGLLTEGF- FWIPSL-GGP TTIARQSGAG ISWLLP---- ----------

NVAN------ -EGVLTEGF- FWIPSL-GGP TTIARQSGSG ISWLFP---- ----------

NVAN------ -EGVLTEGF- FWIPSL-GGP TTIARQSGSG ISWLFP---- ----------

NAAK------ -EGLLTEGF- FWIPSL-GGP TTIG----GG LEWLVP---- ----------

NAAA------ -EHLLDDGF- YWIPSL-GGP TSIARNDGNG FAWLWP---- ----------

NAAS------ -EGLLTDGF- YWIPSL-GGP TSISRNAGSG FAWLWP---- ----------

---------- ---------- ---------- ---------- ---------- ----------

---------- ---------- ---------- ---------- ---------- ----------

---------- ---------- ---------- ---------- ---------- ----------

---------- ---------- ---------- ---------- ---------- ----------

---------- ---------- ---------- ---------- ---------- ----------

---------- ---------- ---------- ---------- ---------- ----------

---------- ---------- ---------- ---------- ---------- ----------

---------- ---------- ---------- ---------- ---------- ----------

---------- ---------- ---------- ---------- ---------- ----------

---------- ---------- ---------- ---------- ---------- ----------

---------- ---------- ---------- ---------- ---------- LPDGAPPIGW

---------- ---------- ---------- ---------- ---------- --------IF

---------- ---------- ---------- ---------- ---------- --------VW

---------- ---------- ---------- ---------- ---------- LY--------

---------- ---------- ---------- ---------- ---------- LY--------

VEGGTKFTPT WSVTKGDDLV KVSADGTVQA LAEGDATQGK IPGLAAQSGF LIKALGQVGF

VEGGTKFTPT WSVTKGDDLV KVSADGTIQA LSEGDATQGK IPGLAAQSGF LIKALGQVGF

AGGDPLLRPT WKITKGEERA QIQPDGTLLA LQPGEVTEVA IPGLASDTGF LIDKLGRVGF

---------- ---------- ---------- ---------- ---------- I---------

---------- ---------- ---------- ---------- ---------- I---------

---------- ---------- ---------- ---------- ---------- Y---------

---------- ---------- ---------- ---------- ---------- Y---------

---------- ---------- ---------- ---------- ---------- Y---------

---------- ---------- ---------- ---------- ---------- V---------

---------- ---------- ---------- ---------- ---------- ----------

---------- ---------- ---------- ---------- ---------- ----------

---------- ---------- ---------- ---------- ---------- LADLAPPIGW

---------- ---------- ---------- ---------- ---------- LSDYAPPIGW

---------- ---------- ---------- ---------- ---------- ----------

---------- ---------- ---------- ---------- ---------- L---------

---------- ---------- ---------- ---------- ---------- ---------Y

---------- ---------- ---------- ---------- ---------- V---------

---------- ---------- ---------- ---------- ---------- Y---------

---------- ---------- ---------- ---------- ---------- ----------

---------- ---------- ---------- ---------- ---------- MKEMYQPVDF

---------- ---------- ---------- ---------- ---------- MPEQLAHLGT

---------- ---------- ---------- ---------- ---------- DASKVESLGA

---------- ---------- ---------- ---------- ---------- DADKVTQLGA

---------- ---------- ---------- ---------- ---------- SQESLDFLGA

---------- ---------- ---------- ---------- ---------- FDPTTVPLGS

---------- ---------- ---------- ---------- ---------- YDPTQIPVGY

---------- ---------- ---------- ---------- ---------- Y---------

---------- ---------- ---------- ---------- ---------- Y---------

---------- ---------- ---------- ---------- ---------- I---------

---------- ---------- ---------- ---------- ---------- Y---------

---------- ---------- ---------- ---------- ---------- Y---------

---------- ---------- ---------- ---------- ---------- Y---------

---------- ---------- ---------- ---------- ---------- F---------

---------- ---------- ---------- ---------- ---------- F---------

---------- ---------- ---------- ---------- ---------- Y---------

---------- ---------- ---------- ---------- ---------- W---------

---------- ---------- ---------- ---------- ---------- Y---------

---------- ---------- ---------- ---------- ---------- Y---------

---------- ---------- ---------- ---------- ---------- FIEGHPPLGW

---------- ---------- ---------- ---------- ---------- FVDGQPPLGW

---------- ---------- ---------- ---------- ---------- FVDGHPPLGW

---------- ---------- ---------- ---------- ---------- FTDGHPPLGW

---------- ---------- ---------- ---------- ---------- FVDGHPPLGW

---------- ---------- ---------- ---------- ---------- FVDGHPPLGW

---------- ---------- ---------- ---------- ---------- FVDGHPPLGW

---------- ---------- ---------- ---------- ---------- FVDGHPPLGW

---------- ---------- ---------- ---------- ---------- FENGAPPVGW

---------- ---------- ---------- ---------- ---------- FVDGHPPLGW

---------- ---------- ---------- ---------- ---------- FVDGHPPLGW

-------YAL PIITSLTLFA TIEL-GTDA- --MANM-QTA KYILRALPLF IFPF--TINF

-------YLL PLITSATLYL TIEI-GTDA- --LANM-NTM KYVLRALPIV IFPF--TMNF

-------LIF PVALGVINLS IIEI-QAAR- -KPKLQ-TIF TNLFRGLSIL MVPI--AASV

-------YIL PVALGLINLA IIEV-QAMR- -RSRLQ-NIA NNVFRGLSVV MVPV--ACTV

-------LGL QAISAAAIIA VVRV-GGEG- --QAMA-AGM KKVMTVVPIA SIFI--TKGF

-------HVF PVILGITALC NIEWT-LKTK FRPTL-TDAF GNLTKMSIVF MMAI--SLHA

-------YIL PLVVTATMWG VLEL-GAEG- --MSDL-QWM RNLIRVMPLA VLPI--TIHF

-------YIL PLAVTATMWA VLEL-GAEG- --VSDL-QWM RNVIRMMPLI TLPI--TMHF

-------WIL PISVGVINLL IVEI-FALK- -GMRFQ-TYI TYFVRAVSVL MIPV--AATV

-------WIL PISVGVINLL IVEI-CALK- -GMRFQ-TYI TYFVRAMSVL MIPI--AATV

EDAAAY-LTL PLLLVAVQYA SSSVT-SP-- PDPKENANTQ RALLVFLPLM VGWF--SLNV

FIGTEF-HLL PILLGLVMFL QQRF-MSS-- PSMDQQ-RQQ RAMGTMMTVV FAVM--FYNF

FLGNEF-HLL PILLGIVMFA QQKI-SASK- PATDQQ-RQQ ETMGTMMALL FTFM--FYNF

---GDI-AFF PILMAGTVYL QQKI-TPT-- ---APN-EQM KIMLVLFPVM MLFF--FNNM

---GDI-ALF PILMGGAVYL QQKI-TPT-- ---ASN-DQM KAMLYIFPVM MLLF--FNNL

YDGENWDAIL VGGFGLTLLV SQIL-SG--- ----RN-PQQ STANKITPVM ITGMFLFFPL

YDGENWDAIL VGGFGLTLLV SQIL-SG--- ----RN-PQQ STANKITPVM ITGMFLFFPL

DDGTHWDIGM IVLFGVSIYL NQSL-TN--- ----AD-PSQ SSMARITPVL FSAMFLFFPL

--------VL TIIATLLYVI QGYLS-MV-- -IPEQK-KAM QMTLILSPAM TFFI--SISA

--------IL TIIATLLYVV QGYLS-MV-- -IPEQK-KAM QMTLILSPAM TFFI--SISA

--------IM PVLAAAFTFI STYIS-QM-- --PQQN-GMT KGMTYLMPLI IGVS--AIGI

--------IM PILAAVFTFM STYIS-QM-- --PQQN-GMT KGMTYLMPLI IGVS--AIGI

--------IM PILAMVFTFL STYIS-QL-- --PKQN-GMT KVMMYGMSIM VGVM--ALNF

--------VL AIIATVFTVI QGYI-STI-- -IPEQK-KTM QSMMLFNPIM TLFF--SLSF

----II-IVV VIIQFTSQLI PHWL-NKKRT TEVQAL-KKS ERYQTIFIIV FTFM--AIIF

----LL-VVV AAVQGVAQFL PQIL-NRKKT SEEKAL-KKA NRTQRIMTIV FFFI--TLIF

DDASLY-LLF PIMTTLSQFV SMEVL-KPE- -EKKEM-QNQ SVLLKLLPFF IGYI--SLTV

EEASLY-LIF PVLTTISQFV SMEVL-KPE- -EKDEM-KNQ SVLLKLLPLF IGYI--SLTV

-----I-KIL PLVMIASQFV MQRM-TP--- ----QD-PAQ QKMMMFMPLV FGFM--FYNF

--------II PVLIVASTIL MQRM-TP--- ----QD-PQQ KMMTIMMPAF LGWI--SLRY

FQTIGL-NLL ALLMVGTQVF QTRM-TS--- ----VD-PNQ KMLMYVMPVM MLYI--FWNM

-TQTQM-RML PVLYVVSQIM FSKLT-QV-- ----HT-EQQ KIMTYVMPLF FFFF--FYDA

-------LGL QVITAAMYMA SIRL-GSE-- ---TLS-PGM KKILQWAPWI SVPF--LMKM

-------GVL PAAVGIITLL NVEL-TTKAE -GPKLP-KMM ANFARVGAIA LFSI--AAQT

TRDALV-APL IVIIVLATHF NARLSDR--- --QQMD-MMN RMMLWFMPLT ILFT--GVLW

DQIHA--FVL PLIIAACVFT TIVS-TRT-- -AVGMY-RFL IVMVLVAPIG LLQT--GIFI

SLDRVI-AVM IVLMSASQFF TQRQLTKN-- -KTPFM-QQQ KMLMYVFPVM FAVF--GINF

TLDRVV-AIM IVLMSASQFY TQRQLTKN-- -KTPFM-QQQ KMLMYVFPVM FAIF--GINF

NGHKIL-VVL IVLMGATTFF TQRQIART-- -DPQQA-MVQ KILLYGSPLM LAIF--GFRF

---LGI-GIL PLLMGAAMFV QLKL-NP--- ----PD-PTQ RMIFGLMPLI FMFI--FAPF

---LVL-GIW PIIMGITMWV QMKL-NP--- ----AD-PTQ QMIFAWMPLL FTFM--LASF

--------IL PIVAGVATFV QQKL-MMA-- ----GN-PQM AMMLWIMPIM IIVF--AINF

--------IL PVIAGVTTFI QQKI-MMA-- ----GN-PQM AMMLWMMPIM IVIF--AINF

--------LM SLSAGVQAYI AQKL-SKY-- -APQAQ-QSA KLMVFIFPVM MTIF--SLNV

--------IL PVLAALTTFI SLRL-SPS-- ----MM-PQM AMMLYIMPVM IFIG--ASSV

--------IL PVIMMVSMFV QTKL-NP--- ----TD-PIQ AKVMMMMPLI FGFM--FFWF

--------IL PVIMAATMFL QTFL-NP--- ----PD-PMQ AKMMKIMPLA FSAL--FFFF

-----V-GPL PLLMGATMFI QQKM-TP--- ----SD-PMQ AKMMLALPVV FTFM--FLNF

--------IT PVVMGATMLL QQRL-TP--- ----AD-PTQ AKIMMFMPVV FTFM--FLNF

--------IL PVLMGATMFI QQKI-TP--- ----AD-PMQ EKIMKFLPLI FTFF--FVTF

--------IL PIFMGLTMFI QQLI-TP--- ----MD-PMQ EKIMKFLPLI FTFF--FLTF

--------VL PLIMGATMLI QQKL-NP--- ----AD-PMQ AKVMMFLPIL FTGL--FWNF

-----F--IL PVLNIAIMWA TQKL-TP--- ----TD-PMQ AKMMQFMPLV FGVM--MAFM

PDTLAY-LVL PLLLVFSQYL SIQIM-QSS- -SNPAMKS-S QAVTKLLPLM IGYF--ALSV

SDTVAY-LVL PAMLVVLQYM SVQIM-QSS- -SDPNVKN-S QAIMKFLPLM IGYF--SLSV

SDTVAY-LVL PVMLVVSQYI SVQIM-QSS- -SDPNVKN-S QAITKFLPLM IGYF--SLSV

SDTLAY-LVL PVLLVISQYV SSQVM-QPP- -NNPSQQG-A QAVVKFLPLL IGYF--ALSV

YDTVAY-LVL PVLLIASQYV SMEIM-KPP- -TDPAQKN-T LLVFKFLPLM IGYF--ALSV

HDTICY-LVL PVLLVASQFV SMEIM-KPP- -TDPSQKN-T LLVLKFLPFM IGWF--SLSV

HDTAAY-LVL PVLLIASQYV SMEIM-KPP- -TDPTQKN-T LLVFKFLPIM IGYF--SLSV

NDTAAY-LVL PVLLVVSQYV SMEIM-KPP- -TDPTQKN-T LLVFKFLPLM IGYF--SLSV

ANAAAY-LVM PVLLVASQYA SQKII-SSQ- -NQPSQQQ-A QAILKFLPLM IGWF--SLNV

YETGCY-LVL PVLLVVSQFV SQTII-SPQ- -TDPAQQQ-S QAILKFLPFM IGFF--SLNV

HDTTAY-LVL PVLLVASQYV SQQIV-SPQ- -TDPAQQQ-S QAILKFLPLM IGFF--SLNV

PGAILCYWAC SNFFSLVQVG FLRIPKVRDF FKIDRIVT-- ---------- --KPETNT

PAAILTYWAC SNFISLGQVA VLRIPSVREY FKIEKMLT-- ---------- --APSDNG

PSCLCLYWVT SSAYGLGQNL LLLSPRVRRT VGIPAVPS-- ---------- --T-HS-T

PSALCVYWVA SSSFGLAQNL LILSPEVRRS VGIPKTQT-- ---------- --S-ELKK

ASAIILYFAV NSIFSLIQSS LFKSSWFRKI AGMPPKLS-- ---------- -----LAH

PAALTIYWIS SQLYSLLQNV MMDLMLPISF TPKKRIN--- ---------- -------N

PTAVFMYWLS SNMFSLGQVA CLRIPAVRTI LKIPQRVV-- ---------- --DSNKNG

PTAVFMYWLS SNLFSLVQVS CLRIPAVRTV LKIPQRVV-- ---------- --DLDKNG

PSSIVLYWLC SSLMGLSQNL LLRSPRFRQL CRIPLTKS-- ---------- ------DK

PSSIVLYWLC SSFVGLSQNL LLRSPGFRQL CRIPSTKS-- ---------- ------DK

PAGLSLYYLA NTVLSSAIQI YLKKLGGANV VMNELG---- ---------- --PVTKPA

PSGLNIYWLS SMLLGMVQQW FVSKQIQKTP NPIAVKT--- ---------- -------R

PSGLNIYWFS SMLLGLIQQW FTNKILDSKH LKNEISV--- ---------- -------R

PAGLGLYYLM FNIFSVAQQF YINKTTTADD MPKVNLA--- ---------- -------K

PAGLGLYYLM FNVFSIAQTF YINKTSSADD LPKISLE--- ---------- -------Q

PAGVLLYMVI ANIFQAGQTF LLSREALPEN LQKILND--- ---------- -------K

PAGVLLYMVI ANIFQAGQTF LLSREALPEN LQKILNE--- ---------- -------K

PAGVLLYILV SNIFQTVQTF LLSREPLPEN LQQLVEE--- ---------- -------P

PGALALYFLV GGLIAILQQV ITTFIIMPKV KKDVAAE--- ---------- -------D

PGALALYFLV GGLIAILQQL ITTFVIMPKV KRDVAAE--- ---------- -------D

QSAISLYWVI SNLFQVVQTF ILQNPFKYQR ELEEQKK--- ---------- -------K

QSAISLYWVI SNLFQVVQTF ILQNPFKYQR ELEEQKK--- ---------- -------K

QSAITLYWVI SNLFQAVQTF ILQNPIKYRK EQEAKNE--- ---------- --------

SGALALYWAA GNFVMIIQQL IVTFILTPKV KQHVADE--- ---------- -------K

TAGVQVYWLF TGIWQTFQVI GLHKLRKTQW FKNKYSR--- ---------- -------A

SAGLQVYWII SGIWTIIQTL SIHKFKKSAY YRRKYLD--- ---------- -------A

PAGLALYWFW NNVFTTGIQV YLRNGGA--- ---------- ---------- --------

PAGLALYWFW NNVFTTGIQV FLRNGGAKAT VE-------- ---------- --------

PSGLVLYYLT SNLVSMGQQW FFNKTSMATL AAESIAP--- ---------- -------K

ASGLGLYWIV GNIIGFAQQF IMNKTELGRE IAAVRAK--- ---------- -------E

PSGVTLYWTF QNVLSIGQQW ILKKTEEKKK AKV------- ---------- --------

PSGLLVYWTA MNGVTLVQQL VMKRTANKNK T--------- ---------- --------

PAALLLHFFV NGILMLIQGV ALRNPFFRKK LGIHEIV--- ---------- -------K

PTAVCLYWIS SSGFSLIQNV LLNKYMPLRE EPPMFTI--- ---------- -------H

HLGLLFYMVS NNIWTFFQQR WIFDKIDAEI EAKKAAK--- ---------- -------K

PAAICLYWVA NNLWTLIQNN GMFLALWYPY DSDHKAR--- ---------- RLIQPWKD

PVGVLVYWLT TNVWTMGQQM YVIRNNPTPG SKAQASYLRN NKAGLAAQAD GNVVKNDK

PVGVLVYWLT TNVWTMGQQM YVIRNNPTPG SKAQAAFLRN TKAGLAAQSD GTIGKGEK

PIAVLLYWLT TNLWSMGQQF FVIKKMPPLK PLGAGGVVAG SAERVSSAPP GTRSRSGR

AAGLVLYWFW NTFLSVIQQY IIMKRQGADV DILGNIK--- ---------- -------A

PAGLVIYWAW NNLLSVIQQG YIMRKNGVKV ELFDNLK--- ---------- -------T

PAALSLYWVV GNLFMIAQTF LIKGPDIKKN PEPQKAG--- ---------- -------K

PAALSLYWVV GNIFSIVQTY LIKGPEIAAH S-----G--- ---------- -------K

PAALPLYWFT SGLFLTVQNI VLQMTHHKSK KTAALTE--- ---------- --------

PSALSLYWVV GGCFSIIQSL ILRSQLKAAK AAENS----- ---------- --------

PAGLVLYWVV NNVLSIAQQW QITRLIDAGG KAANDAK--- ---------- -------A

PAGLVLYYVV NNVLSMAQQW FINKQIEKSN KAALQS---- ---------- --------

PSGLVLYWLL NNILTIGQQM YINKLVND-- ---------- ---------- --------

PAGLVVYWLV NNVLSIGQQW WMLRKS---- ---------- ---------- --------

PAGLTLYWFI NNLCSVAQQL VVNKIFKKQK EQAIMEK--- ---------- -------R

PAGLTLYWCV NNICSLIQQV IVNKLFKNHK KEEIAKH--- ---------- -------S

PSGLVLYWIV NNTLSILQQW YITRKYSDEK PAKKVVA--- ---------- -------K

PAGLVLYWVV NGGLGLLIQW WMIRQHGEKP SKIIQAN--- ---------- -------K

PSGLSLYWLT NNILSTAQQV WLQKYGGAKN PVEKFTNLP- ---------- GCPKPGER

PSGLSLYWLT NNILSTTQQV WLQKLGGAK- ---------- ---------- --------

PSGLSLYWLT NNILSTAQQV WLQKSGGAKN PMMKSSDD-- ---------- --------

PSGLSLYWLT NNILSTAQQV WLQKLGGAKN PVKEYIDKG- ---------- SGPQRGEK

PSGLSIYWLT NNVLSTAQQV YLRKLGGAKP NMDENA-SK- ---------- ISADAGEV

PSGLSIYWFT NNILSTAQQV WLRKLGGAKP VVNQGG-SG- ---------- ITAQPGEQ

PSGLSIYWFT NNVLSTAQQV WLRKLGGAKP VVNENA-SG- ---------- ITAQPGDV

PSGLSIYWFT NNVLSTAQQV WLRQLGGAKP VVNENA-SG- ---------- ITAQPGDV

PSGLTLYWFV NNLLSTGQQL YLKATVKVNI PEAIKAPA-- ---------- ---ERVKA

PAGLTLYWFF NNIITTAQTV VLRKITKPIE VPTGA--G-- ---------- ---EYVPN

PAGLTLYWFA NNIITTAQTL ILRKTTTAPE PPVAG----- ---------- --------
