# Supplementary material for: The evolution of YidC/Oxa/Alb3 family in the three domains of life: a phylogenomic analysis
Source: BMC Evol Biol. 2009 Jun 18;9:137. doi: 10.1186/1471-2148-9-137 (PMC2706819; doi:10.1186/1471-2148-9-137)
Supplement: Additional file 5 — Maximum-likelihood phylogenetic tree of representative bacteria and eukaryotes YidC/Oxa/Alb3 protein sequences. The tree is based on alignment of the full sequences. The nodes with bootstrap support values more than 50% were indicated. [file 1471-2148-9-137-S5.pdf]

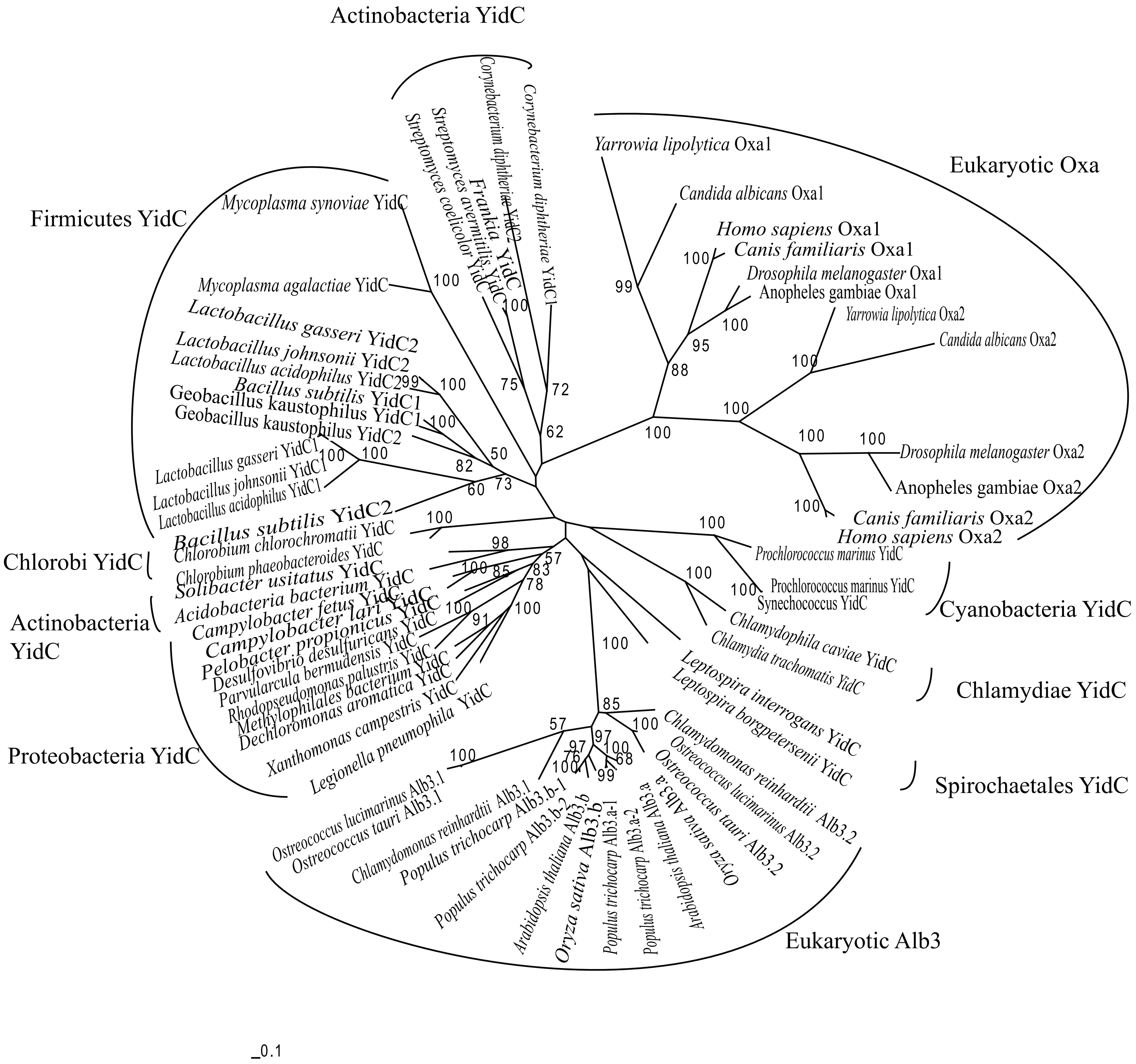
Actinobacteria YidC
Corynebacterium diphtheriae YidC2
Corynebacterium diphtheriae YidC1
Frankia YidC
Streptomyces avermitilis YidC
Streptomyces coelicolor YidC
Eukaryotic Oxa
Yarrowia lipolytica Oxa1
Candida albicans Oxa1
Homo sapiens Oxa1
Canis familiaris Oxa1
Drosophila melanogaster Oxa1
Anopheles gambiae Oxa1
Yarrowia lipolytica Oxa2
Candida albicans Oxa2
Drosophila melanogaster Oxa2
Anopheles gambiae Oxa2
Canis familiaris Oxa2
Homo sapiens Oxa2
Firmicutes YidC
Mycoplasma synoviae YidC
Mycoplasma agalactiae YidC
Lactobacillus gasseri YidC2
Lactobacillus johnsonii YidC2
Lactobacillus acidophilus YidC2
Bacillus subtilis YidC1
Geobacillus kaustophilus YidC1
Geobacillus kaustophilus YidC2
Lactobacillus gasseri YidC1
Lactobacillus johnsonii YidC1
Lactobacillus acidophilus YidC1
Bacillus subtilis YidC2
Chlorobi YidC
Chlorobium chlorochromatii YidC
Chlorobium phaeobacteroides YidC
Actinobacteria YidC
Solibacter usitatus YidC
Acidobacteria bacterium YidC
Campylobacter fetus YidC
Campylobacter lari YidC
Pelobacter propionicus YidC
Desulfovibrio desulfuricans YidC
Parvularcula bermudensis YidC
Rhodopseudomonas palustris YidC
Methylophilales bacterium YidC
Dechloromonas aromatica YidC
Xanthomonas campestris YidC
Legionella pneumophila YidC
Proteobacteria YidC
Prochlorococcus marinus YidC
Prochlorococcus marinus YidC
Synechococcus YidC
Cyanobacteria YidC
Chlamydophila caviae YidC
Chlamydia trachomatis YidC
Chlamydiae YidC
Leptospira interrogans YidC
Leptospira borgpetersenii YidC
Spirochaetales YidC
Chlamydomonas reinhardtii Alb3.2
Ostreococcus lucimarinus Alb3.2
Ostreococcus tauri Alb3.2
Oryza sativa Alb3.a
Arabidopsis thaliana Alb3.a
Populus trichocarp Alb3.a-2
Populus trichocarp Alb3.a-1
Oryza sativa Alb3.b
Arabidopsis thaliana Alb3.b
Populus trichocarp Alb3.b-2
Populus trichocarp Alb3.b-1
Chlamydomonas reinhardtii Alb3.1
Ostreococcus tauri Alb3.1
Ostreococcus lucimarinus Alb3.1
Eukaryotic Alb3
0.1
